# Evaluation of the Local Anesthetic Activity, Acute Toxicity, and Structure–Toxicity Relationship in Series of Synthesized 1-Aryltetrahydroisoquinoline Alkaloid Derivatives In Vivo and In Silico

**DOI:** 10.3390/molecules28020477

**Published:** 2023-01-04

**Authors:** Azizbek A. Azamatov, Sherzod N. Zhurakulov, Valentina I. Vinogradova, Firuza Tursunkhodzhaeva, Roaa M. Khinkar, Rania T. Malatani, Mohammed M. Aldurdunji, Antonio Tiezzi, Nilufar Z. Mamadalieva

**Affiliations:** 1 Institute of the Chemistry of Plant Substances, Academy of Sciences of the Republic of Uzbekistan, Mirzo Ulugbek Str. 77, Tashkent 100170, Uzbekistan; 2 Department of Organic Chemistry, Faculty of Chemistry, National University of Uzbekistan Named after Mirzo Ulugbek, University Str. 4, Tashkent 100174, Uzbekistan; 3 Department of Pharmacy Practice, Faculty of Pharmacy, King Abdulaziz University, Jeddah 21589, Saudi Arabia; 4 Department of Clinical Pharmacy, College of Pharmacy, Umm Al-Qura University, Makkah 21955, Saudi Arabia; 5 Department for the Innovation in Biological, Agro-Food and Forestal Systems, Tuscia University, 01100 Viterbo, Italy

**Keywords:** health care, drug discovery, Pictet–Spengler reaction, 3,4-dimethoxyphenylethylamine, 1-aryl-6,7-dimethoxy-1,2,3,4-tetrahydroisoquinoline, ADMET/TOPKAT, local anesthetic activity, acute toxicity

## Abstract

Isoquinoline alkaloids constitute one of the most common classes of alkaloids that have shown a pronounced role in curing various diseases. Finding ways to reduce the toxicity of these molecules and to increase their therapeutic margin is an urgent matter. Here, a one-step method for the synthesis of a series of 1-aryl-6,7-dimethoxy-1,2,3,4-tetrahydroisoquinolines was performed in 85–98% yield by the Pictet–Spengler reaction. This was accomplished using the reaction between 3,4-dimethoxyphenylethylamine and substituted benzaldehydes boiling in trifluoroacetic acid. Furthermore, 1-(3′-amino-, 4′-aminophenyl)-6,7-dimethoxy-1,2,3,4-tetrahydroisoquinolines were obtained in 94% and 97% yield by reduction in 1-(3′-nitro-, 4′-nitrophenyl)-6,7-dimethoxy-1,2,3,4-tetrahydroisoquinolines with SnCl_2_ × 2H_2_O. The structures of the substances obtained were confirmed by infrared (IR) and nuclear magnetic resonance (^1^H and ^13^C NMR) spectra. ADMET/TOPKAT in silico study concluded that the synthesized compounds exhibited acceptable pharmacodynamic and pharmacokinetic properties without carcinogenic or mutagenic potential but with variable hepatotoxicity. The acute toxicity and structure–toxicity relationship (STR) in the series of 20 derivatives of 1-aryl-6,7-dimethoxy-1,2,3,4-tetrahydroisoquinolines (**3a**–**r**, **4a**, **b**) was studied via determination of acute toxicity and resorptive action in white mice employing intragastric step-by-step administration. The first compound, 1-phenyl-6,7-dimethoxy-1,2,3,4-tetrahydroisoquinoline hydrochloride (**3a**), showed the highest toxicity with LD_50_ of 280 mg/kg in contrast to 1-(3′-bromo -4′-hydroxyphenyl)-6,7-methylenedioxy-1,2,3,4-tetrahydroisoquinoline hydrochloride (**3e**) which proved to be the safest of the compounds studied. Its toxicity was 13.75 times lower than that of the parent compound **3a.** All compounds investigated showed high local anesthetic activity on rabbit eyes in the concentrations studied. Only **3r**, **3n**, and **4a** caused eye irritation and redness. All investigated derivatives (except **4b**) in 1% concentration were more active than lidocaine, providing longer duration of complete anesthesia. Therefore, based on the obtained results of in silico tests, local anesthesia, and acute toxicity, a conclusion can be drawn that the experimental compounds need further extensive future investigations and possible modifications so that they can act as promising drug candidates.

## 1. Introduction

Isoquinoline alkaloids are one of the common classes of alkaloids. They are biosynthesized from tyrosine and phenylalanine and basically include isoquinoline or tetrahydroisoquinoline ring as the main structural part in their skeleton [1]. However, the isoquinoline alkaloids do not constitute a homogeneous group of structurally related compounds due to the various levels of oxygenation, distribution, and intramolecular rearrangements along with the additional existence of rings attached to the base system. They could be divided into eight subgroups: aporphine, phthalideisoquinoline, benzylisoquinoline, benzo[*c*]phenanthridine, protopine, emetine, protoberberine, and morphinan alkaloids [2]. Meanwhile, isoquinoline alkaloids have recently received great attention as they constitute the genetic precursors of a plethora of naturally occurring compounds that have shown a pronounced role in the treating of various diseases [3]. The 1,2,3,4-tetrahydroisoquinoline derivatives have a wide range of biological activities, including antidepressant [4], immunomodulatory [5], anti-Alzheimer and anti-Parkinson [6], inhibiting NO production [7], anti-HIV [8], as well as antifungal [9] activity.

Aporphine alkaloids such as boldine, magnoflorine, glaucine, and oxoglaucine have demonstrated potent anti-inflammatory activity in various in vitro models, including anti-inflammatory activity by inhibiting cyclooxygenase (COX) enzymes, inhibiting prostaglandin synthesis, and downregulating the expression of proinflammatory cytokines (TNF-, IL-6) [10]. Furthermore, recent studies have suggested its neuroprotective effects in ischemic brain damage in mice, and anti-inflammatory activity in colitis models by downregulation of proinflammatory cytokines production [11]. In addition, tetrandrine possesses calcium-antagonistic and hypotensive effects [12]. Neferine improved cell viability and mitochondrial function and reduced cell apoptosis and the production of reactive oxygen species in LPS-treated H9c2 cells. In addition, neferine significantly upregulated Bcl-2 expression and suppressed cleaved caspase 3 activity in LPS-induced mouse heart tissue and H9c2 cells [13]. Selective antagonists of the orexin 1 (OX1) receptor have been proposed in the range of 6- and 7-substituted tetrahydroisoquinolines [14]. Noscapine is a phthalideisoquinoline alkaloid isolated from the opium poppy, *Papaver somniferum*. It has long been used as an antitussive agent, but has more recently been found to possess microtubule-modulating properties and anticancer activity [15]. The vasodilator mechanism of protopine is related to decreasing effects on [Ca^2+^]_i_ and increasing effects on cAMP and cGMP, as well as its influence on the PKC, has been proposed [16]. The 1-aryl-6,7-dihydroxy tetrahydroisoquinoline shows significant affinity towards the D_2_ receptor, with a K_i_ value of 31 nM. This significant affinity can be attributed to the presence of a thiomethyl group, and it is the most active 1-aryl-6,7-dihydroxy tetrahydroisoquinoline derivative reported to date [17]. Several 1-aryl-6,7-dimethoxy-1,2,3,4-tetrahydroisoquinoline derivatives displayed potent anticonvulsant effects in different animal models of epilepsy [18]. In addition, 1,2,3,4-tetrahydroisoquinoline (THIQ) and aryl-substituted derivatives of THIQ are potent inhibitors of the enzyme that catalyzes the formation of epinephrine-phenylethanolamine N-methyltransferase [19].

(-)-Stepholidine (SPD), a natural product isolated from the Chinese herb *Stephania*, possesses dopamine (DA) D1 partial agonistic and D2 antagonistic properties in the nigrostriatal and mesocorticolimbic DAergic pathways [20].

Several THIQ based natural products have been previously explored for their antitumor properties, making it a vital scaffold for anticancer drug design. Various medicinal chemistry strategies employed for the design and development of THIQ analogs as inhibitors or modulators of relevant anticancer targets, the common strategies employed for the synthesis of the core scaffold are also used [21].

Tetrahydroisoquinolines, which belong to a group of cyclized condensation adducts of biogenic amines with aldehydes, are referred to as mammalian alkaloids. They include salsolinol and tetrahydropapaveroline (THP) that are derived from dopamine through condensation with acetaldehyde and dopaldehyde (3,4-dihydroxyphenylacetaldehyde), respectively. THP is a putative dopaminergic neurotoxin that is implicated in the pathology of Parkinson’s disease [22]. In [23], the authors exclude possibility that salsolinol under physiological conditions could be an endogenous factor involved in the neurogenerative processes; conversely, it can exert a protective action on nerve cells in the brain.

Several tetrahydroisoquinolines were tested for their in vitro and in vivo capacities to modulate prolactin and β-endorphin secretion by the rat pituitary and for their abilities to displace [^3^H]spiroperidol and [^3^H]naloxone binding from pituitary and hypothalamic membranes. Authors classified etrahydroisoquinolines as having (a) higher affinity for opiate receptors (tetrahydropapaverine, papaverine, 6-methylsalsolinol, 1-carboxysalsolinol and 3’,4’-deoxynorlaudanosolinecarboxylic acid), (b) higher affinity for the dopamine receptor (salsolinol and 7-methylsalsolinol), or (c) approximately equal affinity for the two binding sites (6,7-dimethylsalsolinol and tetrahydropapaveroline, THP) [24]. 

In our previous work, we demonstrated that 1-aryltetrahydroisoquinoline derivatives possess neuroleptic [25], cardioprotective [26], antiarrhythmic, anti-inflammatory [27,28,29], and cytotoxic activities [30]. 

Continuing the search for biologically active substances in the series of 1-aryltetrahydroisoquinolines, the process was optimized, and six more new derivatives were obtained in addition to the already known ones.

The aim of this study is the search for promising local anesthetic substances with low toxicity. Finding ways to reduce the toxicity of these molecules and increase their therapeutic margin is an urgent matter. In an earlier study conducted by the authors, a number of 1-aryl-6,7-dimethoxy-1,2,3,4-tetrahydroisoquinoline derivatives were synthesized in 35–86% yield by Pictet–Spengler reaction from 3,4-dimethoxyphenylethylamine (**1**) and substituted benzaldehydes in two steps via imine production by boiling in benzene with apparatus Dean–Stark additive [31]. Pictet–Spengler reaction is a chemical reaction mode in which the condensation of β-arylethylamine with ketone or aldehyde occurs and consequently ring closure is achieved. Continuing our study, a more efficient one-step method was adopted to obtain 1-aryl-6,7-dimethoxy-1,2,3,4-tetrahydroisoquinolines (**3a**–**r**) in 85–98% yield by boiling in trifluoroacetic acid. Furthermore, the reduction of 1-(nitrophenyl)-6,7-dimethoxy-1,2,3,4-tetrahydroisoquinolines was also performed to obtain compounds **4a, b**.

In addition, the ‘structure–toxicity’ relationship in the series of 20 derivatives of 1-aryl-6,7-dimethoxy-1,2,3,4-tetrahydroisoquinoline (**3a**–**r**, **4a, b**) was also performed in vivo by determination of acute toxicity and resorptive action in white mice by stepwise intragastric administration. This was carried out with the aim of determining the value of introducing multiple functional groups onto the original compound (**3a**) in reducing its toxicity. Moreover, the synthesized series of 1-aryltetrahydroisoquinoline derivatives underwent absorption, distribution, metabolism, excretion, and toxicity (ADMET) prediction and toxicity prediction using computer assisted technology (TOPKAT) by Discovery Studio 2016 (Accelrys Inc., San Diego, CA, USA) in order to determine the pharmacokinetic, pharmacodynamic, and toxicity characteristics.

## 2. Results and Discussion

**Scheme 1:**
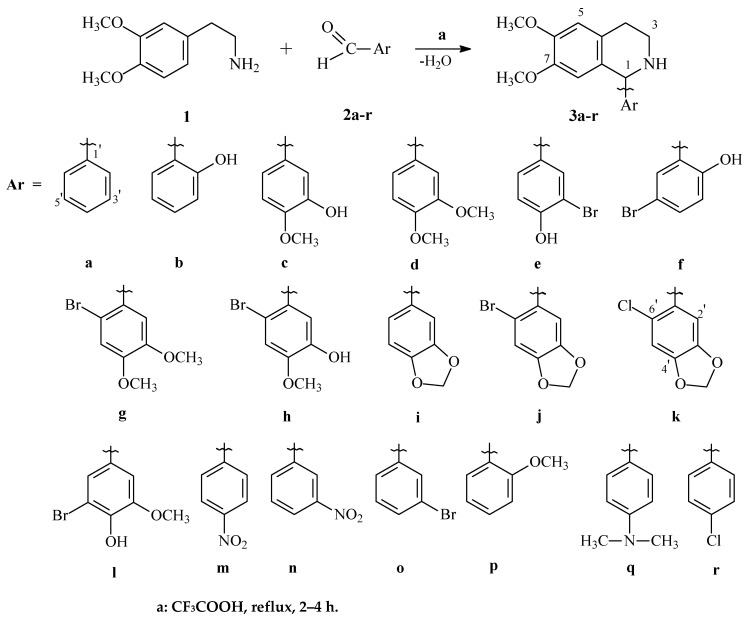
Scheme showing one-step synthesis of 1-aryl-6,7-dimethoxy-1,2,3,4-tetrahydroisoquinolines (**3a–r**).

A number of 1-aryl-6,7-dimethoxy-1,2,3,4-tetrahydroisoquinoline derivatives in yield of 35–86% were previously synthesized by the authors using the Pictet–Spengler reaction from 3,4-dimethoxyphenylethylamine (**1**) and substituted benzaldehydes in two stages obtaining imine by boiling in benzene with Dean–Stark apparatus [31]. In continuation of these studies, a more effective one-step method for the production of 1-aryl-6,7-dimethoxy-1,2,3,4-tetrahydroisoquinolines (**3a**–**r**) in 85–98% yield of boiling in trifluoroacetic acid was developed. Scheme 1 shows the one step synthesis of 1-aryl-6,7-dimethoxy-1,2,3,4-tetrahydroisoquinolines (**3a**–**r**).

**Scheme 2:**
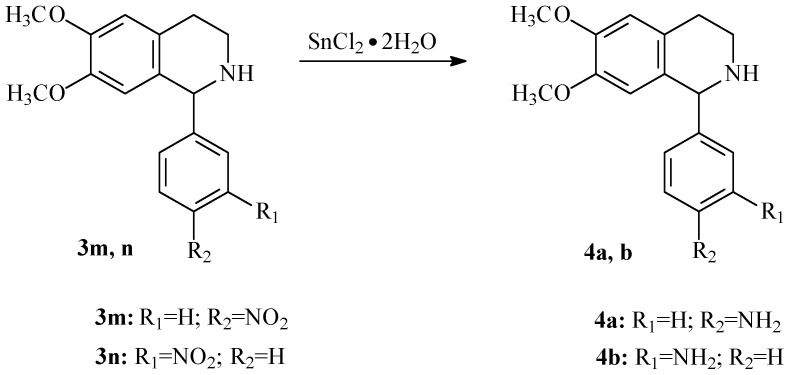
Scheme showing the reduction of 1-(4′-nitrophenyl)- (**3m**), 1-(3′-nitrophenyl)-6,7-dimethoxy-1,2,3,4-tetrahydroisoquinolines (**3n**) to form **4a** and **4b**.

In addition, methods have been used to reduce the nitro group, such as NaBH_4_/Cu(NO_3_)_2_ ×3H_2_O, SnCl_2_×2H_2_O [32], or NH_2_-NH_2_ by microwave irradiation [33]. 1-(3′-Nitro-, 4′-nitrophenyl)-6,7-dimethoxy-1,2,3,4-tetrahydroisoquinolines were reduced with SnCl_2_×2H_2_O and 1-(3′-amino-, 4′-aminophenyl)-6,7-dimethoxy-1,2,3,4-tetrahydroisoquinoline with yields of in 94% and 97% respectively were obtained as shown in Scheme 2. The spectral characteristics of compounds **3a**–**r**, **4a, b** confirm the formation of substituted 1-aryltetrahydroisoquinolines. This includes the presence of the active hydrogen band in the IR spectra and in ^1^H NMR spectra of aromatic protons singlets which are H-5 at δ_H_ 6.23–6.91 and H-8 at δ_H_ 6.09–6.80, as well as the singlet of the methine proton H-1 at δ_H_ 4.78–5.92. It should be noted that protons H-8 resonate in a stronger field than H-5, and the chemical shift of these protons depends slightly on the nature of the substituents in the C ring (only the presence of the nitro group causes a weak field shift of H-5 by ~0.5 ppm). Whereas substituents of the second type, as well as ortho-substituents, significantly affect the chemical shift of the H-1 proton.

### 2.1. In Silico ADMET/TOPKAT Prediction

**Figure 1:**
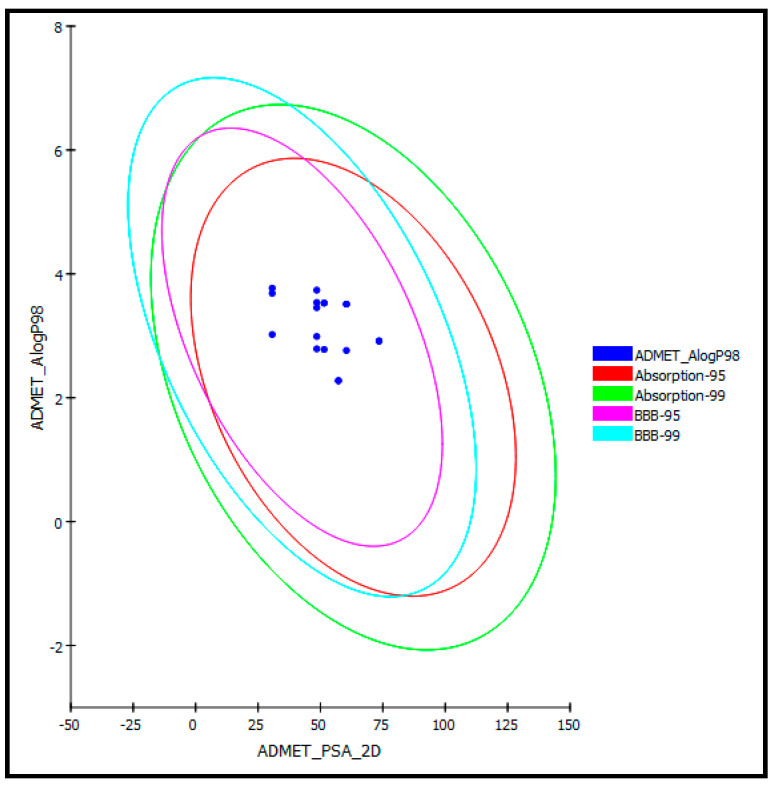
ADMET plot for a series of synthesized 1-aryltetrahydroisoquinoline derivatives displaying 95% and 99% confidence limit ellipses corresponding to the blood–brain barrier (BBB) and the human intestinal absorption (HIA) models.

ADMET/TOPKAT evaluation was performed in silico using Discovery Study 2016 (Accelrys Inc., San Diego, CA, USA) for the synthesized series of 1-aryltetrahydroisoquinoline derivatives. The ADMET evaluation results shown in Table 1 showed good human intestinal absorption level for all tested compounds, as thus they were within the 95% absorption ellipses as revealed by the ADMET graph (Figure 1). Furthermore, all 1-aryltetrahydroisoquinoline derivatives tested showed good to optimal levels of solubility. In addition, all 1-aryltetrahydroisoquinoline derivatives tested exhibited medium to high blood–brain barrier (BBB) penetration and therefore allocated within the 95% of confidence ellipse of the BBB (Figure 1). Meanwhile, all compounds revealed more than 90% plasma protein binding (PPB) pattern. Furthermore, they showed no inhibition of cytochrome P450 2D6 (CYP2D6) reflecting its inability to trigger uncontrolled drug–drug interactions but, unfortunately, showed hepatotoxicity with varying degrees (Table 1).

For TOPKAT prediction displayed in Table 2, all examined synthesized series of 1-aryltetrahydroisoquinoline derivatives revealed no mutagenicity with respect to the in silico Ames Mutagenicity evaluation. They also revealed no carcinogenicity in both rat male and female FDA. In addition, most of the compounds caused no irritation to the skin with the exception of **3i**, **3m**, and **3n** that showed mild skin irritancy. However, most of the compounds showed mild ocular irritation with the exception of **3c**, **3d**, **3e**, **3f**, **3l**, **3m**, and **3p** that triggered severe eye irritation. Meanwhile, the tested compounds showed rat chronic LOAEL (lowest observed adverse effect level) values ranging between 0.004 and 0.026 g/kg body weight. From ADMET/TOPKAT in silico evaluation, it was obvious that the examined synthesized compounds exhibited acceptable pharmacodynamic and pharmacokinetic properties but unfortunately caused hepatotoxicity with no carcinogenic or mutagenic potential.

### 2.2. Biological Evaluation

Acute toxicity studies have shown a similar pattern of resorptive action where the introduction of subtoxic and toxic doses resulted in depression of general state, suppression of motor activity, short-term respiration rate, head tremor, convulsions, and death of some animals in which the LD_50_ of the compounds tested was shown in Table 3.

The first compound,1-phenyl-6,7-dimethoxy-1,2,3,4-tetrahydroisoquinoline hydrochloride (**3a**), showed the maximum toxicity with LD_50_ of 280 mg/kg in contrast to 1-(3′-bromo-4′-hydroxyphenyl)-6,7-methylenedioxy-1,2,3,4-tetrahydroisoquinoline hydrochloride (**3e**) which was found to be the safest of the studied compounds. Its toxicity was 13.75 times lower than that of compound **3a**. However, introduction of the metylenedioxy group into the aryl radical at the C-3′ and C-4′ atoms of **3i** did not lead to a change in acute toxicity.

Conversely 3k, the additional introduction of a chlorine atom into the C-6′ 3k compound resulted in a 1.86-fold decrease of acute toxicity, and the introduction of a bromine atom (3j) at this position resulted in a decrease than 2.5 times the acute toxicity. Meanwhile, the presence of an oxy or methoxy group at the C-2 position (3b and 3p), a hydroxyl group at C-2, and a bromine atom at C-5 (3f) did not result in a significant decrease of toxicity. Simultaneous administration of bromine at C-2, methoxyl at C-4, and hydroxyl at C-5 (3h) reduced acute toxicity about 2-fold compared with 3a, while compound 3n with a nitro group at C-3 had showed an LD50 value of 460.5 ± 598.5 mg/kg. Introduction of methoxy groups into the phenyl radical at C-4 and C-5 positions with simultaneous bromination at the C-2 position (compound **3g**) resulted in no significant change in acute toxicity compared to **3a**. However, the introduction of methoxy groups into the phenyl radical at C-4 and C-5 (compound **3d**), bromination of the parent compound at C-3 (compound **3o**), as well as chlorination or nitration at C-4 (compounds **3m**, **3r**) reduced the acute toxicity (**3a**) by about 2.5–2.6 times, and the amino group in C-4 by 3.75 times (**4a**). Substitution of the nitro group with a dimethylamino radical reduced toxicity nearly 4.5-fold (compound **3q**). The presence of a hydroxy- at C-3 and a methoxy group at C-4 resulted in a 3.5-fold decrease of LD_50_ (compound **3c**). Simultaneous introduction of bromine atom at C-5′, hydroxyl at C-4, and methoxy group at C-3 into the initial molecule (**3a**) reduced the toxicity by 5.6-fold (compound **3l**), and the nitro group in C-4′—by nearly 7-fold (compound **3m**).

The local anesthetic activity of the studied compounds was evaluated on rabbit corneal model simulating surface anesthesia. During the experiments, no local irritant effects on rabbit eyes were observed in the concentrations studied, with the exception of compounds **3r**, **3n**, and **4a** which caused reversible irritation and redness of the eyes. As shown in Table 4, all studied derivatives (except **4b**) at a concentration 1% were more active than lidocaine, providing a longer duration of complete anesthesia. The compounds **3f**, **3h**, **3l**, **3n**, **3p**, and **3q** showed a duration of complete anesthesia for 1.6, 1.7, 1.4, 2.6, 3.0, and 2.2 times longer than lidocaine. The compounds **3c–f**, **3l**, **3o**, and **3p** revealed a longer total duration of anesthesia than lidocaine.

## 3. Materials and Methods

### 3.1. General Methods

Solvents and reagents were purchased from Sigma-Aldrich (Sigma-Aldrich GmbH, Steinheim, Germany). Starting materials or some benzaldehydes containing bromine and chlorine were synthesized corresponding to the benzaldehyde in the laboratory. The purity of the reagents was 95–98%. Mass spectra were recorded using an Agilent 1200 Infinity high performance liquid chromatograph (Agilent Technologies, Santa Clara, CA USA) and a 6420 Triple Quad LC-MS mass detector (Agilent Technologies, Santa Clara, CA USA USA) (electrospray ionization +ESI TIC Scan). IR spectra were recorded using the FTIR System 2000 spectrometer (PerkinElmer, Inc., Waltham, MA, USA,) in KBr tablets. NMR spectra were recorded on a JNM-ECX400 and JNM-ECZ600R spectrometer (Jeol, Akishima, Japan) at operating frequencies of 400 and 600 MHz; for ^1^H assessment the solvents were CDCl_3_, CCl_4_, CD_3_COOD, and DMSO-d_6_. Meanwhile, TMS (0 ppm) was used as an internal standard in NMR spectra. In the ^13^C NMR spectra, a chemical solvent shift (CDCl_3_, 77.16 ppm relative to TMS) was used as an internal standard. The software MestReNova 14.2.0 (Mestrelab Research S.L., Santiago de Compostela, Spain) processed NMR spectra. The reaction progress and the purity of the obtained compounds were monitored by TLC on Silufol L/W 10 × 20 cm plates (Sigma-Aldrich GmbH, Steinheim, Germany) with a fluorescence indicator at 254 nm (Germany) in different solvent systems CHCl_3_:MeOH, 6:1 (System I) and 8:1 (System II). The melting point temperatures (m.p.) of all synthesized substances were determined on a BOETIUS microheating plate (Veb Analytik, Dresden, Germany).

### 3.2. Synthesis of 1-Aryl-6,7-dimethoxy-1,2,3,4-tetrahydroisoquinoline Derivatives ***3 a–r***

General Methodology

A mixture of 3,4-dimethoxyphenylethylamine (**1**) (0.015 mol) with **2 a–r** substituted benzaldehyde (0.015 mol) in 10–20 mL trifluoroacetic acid was boiled under reflux for 2–4 h. The progress of the reaction was monitored using TLC and then the reaction mixture was cooled with ice followed by its alkalinization with 10% NaOH aqueous solution to reach pH 9–10. The amine was exhaustively extracted with chloroform. After the distillation of chloroform, the crystallization of the crude product was carried out by an appropriate solvent or by dissolving in acetone and acidified with concentrated hydrochloric acid to reach pH 5–6. The precipitated hydrochloride was filtered, washed three times with acetone and recrystallized from ethyl alcohol. The obtained hydrochloride salt was dissolved in H_2_O (20 mL) and aqueous NaOH (10%) was added to adjust the pH to 8–9. The mixture was extracted with CHCl_3_ (60 mL × 4). The combined organic layer was dried over anhydrous Na_2_SO_4_ and evaporated in vacuo to give free amine.

#### 3.2.1. 1-(Phenyl)-6,7-dimethoxy-1,2,3,4-tetrahydroisoquinoline (**3a**), C_17_H_19_O_2_N

Obtained from 2.76 g (15.25 mmol) of 3,4-dimethoxyphenylethylamine (**1**) and 1.62 g (15.25 mmol) of benzaldehyde (**2a**). Yield 3.85 g (94%), m.p. 118–121°C (acetone); R*_f_* 0.51 (System I). ^1^H NMR (600 MHz, CDCl_3_, δ, ppm, J/Hz): 2.73 (1H, *dt*, *J* = 4.8, 15.9, H_a_-4), 2.91 (1H, *m*, H_b_-4), 3.02 (1H, *ddd*, *J* = 4.7, 8.3, 12.2, H_a_-3), 3.19 (1H, *dt*, *J* = 5.3, 12.2, H_b_-3), 3.61 (3H, *s*, 7-OCH_3_), 3.85 (3H, *s*, 6-OCH_3_), 5.02 (1H, *s*, H-1), 6.23 (1H, *s*, H-8), 6.62 (1H, *s*, H-5), 7.23–7.30 (5H, *m*, Ar-H); ^13^C NMR (δ, ppm): 29.44 (C-4), 42.00 (C-3), 55.93 (6,7-OCH_3_), 61.58 (C-1), 111.06 (C-8), 111.53 (C-5), 113. 49 (C-3′), 115.04 (C-5′), 127.43 (C-4′), 127.78 (C-4a), 128.48 (C-3′,5′), 128.99 (C-2′,6′), 130.02 (C-8a), 145.03 (C-1′), 147.14 (C-7), 147.70 (C-6)

#### 3.2.2. 1-(2′-Hydroxyphenyl)-6,7-dimethoxy-1,2,3,4-tetrahydroisoquinoline (**3b**), C_17_H_18_O_3_N

Obtained from 2.12 g (11.71 mmol) of 3,4-dimethoxyphenylethylamine (**1**) and 1.43 g (11.71 mmol) of 2-hydroxybenzaldehyde (**2b**). Yield 3.04 g (91%); m.p. 159–162 °C (acetone); R*_f_* 0.62 (System I). IR (KBr, ν_max_, cm^–1^): 3310, 30002, 2954, 2936, 2864, 2830, 2698, 2601, 1711, 1600, 1581, 1516, 1563, 1450, 1361, 1299, 1258, 1220, 1167, 1147, 1120, 1095, 1070, 1036, 1015, 944, 860, 845.^1^H NMR (400 MHz, CDCl_3_, δ, ppm, J/Hz): 2.74 (1H, *dt*, *J* = 4.7, 16.2, H_a_-4), 2.94–3.01 (1H, *m*, H_a_-3), 3.08 (1H, *ddd*, *J* = 4.6, 8.5, 11.9, H_b_-4), 3.25 (1H, *dt*, *J* = 5.2, 11.9, H_b_-3), 3.66 (3H, *s*, 7-OCH_3_), 3.84 (3H, *s*, 6-OCH_3_), 5.11 (1H, *s*, H-1), 6.37 (1H, *s*, H-8), 6.61 (1H, *s*, H-5), 6.79–6.84 (2H, *m*, H-3, 4), 7.02 (1H, *dd,J* = 1.5, 7.5, H-6′), 6.19 (1H, *ddd*, *J* = 1.7, 7.3, 8.2, H-5′). ^13^C NMR (δ, ppm): 24.69 (C-4), 37.94 (C-3), 52.05 (6-OCH_3_), 52.10 (7-OCH_3_), 56.32 (C-1), 106.67 (C-8), 107.79 (C-5), 113. 49 (C-3′), 115.04 (C-5′), 122.49 (C-4a), 123.08 (C-8a), 123.73 (C-1′), 125.36 (C-6′), 125.63 (C-4′), 143.45 (C-7), 144.26 (C-6), 153.54 (C-2′).

#### 3.2.3. 1-(3′-Hydroxy-4′-methoxyphenyl)-6,7-dimethoxy-1,2,3,4-tetrahydroisoquinoline (**3c**), C_18_H_21_O_4_N

Obtained from 2.82 g (15.58 mmol) of 3,4-dimethoxyphenylethylamine (**1**) and 1.52 g (15.58 mol) of isovanilline (**2c**). Yield 4.36 g (89%); m.p. of hydrochloride 199–203 °C (acetone); R*_f_* 0.67 (System I). IR (KBr, ν_max_, cm^–1^): 3431, 3290, 2929, 2848, 1596, 1514, 1455, 1434, 1361, 1299, 1271, 1258, 1239 1225, 1162, 1136, 1096, 1016, 849, 810. ^1^H NMR (400 MHz, DMSO-d_6_, δ, ppm, J/Hz): 2.92 (1H, *dt*, *J* = 4.8, 16.6, H_a_-4), 3.05–3.11 (1H, *m*, H_b_-4), 3.19 (2H, *q*, *J* = 5.0, 5.9, H-3), 3.46 (3H, *s*, 7-OCH_3_), 3.70 (3H, *s*, 6-OCH_3_), 3. 71 (3H, *s*, 4′-OCH_3_), 5.43 (1H, *s*, H-1), 6.23 (1H, *s,* H-8), 6.70–6.73 (2H, *m*, H-2′, H-6′), 6.80 (1H, *s*, H-5), 6.90 (1H, *d*, *J* = 8.8, H-5′). ^13^C NMR (δ, ppm): 24.56 (C-4), 38.92 (C-3), 55.86 (6, 7-OCH_3_) 55.91 (4′-OCH_3_), 57.75 (C-1), 111.42 (C-8), 112.07 (C-5), 112. 52 (C-5′), 117.74 (C-2′), 121.91 (C-6′), 124.53 (C-8a), 125.64 (C-4a), 130.04 (C-1′), 147.24 (C-3′), 148.24 (C-6), 149.14 (C-7), 149.31 (C-4′).

#### 3.2.4. 1-(3′,4′-Dimethoxyphenyl)-6,7-dimethoxy-1,2,3,4-tetrahydroisoquinoline (**3d**), C_19_H_23_O_4_N

Obtained from 3.16 g (17.46 mmol) of 3,4-dimethoxyphenylethylamine (**1**) and 2.89 g (17.46 mmol) of veratrum aldehyde (**2d**). Yield 5.28 g (92%); m.p. 72–74 °C (acetone); R*_f_* 0.66 (System I). Mass- (+ESI) (*m*/*z*, *I*_rel_, %): 330 [M + H]^+^, 282 (10), 267 (17), 161 (73), 151 (100), 155 (42). IR (KBr, ν_max_, cm^−1^): 3568, 3276, 2922, 2823, 1646, 1609, 1517, 1463, 1254, 1217, 1136, 1115, 1020, 999, 870. ^1^H NMR (400 MHz, CDCl_3_, δ, ppm, *J*/Hz): 2.73 (1H, *dt*, *J* = 4.4, 16.1, H_a_-4), 2.95 (1H, *ddd*, *J* = 5.5, 8.4, 14.4, H_a_-3), 3.05 (1H, *ddd*, *J* = 4.5, 8.7, 12.1, H_b_-4), 3.24 (1H, *dt*, *J* = 4.9, 11.6, H_b_-3), 3.65 (3H, *s*, 7-OCH_3_), 3.83 (3H, *s*, 6-OCH_3_), 3.87 (3H, *s*, 3′-OCH_3_), 3.88 (3H, *s*, 4′-OCH_3_), 4.99 (1H, *s*, H-1), 6.27 (1H, *s*, H-8), 6.63 (1H, *s*, H-5), 6.78 (1H, *dd*, *J* = 1.5, 8.1, H-6′), 6.80 (1H, *d*, *J* = 1.5, H-2′), 6.81 (1H, *d*, *J* = 8.1, H-5′). ^13^C NMR (δ, ppm): 29.26 (C-4), 42.25 (C-3), 55.86 (6-OCH_3_), 55.91 (7-OCH_3_), 55.91 (3′-OCH_3_), 55.91 (4′-OCH_3_), 61.47 (C-1), 110.69 (C-8), 110. 89 (C-5), 111.39 (C-5′), 111.80 (C-2′), 121.28 (C-6′), 127.58 (C-8a), 130.08 (C-4a), 137.33 (C-1′), 147.01 (C-6), 147.61 (C-7), 148.34 (C-3′), 148.99 (C-4′).

#### 3.2.5. 1-(3′-Bromo-4′-hydroxyphenyl)-6,7-dimethoxy-1,2,3,4-tetrahydroisoquinoline (**3e**), C_17_H_18_O_3_NBr

Obtained from 2.18 g (12.04 mmol) of 3,4-dimethoxyphenylethylamine (**1**) and 2.42 g (12.04 mmol) of 3-bromo-4-hydroxybenzaldehyde (**2e**). Yield 3.86 g (88%); m.p. of hydrochloride 205–207 °C (acetone); R*_f_* 0.69 (System II). ^1^H NMR (600 MHz, DMSO-*d*_6_, δ, ppm, J/Hz): 2.64 (1H, *dt*, *J* = 4.8, 15.8, H_a_-4), 2.80–2.85 (1H, *m*, H_a_-3), 2.88–2.92 (2H, m, H_b_-4) 2.64 (1H, *dt*, *J* = 5.1, 10.4, H_b_-3), 3.54 (3H, *s*, 7-OCH_3_), 3.73 (3H, *s*, 6-OCH_3_), 4.86 (1H, *s*, H-1), 6.14 (1H, *s*, H-8), 6.55 (1H, *s*, H-5), 6.82 (1H, *d*, *J* = 8.3, H-5′), 6.92 (1H, *dd*, *J* = 2.2, 8.3, H-6′), 7.27 (1H, *d*, *J* = 2.2, H-2′). ^13^C NMR (δ, ppm): 28.87 (C-4), 41.55 (C-3), 55.81 (7-OCH_3_), 56.01 (6-OCH_3_), 59.98 (C-1), 109.62 (C-3′), 111.99 (C-8), 112. 48 (C-5), 116.19 (C-5′), 127.67 (C-4a), 129.33 (C-6′), 129.75 (C-8a), 133.52 (C-2′), 136.56 (C-1′), 147.50 (C-6), 148.19 (C-7), 153.85 (C-4′).

#### 3.2.6. 1-(5′-Bromo-2′-hydroxyphenyl)-6,7-dimethoxy-1,2,3,4-tetrahydroisoquinoline (**3f**), C_17_H_18_O_3_NBr

Obtained from 2.28 g (12.59 mmol) of 3,4-dimethoxyphenylethylamine (**1**) and 2.53 g (12.59 mmol) of 5-bromo-2-oxybenzaldehyde (**2f**). Yield 4.41 g (96%); m.p. of hydrochloride 189–192 °C (acetone); R*_f_* 0.51 (System II). IR (KBr, ν_max_, cm^−1^): 3304, 2932, 2832, 1609, 1579, 1515, 1478, 1258, 1220, 1169, 1119, 1030, 972, 817. ^1^H NMR (400 MHz, CDCl_3_, δ, ppm, J/Hz): 2.76 (1H, *dt*, *J* = 5.5, 16.5, H_a_-4), 2.92 (1H, *dt*, *J* = 5.9, 16.5, H_a_-3), 3.09 (1H, *ddd*, *J* = 5.2, 7.3, 12.5, H_b_-4), 3.25 (1H, *dt*, *J* = 5.9, 12.1, H_b_-3), 3.72 (3H, *s*, 7-OCH_3_), 3. 85 (3H, *s*, 6-OCH_3_), 5.12 (1H, *s*, H-1), 6.37 (1H, *s*, H-8), 6.61 (1H, *s*, H-5), 6.71 (2H, d, *J* = 8.6, H-3′), 7.05 (1H, d, *J* = 2.0, H-6′), 7.26 (1H, *dd*, *J* = 2.0, 8.6, H-4′). ^13^C NMR (δ, ppm): 28.27 (C-4), 41.28 (C-3), 56.27 (6-OCH_3_), 56.42 (7-OCH_3_), 59.43 (C-1), 111.21 (C-8), 111.41 (C-4′), 112. 46 (C-5), 119.78 (C-3′), 126.91 (C-4a), 127.03 (C-8a), 129.44 (C-1′), 132.71 (C-6′), 132.76 (C-5′), 148.33 (C-7), 149.32 (C-6), 157.50 (C-2′).

#### 3.2.7. 1-(2′-Bromo-4′,5′-dimethoxyphenyl)-6,7-dimethoxy-1,2,3,4-tetrahydroisoquinoline (**3g**), C_19_H_22_O_4_NBr

Obtained from 2.56 g (14.14 mmol) of 3,4-dimethoxyphenylethylamine (**1**) and 3.53 g (14.41 mmol) of 2-bromo-4,5-dimethoxybenzaldehyde (**2g**). Yield 5.35 g (93%), m.p. 144–145 °C (acetone); R*_f_* 0.50 (System II). IR (KBr, ν_max_, cm^−1^): 3332, 2994, 2950, 2904, 2839, 1598, 1518, 1505, 1459, 1440, 1379, 1329, 1232, 1202, 1187, 1154, 1111, 1025, 1041, 985, 943, 879, 841, 807. ^1^H NMR (400 MHz, CDCl_3_, δ, ppm, J/Hz): 2.77 (1H, *dt*, *J* = 4.6, 15.8, H_a_-4), 2.95 (1H, (1H, *ddd*, *J* = 5.2, 8.1, 13.8, H_a_-3), 3.07 (1H, *ddd*, *J* = 4.6, 8.1, 11.9, H_b_-4), 3.19 (1H, *dt*, *J* = 5.2, 11.6, H_b_-3), 3.67 (3H, *s*, 7-OCH_3_), 3. 68 (3H, *s*, 6-OCH_3_), 3.88 (3H, *s*, 3′-OCH_3_), 3.89 (3H, *s*, 4′-OCH_3_), 5.43 (1H, *s*, H-1), 6.26 (1H, *s*, H-8), 6.57 (1H, *s*, H-5), 6.64 (1H, *s*, H-6′), 7.06 (1H, *s*, H-3′). ^13^C NMR (δ, ppm): 29.34 (C-4), 41.98 (C-3), 55.83 (6-OCH_3_), 55.96 (7-OCH_3_), 56.03 (3′-OCH_3_), 56.18 (4′-OCH_3_), 59.90 (C-1), 110.67 (C-8), 111. 43 (C-5), 113.29 (C-6′), 114.54 (C-2′), 115.13 (C-3′), 127.91 (C-4a), 129.02 (C-8a), 135.73 (C-1′), 147.09 (C-6), 147.66 (C-7), 148.29 (C-4′), 148.78 (C-5′).

#### 3.2.8. 1-(2′-Bromo-5′-hydroxy-4′-methoxyphenyl)-6,7-dimethoxy-1,2,3,4-tetrahydroisoquinoline (**3h**), C_18_H_20_O_4_NBr

Obtained from 2.06 g (11.38 mmol) of 3,4-dimethoxyphenylethylamine (**1**) and 2.63 g (11.38 mol) of 2-bromo-5-hydroxy-4-methoxybenzaldehyde (**2h**). Yield 3.85 g (85%); m.p 199–200 °C (acetone); R*_f_* 0.57 (System II). Mass (+ESI) (*m*/*z*, *I*_rel_,%): 394/396 [M + H]^+^ with bromine isotopes 79 and 81.^1^H NMR (400 MHz, CDCl_3_, δ, ppm, J/Hz): 2.74 (1H, *dt*, *J* =5.4, 15.9, Ha-4), 2.86 (1H, *dt*, *J* =5.5, 15.7, H_b_-4), 2.97 (1H, *ddd*, *J* = 4.9, 7.2, 11.9, H_a_-3), 3.13 (1H, *ddd*, *J* = 4.9, 6.3, 11.4, H_b_-3), 3.66 (3H, *s*, 7-OCH_3_), 3.82 (3H, *s*, 6-OCH_3_), 3.84 (3H, *s*, 4′-OCH_3_), 5.36 (1H, *s*, H-1), 6.23 (1H, *s*, H-8), 6.45 (1H, *s*, H-3′), 6.59 (1H, *s*, H-5), 6.94 (1H, *s*, H-6′). ^13^C NMR (δ, ppm): 29.11 (C-4), 40.91 (C-3), 55.95 (6-OCH_3_), 55.98 (7-OCH_3_), 56.24 (5′-OCH_3_), 59.25 (C-1), 110.84 (C-8), 111.48 (C-5), 113. 09 (C-2′), 114.84 (C-6′), 118.22 (C-3′), 127.94 (C-4a), 128.36 (C-8a), 135.88 (C-1′), 145.07 (C-4′), 147.10 (C-6), 147.35 (C-7), 147.87 (C-5′).

#### 3.2.9. 1-(3′,4′-Methylenedioxyphenyl)-6,7-dimethoxy-1,2,3,4-tetrahydroisoquinoline (**3i**), C_18_H_19_O_4_N

Obtained from 2.66 g (14.69 mmol) of 3,4-dimethoxyphenylethylamine (**1**) and 2.21 g (14.73 mmol) of 3,4-methylenedioxybenzaldehyde (**2i**). Yield 4.10 (89%); m.p. of hydrochlorohyde 254–257 °C (acetone); R*_f_* 0.52 (System I). IR (KBr, ν_max_, cm^−1^): 3250, 2908, 2815, 1608, 1518, 1487, 1444, 1370, 1250, 1215, 1123, 1107, 1042, 967, 942, 926, 871, 834, 761, 710. ^1^H NMR (400 MHz, CDCl_3_, δ, ppm, J/Hz): 2.66 (1H, *dt*, *J* = 4.6, 15.9, H_a_-4), 2.85 (1H, *ddd*, *J* = 5.3, 8.3, 13.8, H_b_-4), 2.93–2.99 (1H, *m*, H_a_-3), 3.15 (1H, *dt*, *J* = 5.2, 12.1, H_b_-3), 3.61 (3H, *s*, 7-OCH_3_), 3.80 (3H, *s*, 6-OCH_3_), 4.90 (1H, *s*, H-1), 5. 87 (2H, *s*, 3′-OCH_2_O-4′), 6.20 (1H, *s*, H-8), 6.54 (1H, *s*, H-5), 6.64 (1H, *dd*, *J* = 0.6, 1.5, H-2′), 6.67 (1H, *dd*, *J* = 1.5, 7.9, H-6′), 2.69 (1H, *dd*, *J* = 0.7, 7.9, H-5′). ^13^C NMR (δ, ppm): 29.51 (C-4), 42.09 (C-3), 56.06 (6-OCH_3_), 56.12 (7-OCH_3_), 61.42 (C-1), 101.16 (C-7′), 108.08 (C-5′), 109. 34 (C-2′), 111.17 (C-8), 111.64 (C-5), 122.39 (C-6′), 127.84 (C-1′), 130.14 (C-8a), 139.24 (C-4a), 146.99 (C-6), 147.29 (C-7), 147.86 (C-3′), 147.91 (C-4′).

#### 3.2.10. 1-(2′-Bromo-4′,5′-methylenedioxyphenyl)-6,7-dimethoxy-1,2,3,4-tetrahydroisoquinoline (**3j**), C_18_H_18_O_4_NBr

Obtained from 4.39 g (24.25 mmol) of 3,4-dimethoxyphenylethylamine (**1**) and 5.55 g (24.25 mmol) of 2-bromo-3,4-methylenedioxybenzaldehyde (**2j**). Yield 8.35 g (94%); m.p. 201–203 °C (acetone); R*_f_* 0.52 (system II). IR (KBr, ν_max_, cm^−1^): 3424, 2967, 2840, 2562, 1674, 1516, 1479, 1445, 1393, 1374, 1306, 1285, 1215, 1129, 1034, 998, 927, 861, 841, 801, 762, 719. ^1^H NMR (400 MHz, CDCl_3_, δ, ppm, J/Hz): 2.79 (1H, *dt*, *J* = 5.6, 16.2, H_a_-4), 2.84–2.91 (1H, *m*, H_b_-4), 2.99–3.05 (2H, *m*, H-3), 3.11 (1H, *ddd*, *J* = 5.0, 6.6, 11.8, H_b_-3), 3.71 (3H, *s*, 7-OCH_3_), 3. 88 (3H, *s*, 6-OCH_3_), 5.44 (1H, *s*, H-1), 5.93 (2H, *dd*, *J* = 1.3, 7.0, 4′-OCH_2_O-5′), 6.27 (1H, *s*, H-8), 6.45 (1H, *s*, H-6′), 6.63 (1H, *s*, H-5), 7.04 (1H, *s*, H-3′). ^13^C NMR (δ, ppm): 29.27 (C-4), 40.48 (C-3), 55.90 (6-OCH_3_), 55.99 (7-OCH_3_), 59.42 (C-1), 101.80 (C-7′), 110.74 (C-5′), 110.76 (C-2′), 111.46 (C-8), 112.52 (C-5), 114.78 (C-6′), 128.00 (C-1′), 128.61 (C-8a), 137.19 (C-4a), 147.24 (C-6), 147.29 (C-7), 147.53 (C-3′), 147.83 (C-4′).

#### 3.2.11. 1-(4′,5′-Methylenedioxy-2′-chlorophenyl)-6,7-dimethoxy-1,2,3,4-tetrahydroisoquinoline (**3k**), C_18_H_18_O_4_NCl

Obtained from 4.20 g (23.20 mmol) of 3,4-dimethoxyphenylethylamine (**1**) and 4.29 g (23.20 mmol) of 2-chloro-4,5-methylenedioxybenzaldehyde (**2k**). Yield 6.94 g (86%); m.p 107–108°C (acetone), R*_f_* 0.73 (System II). ^1^H NMR (400 MHz, CDCl_3_,δ, ppm, J/Hz): 2.80 (1H, *dt*, *J* = 5.9, 16.4, H_a_-4), 2.87 (1H, *dt*, *J* =5.5, 16.1, H_b_-4), 3.01–3.12 (2H, *m*, H-3), 3.71 (3H, *s*, 7-OCH_3_), 3. 88 (3H, *s*, 6-OCH_3_), 5.48 (1H, *s*, H-1), 5.92 (2H, *dd*, *J* = 1.6, 6.0, 4′-OCH_2_O-5′), 6.27 (1H, *s*, H-8), 6.44 (1H, *s*, H-5), 6.63 (1H, *s*, H-3′), 6.88 (1H, *s*, H-6′).

#### 3.2.12. 1-(5′-Bromo-4′-hydroxy-3′-methoxyphenyl)-6,7-dimethoxy-1,2,3,4-tetrahydroisoquinoline (**3l**), C_18_H_20_O_4_NBr

Obtained from 2.51 g (13.87 mmol) of 3,4-dimethoxyphenylethylamine (**1**) and 3.21 g (13.89 mmol) of 5-bromovaniline (**2l**). Yield 4.91 (90%); m.p. 193–201 °C (acetone); R*_f_* 0.74 (System II). IR (KBr, ν_max_, cm^−1^): 3288, 2974, 2936, 2848, 1607, 1577, 1515, 1441, 1425, 1355, 1326, 1283, 1256, 1150, 1107, 1050, 997, 869, 807. ^1^H NMR (400 MHz, DMSO-*d*_6_,δ, ppm, J/Hz): 2.57–2.65 (1H, *m*, H_a_-4), 2.78–2.90 (2H, *m*, H-3,4), 3.06 (1H, *dd*, J= 5.9, 11.1, H_b_-3), 3.55 (3H, *s*, 7-OCH_3_), 3.73 (3H, *s*, 6-OCH_3_), 3.76 (3H, *s*, 3′-OCH_3_), 4.78 (1H, *s*, H-1), 6.17 (1H, *s*, H-8), 6.54 (1H, *s*, H-5), 6.73 (1H, *s,* H-2′), 7.04 (1H, *s*, H-6′). ^13^C NMR (δ, ppm): 29.18 (C-4), 41.87 (C-3), 55.74 (6-OCH_3_), 56.05 (7-OCH_3_), 56.42 (C-3′), 60.64 (C-1), 96.23 (C-2′, 5′, 6′), 109.11 (C-1′), 111.96 (C-8), 112.46 (C-5), 125.15 (C-8a), 127.81 (C-4a), 143.66 (C-6), 147.39 (C-7), 148.15 (C-3′), 148.49 (C-4′).

#### 3.2.13. 1-(4′-Nitrophenyl)-6,7-dimethoxy-1,2,3,4-tetrahydroisoquinoline (**3m**), C_17_H_18_O_4_N_2_

Obtained from 2.50 g (13.81 mmol) of 3,4-dimethoxyphenylethylamine (**1**) and 2.10 g (13.81 mmol) of 4-nitrobenzaldehyde (**2m**). Yield 4.17 g (96%); m.p. 139–140 °C (acetone); R_f_ 0.63 (System II). IR (KBr, ν_max_, cm^−1^): 3244, 2965, 2832, 1607, 1515, 1349, 1257, 1221, 1113. ^1^H NMR (400 MHz, CDCl_3_, δ, ppm, J/Hz): 2.70 (1H, *dt*, *J* = 5.0, 16.1, H_a_-4), 2.83–2.90 (1H, *m*, H_b_-3), 2.96–3.03 (1H, *m*, H_b_-4), 3.07–3.13 (1H, *m*, H_a_-3), 3.58 (3H, *s*, 7-OCH_3_), 3. 81 (3H, *s*, 6-OCH_3_), 5.08 (1H, *s*, H-1), 6.09 (1H, *s*, H-8), 6.59 (1H, *s*, H-5), 7.38 (2H, *d*, *J* = 8.5, H-2′,6′), 8.11 (2H, *d*, *J* = 8.5, H-3′,5′).

#### 3.2.14. 1-(3′-Nitrophenyl)-6,7-dimethoxy-1,2,3,4-tetrahydroisoquinoline (**3n**), C_17_H_18_O_4_N_2_

Obtained from 3.21 g (17.73 mmol) of 3,4-dimethoxyphenylethylamine (**1**) and 2.68 g (17.73 mmol) of 3-nitrobenzaldehyde (**2n**). Yield 5.34 g (95%); m.p. 216–218 °C (acetone); R*_f_* 0.56 (System II). IR (KBr, ν_max_, cm^−1^): 3442, 3007, 2840, 1680, 1531, 1354, 1264, 1240, 1136. ^1^H NMR (400 MHz, DMSO-d_6_, δ, ppm, J/Hz): 3.00 (1H, *dt*, *J* = 6.3, 17.2, H_a_-4), 3.12 (1H, *dt*, *J* = 6.0, 17.2, H_b_-3), 3.27–3.35 (2H, *m,* H_a_-3, H_b_-4), 3.51 (3H, *s*, 7-OCH_3_), 3.78 (3H, *s*, 6-OCH_3_), 5. 92 (1H, *s*, H-1), 6.31 (1H, *s*, H-8), 6.91 (1H, *s*, H-5), 7.71 (1H, *d*, *J* = 7.7, H-6′), 7.75 (1H, *t*, *J* = 7.7, H-5′), 8.25 (1H, *s*, H-2′), 8.31 (1H, *d*, *J* = 7.7, H-4′).

#### 3.2.15. 1-(3′-Bromophenyl)-6,7-dimethoxy-1,2,3,4-tetrahydroisoquinoline (**3o**), C_17_H_18_O_2_NBr

Obtained from 2.55 g (14.09 mmol) of 3,4-dimethoxyphenylethylamine (**1**) and 2.61 g (14.09 mmol) of 3-bromobenzaldehyde (**2o**). Yield 4.75 g (97%); m.p. of hydrochlorohyde 248–251°C (acetone); R*_f_* 0.78 (System II). IR (KBr, ν_max_, cm^−1^): 3244, 3235, 2994, 2960, 2922, 2892, 2826, 2722, 1610, 1590, 1508, 1456, 1331, 1253, 1214, 1177, 1114, 1043, 1018, 969, 852, 818. ^1^H NMR (400 MHz, CDCl_3_, δ, ppm, J/Hz): 1.84 (1H, *s*, NH), 2.72 (1H, *dt*, *J* = 4.9, 15.9, H_a_-4), 2.87–2.94 (1H, *m*, H_a_-3), 3.01 (1H, *ddd*, *J* = 4.7, 8.0, 12.3, H_b_-4), 3.16 (1H, *dd*, *J* = 5.3, 11.9, H_b_-3), 3.64 (3H, *s*, 7-OCH_3_), 3.86 (3H, *s*, 6-OCH_3_), 4.99 (1H, *s*, H-1), 6.21 (1H, *s*, H-8), 6.62 (1H, *s*, H-5), 7.16–7.18 (2H, *m*, Ar-H), 7.38–7.40 (2H, *m,* Ar-H). ^13^C NMR (δ, ppm): 29.30 (C-4), 41.77 (C-3), 55.94 (6-OCH_3_), 56.02 (7-OCH_3_), 61.02 (C-1), 110.88 (C-8), 111.61 (C-5), 122. 64 (C-8a), 127.69 (C-6′), 127.82 (C-4a), 129.04 (C-1′), 130.03 (C-5′), 130.60 (C-2′), 132.00 (C-4′), 147.24 (C-6), 147.43 (C-7), 147.90 (C-3′).

#### 3.2.16. 1-(2′-Methoxyphenyl)-6,7-dimethoxy-1,2,3,4-tetrahydroisoquinoline (**3p**), C_17_H_18_O_4_N_2_

Obtained from 2.67 g (14.75 mmol) of 3,4-dimethoxyphenylethylamine (**1**) and 2.01 g (14.75 mmol) of 2-methoxybenzaldehyde (**2p**). Yield 3.92 g (89%); m.p. of hydrochlorohyde 259–262 °C (acetone); R*_f_* 0.45 (System II). IR (KBr, ν_max_, cm^−1^): 3159, 2937, 2891, 1605, 1585, 1516, 1258, 1116. ^1^H NMR (400 MHz, CDCl_3_, δ, ppm, J/Hz): 3.00 (1H, *dt*, *J* = 5.5, 16.4, H_a_-4), 3.03–3.08 (1H, *m*, H_b_-4), 3.10–3.14 (2H, *m*, H-3), 3.59 (3H, *s*, 7-OCH_3_) 3.81 (3H, *s*, 6-OCH_3_), 3.82 (3H, *s*, 2′-OCH_3_), 5.75 (1H, *s*, H-1), 6.20 (1H, *s*, H-8), 6. 57 (1H, *s*, H-5), 6.82 (1H, *t*, *J* =7.5, H-5′), 6.87 (1H, *d*, *J* = 7.9, H-6′), 6.89 (1H, *dd*, *J* = 1.8, 8.0 H-3′), 7.29 (1H, *td*, *J* = 1.8, 7.5, H-4′), 8.83 and 10.73 (0.5 H each, *br. s,* NH).

#### 3.2.17. 1-(4′-Dimethylaminophenyl)-6,7-dimethoxy-1,2,3,4-tetrahydroisoquinoline (**3q**), C_19_H_24_O_2_N_2_

Obtained from 26.3 g (145.30 mmol) of 3,4-dimethoxyphenylethylamine (**1**) and with 21.67 g (145.30 mol) of 4-dimethylaminobenzaldehyde (**2q**). Yield 49.04 g (92%); m.p. of dihydrochlorohyde 246–248 °C, (acetone); R*_f_* 0.72 (System I). IR (KBr, ν_max_, cm^−1^): 3418, 2912, 2761, 2622, 2542, 1616, 1518, 1462, 1445, 1354, 1299, 1260, 1232, 1168, 1117, 1056, 1018, 985, 944, 876, 856, 818. ^1^H NMR (400 MHz, CDCl_3_, δ, ppm, J/Hz): 2.88 (6H, *s*, 4′-N(CH_3_)_2_), 2.80–2.84 (1H, *m*, H_a_-4), 2.97–3.02 (1H, *m*, H_b_-4), 3.06–3.14 (2H, *m*, H-3), 3.62 (3H, *s*, 7-OCH_3_), 3. 85 (3H, *s*, 6-OCH_3_), 5.31 (1H, *s*, H-1), 6.22 (1H, *s*, H-8), 6.58 (1H, *s*, H-5), 6.62 (2H, *d*, *J* = 9.0, H-2′,6′), 7.10 (2H, *d*, *J* = 9.0, H-3′,5′). ^13^C NMR (δ, ppm): 25.20 (C-4), 39.48 (C-3), 40.34 (4-N(CH_2_)_3_) 56.00 (6-OCH_3_), 56.04 (7-OCH_3_), 59.09 (C-1), 110.76 (C-8), 110.97 (C-5), 112.20 (C-3′, 5′), 123.59 (C-4a), 123.96 (C-8a), 124.76 (C-1′), 130.98 (C-2′, 6′), 148.08 (C-7), 148.91(C-6), 151.03 (C-4′).

#### 3.2.18. 1-(4′-Chlorophenyl)-6,7-dimethoxy-1,2,3,4-tetrahydroisoquinoline (**3r**), C_19_H_24_O_2_N_2_

Obtained from 1.51 g (8.34 mmol) of 3,4-dimethoxyphenylethylamine (**1**) and 1.24 g (8.34 mmol) of 4-chlorobenzaldehyde (**2r**). Yield 1.82 g (64%); m.p. of hydrochlorohyde 252–254 °C (acetone); R*_f_* 0.54 (System II). IR (KBr, ν_max_, cm^−1^): 3437, 2929, 2911, 1610, 1517, 1495, 1471, 1243, 1223, 1130, 1101, 1027, 955, 859. ^1^H NMR (400 MHz, CDCl_3_, δ, ppm, J/Hz): 3.02 (1H, *dt*, *J* = 6.0, 17.3 H_a_-4), 3.14 (1H, *dt*, *J* = 6.3, 17.3 H_b_-4), 3.26–3.39 (2H, *m*, H-3), 3.60 (3H, *s*, 7-OCH_3_), 3. 83 (3H, *s*, 6-OCH_3_), 5.51 (1H, *s*, H-1), 6.23 (1H, *s*, H-8), 6.73 (1H, *s*, H-5), 7.27 (2H, *d*, *J* = 8.5, H-2′,6′), 7.38 (2H, *d*, *J* = 8.5, H-3′,5′).

### 3.3. Reduction of 1-(Nitrophenyl)-6,7-dimethoxy-1,2,3,4-tetrahydroisoquinolines

General Methodology

9.0 mL of HCl (33%) were added to 6.70 g of SnCl_2_ ×2H_2_O followed by stirring of the mixture for 10–15 min on a magnetic stirrer at room temperature. Then a suspension consisting of 3.10 g of 1-(3′-nitro or 4′-nitrophenyl)-6,7-dimethoxy-1,2,3,4-tetrahydroisoquinoline in 20 mL of ethyl alcohol and 4.60 mL of HCl (33%) was added in portions. The reaction mixture was stirred for 30 min and boiled under reflux for 3 h. The course of reaction was monitored by TLC. Consequently, it was diluted with water and alkalized with 30% NaOH solution to strongly alkaline condition to reach pH 10. The products were extracted with CHCl_3_ (5 × 60 mL), the extracts were combined, and the solvent was removed by recrystallization from methanol.

#### 3.3.1. 1-(4′-Aminophenyl)-6,7-dimethoxy-1,2,3,4-tetrahydroisoquinoline (**4a**), C_19_H_24_O_2_N_2_

Obtained from 3.10 g (9.87 mmol) 1-(4′-nitrophenyl)-6,7-dimethoxy-1,2,3,4- tetrahydroisoquinoline (**3m**) and 6.70 g SnCl_2_×2H_2_O. Yield 2.72 g (97%); m.p. of the dihydrochlorohyde 276–277 °C (CH_3_OH); R*_f_* 0.37 (System I). IR (KBr, ν_max_, cm^−1^): 3392, 3321, 3253, 2992, 2961, 2907, 2805, 1634, 1609, 1514, 1460, 1404, 1370, 1350, 1330, 1287, 1252, 1216, 1202, 1174, 1114, 1039, 984, 853, 832.^1^H NMR (400 MHz, DMSO-d_6_ + CCl_4_, δ, ppm, J/Hz): 2.79 (1H, *dt*, *J* = 5.3, 16.3, H_a_-4), 2.92–3.07 (2H, *m*, H_a_-3, H_b_-4), 3.16 (1H, *dt*, *J* = 5.4, 11.5, H_b_-3), 3.53 (3H, *s*, 7-OCH_3_), 3.74 (3H, *s*, 6-OCH_3_), 4. 90 (*br. s*, NH), 5.09 (1H, *s*, H-1), 6.20 (1H, *s*, H-8), 6.50 (2H, *d*, *J* = 8.5, H-3′, 5′), 6.63 (1H, *s*, H-5), 6.88 (1H, *d*, *J* = 8.5, H-2′, 6′). ^13^C NMR (δ, ppm): 26.90 (C-4), 40.30 (C-3), 55.86 (6-OCH_3_), 55.94 (7-OCH_3_), 59.53 (C-1), 111.68 (C-8), 111.99 (C-5), 114.29 (C-3′, 5′), 126.26 (C-4a), 126.26 (C-1′), 127.42 (C-8a), 130 (C-2′, 6′), 147.75 (C-4′), 148.53 (C-7), 149.06 (C-6).

#### 3.3.2. 1-(3′-Aminophenyl)-6,7-dimethoxy-1,2,3,4-tetrahydroisoquinoline (**4b**), C_19_H_24_O_2_N_2_

Obtained from 3.0 g (9.55 mmol) 1-(3′-nitrophenyl)-6,7-dimethoxy-1,2,3,4-tetrahydroisoquinoline (**3n**) and 6.70 g SnCl_2_×2H_2_O. Yield 2.55 g (94%); m.p. of the dihydrochlorohyde 277–278 °C (CH_3_OH); R*_f_* 0.44 (System I).^1^H NMR (400 MHz, DMSO-*d*_6_ + CCl_4_, δ, ppm, J/Hz): 2.96 (1H, *dt*, *J* = 4.9, 16.7, H-4), 3.17–3.29 (3H, *m*, H-3,4), 3.54 (3H, *s*, 7-OCH_3_), 3.75 (3H, *s*, 6-OCH_3_), 5.05 (1H, *br. s,* NH), 5.33 (1H, *s*, H-1), 6. 25 (1H, *s*, H-8), 6.47 (1H, *d*, *J* = 7.7 H-4′), 6.54 (1H, *br. s,* H-2′), 6.57 (1H, *dd*, *J* = 2.5, 7.9, H-6′), 6.72 (1H, *s*, H-5), 7.00 (1H, *t*, *J* = 7.8, H-5′), 7.44 (2H, *br. s,* NH_2_). ^13^C NMR (δ, ppm): 25.05 (C-4), 39.64 (C-3), 55.89 (6-OCH_3_), 55.97 (7-OCH_3_), 58.70 (C-1), 111.47 (C-8), 111.77 (C-5), 115. 29 (C-4′), 115.67 (C-2′), 117.84 (C-6′), 124.14 (C-4a), 125.27 (C-8a), 129.48 (C-5′) 138.05 (C-1′), 148.09 (C-3′), 149.08 (C-7), 149.22 (C-6).

The spectra of all tested compounds were represented in Supplementary Materials Figures S1–S57.

### 3.4. In Silico ADMET/TOPKAT Evaluation

The synthesized series of 1-aryltetrahydroisoquinoline derivatives was subjected to ADMET (Absorption, Distribution, Metabolism, Excretion, and Toxicity) prediction as well as Toxicity Prediction by Komputer Assisted Technology (TOPKAT) using Discovery Studio 2016 (Accelrys Inc., San Diego, CA, USA) with the aim of determining the pharmacokinetic, pharmacodynamic, and toxicity characteristics. Water solubility, plasma protein binding (PPB) prediction, human intestinal absorption (HIA), cytochrome P450 2D6 inhibition (CYP2D6), blood–brain barrier (BBB) penetration, and level of hepatotoxicity were chosen as ADMET parameters. Meanwhile, rat chronic LOAEL (lowest observed adverse effect level), Ames mutagenicity, together with skin and eye irritation were chosen as TOPKAT descriptors [34,35].

### 3.5. Biological Evaluation

Local anesthetic activity was evaluated using the rabbit eye cornea terminal anesthesia test on 2.0–2.5 kg male chinchilla rabbits after introduction of total 0.4 mL (0.2 mL twice with an interval of 30 s) of 1.0 % solutions of the studied substances. Lidocaine was used as an etalon drug. Each substance was tested on three rabbits.

The acute toxicity and resorptive action were determined in white mice weighing 20–24 g by gradual oral administration at doses of 1–5000 mg/kg.

The animals were kept under standard nursery conditions with a natural 12-h light-dark cycle, at an air temperature of 20 ± 2 °C. The animals had unlimited access to food and water. The experiments were performed in accordance with the International Convention for the Protection of Vertebrate Animals used for Experimental and Scientific Purposes (Strasbourg, 1986) [36]. Each substance was tested on 10 mice and LD_50_ was determined graphically by the well-known Litchfield–Wilcoxon method [37,38].

## 4. Conclusions

From the current study, it was concluded that the synthesized compounds showed acceptable pharmacodynamic and pharmacokinetic properties without carcinogenic or mutagenic potential but with variable hepatotoxicity as revealed from ADMET/ TOPKAT in silico study. Furthermore, in vivo studies showed that the introduction of substituents in the C-3′ and C-4′ positions of the phenyl radical of the initial **3a** structure led to a decrease of the acute toxicity of the obtained derivatives. Substitution of hydrogen at the C-2′ position for various groups does not significantly decrease the acute toxicity. The degree of acute toxicity depends on the mutual arrangement and nature of the substituents in the phenyl radical. Among the compounds studied, the least toxic compounds are those having substituents at C-3′ and C-4′ atoms, or at C-3′ hydroxyl, bromo or methoxy, or at C-4′ hydroxyl, nitro group, amino group, or methoxy. The exception is the methoxylation of both positions simultaneously (**3i**). All studied compounds showed high local anesthetic activity on rabbit eyes in the investigated concentrations. Only substances **3r**, **3n**, and **4a** caused eye irritation and redness. All studied derivatives (except **4b**) at a concentration of 1% were more active than lidocaine, providing a longer duration of complete anesthesia. Thus, based on the results obtained from the in silico, local anesthetic, and acute toxicity studies, it was clearly obvious that the investigated compounds (**3c**, **3d**, **3e**, **3l**) need further in-depth future investigations so that they may act as promising drug candidates.

## Figures and Tables

**Table 1:** ADMET characteristics of the series of synthesized 1-aryltetrahydroisoquinoline derivatives.

Compounds	Absorption Level	Solubility Level	BBB Level	PPB Level	CPY2D6	Hepatotoxic	Alog p98	PSA-2D
**3a**	0	2	1	True	NI	TOX	3.021	30.67
**3b**	0	3	2	True	NI	TOX	2.779	51.49
**3c**	0	3	2	True	NI	TOX	2.763	60.42
**3d**	0	2	1	True	NI	TOX	2.989	48.53
**3e**	0	2	1	True	NI	TOX	3.528	51.46
**3f**	0	2	1	True	NI	TOX	3.528	51.46
**3g**	0	2	1	True	NI	TOX	3.737	48.53
**3h**	0	2	2	True	NI	TOX	3.511	60.42
**3i**	0	2	2	True	NI	TOX	2.789	48.53
**3j**	0	2	1	True	NI	TOX	3.538	48.53
**3k**	0	2	1	True	NI	TOX	3.454	48.53
**3l**	0	2	2	True	NI	TOX	3.511	60.42
**3m**	0	2	2	True	NI	TOX	2.916	73.49
**3n**	0	2	2	True	NI	TOX	2.916	73.49
**3o**	0	2	1	True	NI	TOX	3.770	30.67
**3p**	0	2	1	True	NI	TOX	3.686	30.67
**3q**	0	3	2	True	NI	TOX	2.275	57.21
**3r**	0	3	2	True	NI	TOX	2.275	57.21
**4a**	0	2	1	True	NI	TOX	3.021	30.67
**4b**	0	3	2	True	NI	TOX	2.779	51.49

0, 1, 2, and 3 indicates good, moderate, low, and very low absorption, respectively; 0, 1, 2, 3, 4, and 5 indicates extremely low, very low but possible, low, good, optimal, and too soluble, respectively; 0, 1, 2, 3, and 4 denote very high, high, medium, low, and undefined, penetration via BBB respectively. PBB—plasma protein binding, FALSE means less than 90%, TRUE means more than 90; NI—non-inhibitor; NT—non-toxic.

**Table 2:** In silico TOPKAT evaluation of the series of synthesized 1-aryltetrahydroisoquinoline derivatives.

Compounds	Ames Prediction	Rat Chronic LOAEL	Skin Irritancy	Ocular Irritancy	Rat Female FDA	Rat Male FDA
**3a**	Non-mutagen	0.026	None	Mild	Non-carcinogen	Non-carcinogen
**3b**	Non-mutagen	0.041	None	Mild	Non-carcinogen	Non-carcinogen
**3c**	Non-mutagen	0.022	None	Severe	Non-carcinogen	Non-carcinogen
**3d**	Non-mutagen	0.012	None	Severe	Non-carcinogen	Non-carcinogen
**3e**	Non-mutagen	0.019	None	Severe	Non-carcinogen	Non-carcinogen
**3f**	Non-mutagen	0.010	None	Severe	Non-carcinogen	Non-carcinogen
**3g**	Non-mutagen	0.007	None	Mild	Non-carcinogen	Non-carcinogen
**3h**	Non-mutagen	0.013	None	Mild	Non-carcinogen	Non-carcinogen
**3i**	Non-mutagen	0.008	Mild	Mild	Non-carcinogen	Non-carcinogen
**3j**	Non-mutagen	0.005	None	Mild	Non-carcinogen	Non-carcinogen
**3k**	Non-mutagen	0.004	None	Mild	Non-carcinogen	Non-carcinogen
**3l**	Non-mutagen	0.013	None	Severe	Non-carcinogen	Non-carcinogen
**3m**	Non-mutagen	0.006	Mild	Severe	Non-carcinogen	Non-carcinogen
**3n**	Non-mutagen	0.008	Mild	Mild	Non-carcinogen	Non-carcinogen
**3o**	Non-mutagen	0.012	None	Mild	Non-carcinogen	Non-carcinogen
**3p**	Non-mutagen	0.009	None	Severe	Non-carcinogen	Non-carcinogen
**3q**	Non-mutagen	0.018	None	Mild	Non-carcinogen	Non-carcinogen
**3r**	Non-mutagen	0.022	None	Mild	Non-carcinogen	Non-carcinogen
**4a**	Non-mutagen	0.026	None	Mild	Non-carcinogen	Non-carcinogen
**4b**	Non-mutagen	0.041	None	Mild	Non-carcinogen	Non-carcinogen

**Table 3:** Acute toxicity of the studied compounds in mice after intragastric administration (N = 10).

Compound	LD_50_, mg/kg	Compound	LD_50_, mg/kg
**3a**	280 (250.0 ± 313.6)	**3k**	520 (456.1 ± 592.8)
**3b**	375 (334.8 ± 420.0)	**3l**	1560 (1392.8 ± 1747.2)
**3c**	980 (867.2 ± 1107.4)	**3m**	1950 (1695.6 ± 2242.5)
**3d**	670 (587.7 ± 763.8)	**3n**	525 (460.5 ± 598.5)
**3e**	3850 (3347.8 ± 4427.5)	**3o**	710 (628.3 ± 80.,3)
**3f**	305 (270 ± 344.6)	**3p**	300 (265.4 ± 339.0)
**3g**	290 (258.9 ± 330.6)	**3q**	1250 (110.6 ± 1412)
**3h**	550 (482.4 ± 627.0)	**3r**	740 (649.1 ± 843.6)
**3i**	280 (243.4 ± 322.0)	**4a**	1050 (929.2 ± 1176.0)
**3j**	730 (651.7 ± 817.6)	**4b**	750 (657.8 ± 855.0)

**Table 4:** Local anesthetic activity of the studied compounds in rabbit corneal model in compared to lidocaine.

Compound ^a^	Onset of Anesthesia (min)	Duration of Complete Anesthesia (%)	Total Duration of Anesthesia (min)
**Lidocaine**	5.0 ± 0.5	100	52.7 ± 4.9
**3a**	3.0 ± 1.1	+50	53.0 ± 4.6
**3b**	1.0 ± 0.4	+50	44.0 ± 1.5
**3c**	3.0 ± 0.8	+40	70.0 ± 4.5
**3d**	2.0 ± 0.7	+60	70.0 ± 2.1
**3e**	5.0 ± 0.9	+49	80.0 ± 3.2
**3f**	1.0 ± 0.5	+160	64.0 ± 3.8
**3g**	2.0 ± 1.0	+90	44.0 ± 3.5
**3h**	1.0 ± 0.4	+170	40.0 ± 3.2
**3i**	2.0 ± 0.4	+80	57.0 ± 3.7
**3j**	2.0 ± 1.0	+90	52.0 ± 4.0
**3k**	2.0 ± 1.2	+90	43.0 ± 4.5
**3l**	5.0 ± 1.2	+140	89.0 ± 3.0
**3m**	3.0 ± 0.4	+80	48.0 ± 2.8
**3n**	3.0 ± 1.0	+260	37.0 ± 3.2
**3o**	2.0 ± 0.4	+130	115.0 ± 3.8
**3p**	3.0 ± 0.8	+300	67.0 ± 3.6
**3q**	2.0 ± 0.6	+220	45.0 ± 4.1
**3r**	2.0 ± 0.6	+150	35.0 ± 3.4
**4a**	2.0 ± 0.7	+40	25.0 ± 4.0
**4b**	3.0 ± 0.7	+0	44.0 ± 4.1

^a^ All compounds were in aqueous solution at 1% concentration. The values of complete anesthesia were expressed as percentage of the anesthetic activity of lidocaine (=100%).

## Data Availability

Data are available in the manuscript.

## Supplementary Materials

The following supporting information can be downloaded at: https://www.mdpi.com/article/10.3390/molecules28020477/s1, Figures S1–S57: NMR, ESI-MS, and IR spectra of the 20 derivatives of 1-aryl-6,7-dimethoxy-1,2,3,4-tetrahydroisoquinolines (**3a–r, 4a–b**).

## Sample Availability

Samples are available from the authors.
