# Supplementary material for: Evaluation of the Local Anesthetic Activity, Acute Toxicity, and Structure–Toxicity Relationship in Series of Synthesized 1-Aryltetrahydroisoquinoline Alkaloid Derivatives In Vivo and In Silico
Source: Molecules. 2023 Jan 4;28(2):477. doi: 10.3390/molecules28020477 (PMC9864514; doi:10.3390/molecules28020477)
Supplement: Supplementary file 1 [file molecules-28-00477-s001.zip › molecules-2066398-supplementary.pdf]

Evaluation of the Local Anesthetic Activity, Acute Toxicity and Structure-Toxicity Relationship in Series of Synthesized 1-Aryltetrahydroisoquinoline Alkaloid Derivatives in Vivo and in Silico

Azizbek A. Azamatov [1], Sherzod N. Zhurakulov [1,2], Valentina I. Vinogradova [1], Firuza Tursunkhodzhaeva [1], Roaa M. Khinkar [3], Rania T. Malatani [3], Mohammed M. Aldurdunji [4], Antonio Tiezzi [5] and Nilufar Z. Mamadalieva [1,*]

1 Institute of the Chemistry of Plant Substances, Academy of Sciences of the Republic of Uzbekistan, Tashkent 100170, Mirzo Ulugbek str. 77, Uzbekistan
2 National University of Uzbekistan named after Mirzo Ulugbek, Tashkent 100174, University str. 4, Uzbekistan
3 Department of Pharmacy Practice, Faculty of Pharmacy, King Abdulaziz University, Jeddah 21589, Saudi Arabia
4 Department of Clinical Pharmacy, College of Pharmacy, Umm Al-Qura University, P.O. Box 13578 Makkah 21955, Saudi Arabia
5 Department for the Innovation in Biological, Agro-food and Forestal Systems, Tuscia University, 01100 Viterbo, Italy
* Correspondence: nmamadalieva@yahoo.com

Supplementary data

Figure S1. ^{1}H NMR spectrum of compound **3a.**
Figure S2. ^{13}C NMR spectrum of compound **3a.**
Figure S3. IR spectrum of compound **3a**.
Figure S4. ^{1}H NMR spectrum of compound **3b.**
Figure S5. ^{13}C NMR spectrum of compound **3b**.
Figure S6. IR spectrum of compound **3b**.
Figure S7. ^{1}H NMR spectrum of compound **3c.**
Figure S8. ^{13}C NMR spectrum of compound **3c**.
Figure S9. ^{1}H NMR spectrum of compound **3d.**
Figure S10. ^{13}C NMR spectrum of compound **3d**.
Figure S11. HSQC spectrum of compound **3d.**
Figure S12. HMBC spectrum of compound **3d.**
Figure S13. COSY spectrum of compound **3d.**
Figure S14. IR spectrum of compound **3d**.
Figure S15. +ESI-mass- spectrum *m/z* 330 $(M+H)^{+}$ of compound **3d**.
Figure S16. +ESI-mass- spectrum of compound **3d**.
Figure S17. ^{1}H NMR spectrum of compound **3e**.
Figure S18. ^{13}C NMR spectrum of compound **3e**
Figure S19. ^{1}H NMR spectrum of compound **3f**.
Figure S20. ^{13}C NMR spectrum of compound **3f**.
Figure S21. IR spectrum of compound **3f**.
Figure S22. ^{1}H NMR spectrum of compound **3g**.
Figure S23. ^{13}C NMR spectrum of compound **3g**.
Figure S24. IR spectrum of compound **3g**.
Figure S25. ^{1}H NMR spectrum of compound **3h**.
Figure S26. ^{13}C NMR spectrum of compound **3h**.
Figure S27. +ESI-mass- spectrum *m/z* 394 $(M+H)^{+}$ from the isotope bromine 79, of compound **3h**
Figure S28. +ESI-mass- spectrum *m/z* 396 $(M+H)^{+}$ from the isotope bromine 81, of compound **3h**
Figure S29. ^{1}H NMR spectrum of compound **3i**.
Figure S30. ^{13}C NMR spectrum of compound **3i**.

Figure S31. Diff-NOE spectrum of compound **3i.**
Figure S32. COSY spectrum of compound **3i.**
Figure S33. Dept spectrum of compound **3i.**
Figure S34. Diff-decpl spectrum of compound **3i.**
Figure S35. IR spectrum of compound **3i**.
Figure S36. ^{1}H NMR spectrum of compound **3j**.
Figure S37. ^{13}C NMR spectrum of compound **3j**.
Figure S38. IR spectrum of compound **3j**
Figure S39. ^{1}H NMR spectrum of compound **3k**
Figure S40. ^{1}H NMR spectrum of compound **3l**.
Figure S41. ^{13}C NMR spectrum of compound **3l**.
Figure S41. ^{13}C NMR spectrum of compound **3l**.
Figure S43. IR spectrum of compound **3m**.
Figure S44. ^{1}H NMR spectrum of compound **3n**
Figure S45. IR spectrum of compound **3n**.
Figure S46. ^{1}H NMR spectrum of compound **3o**.
Figure S47. ^{13}C NMR spectrum of compound **3o**.
Figure S48. ^{1}H NMR spectrum of compound **3p**
Figure S49. IR spectrum of compound **3n**.
Figure S50. ^{1}H NMR spectrum of compound **3q**.
Figure S51. IR spectrum of compound **3q**.
Figure S52. ^{1}H NMR spectrum of compound **3r**.
Figure S53. ^{1}H NMR spectrum of compound **4a**.
Figure S54. ^{13}C NMR spectrum of compound **4a**.
Figure S55. IR spectrum of compound **4a**.
Figure S56. ^{1}H NMR spectrum of compound **4b**.
Figure S57. ^{13}C NMR spectrum of compound **4b**.

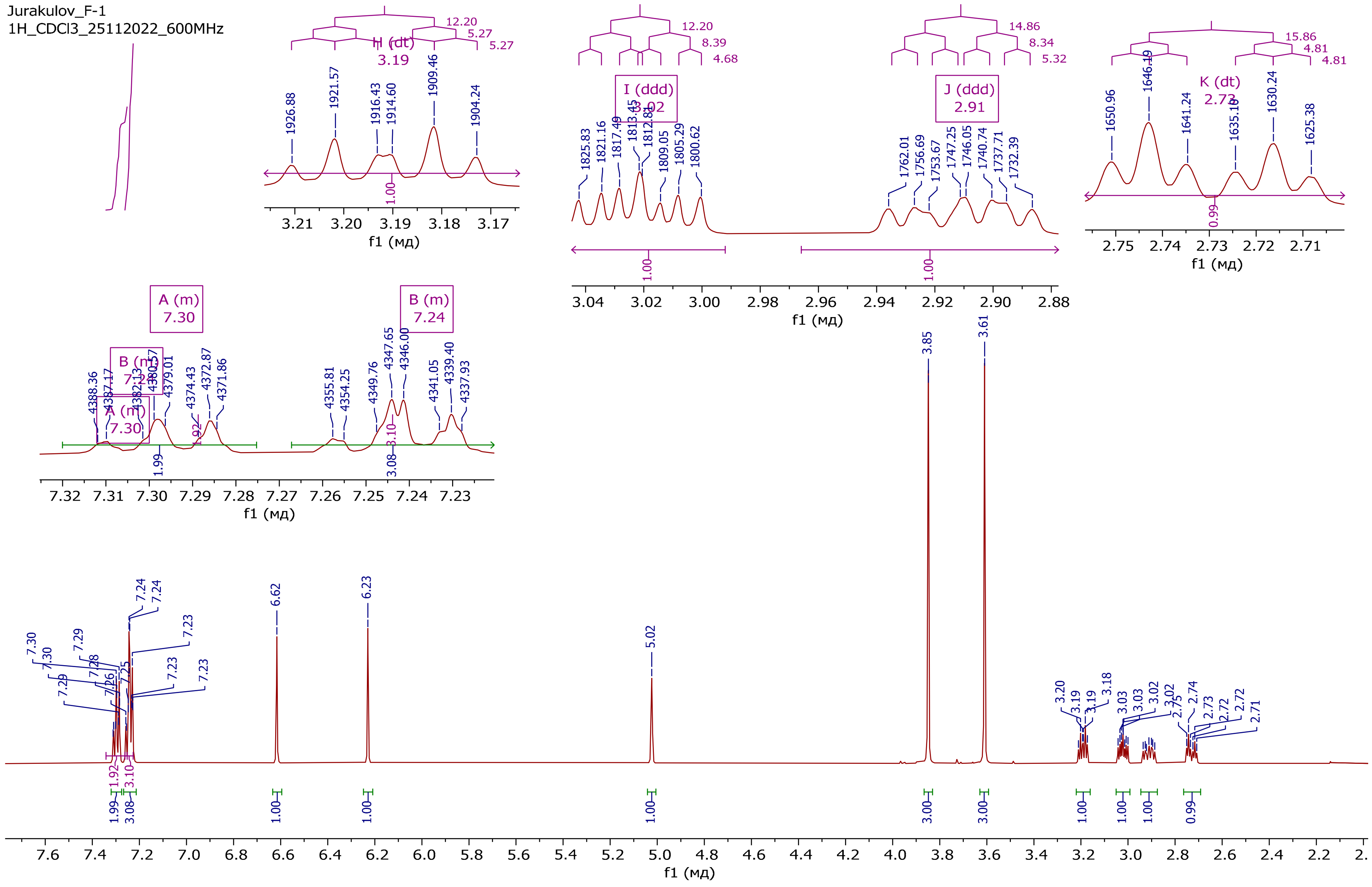

Figure S1. ^{1}H NMR spectrum of compound **3a.**

Jurakulov_F-1
13C_CDCl3_25112022_600MHz

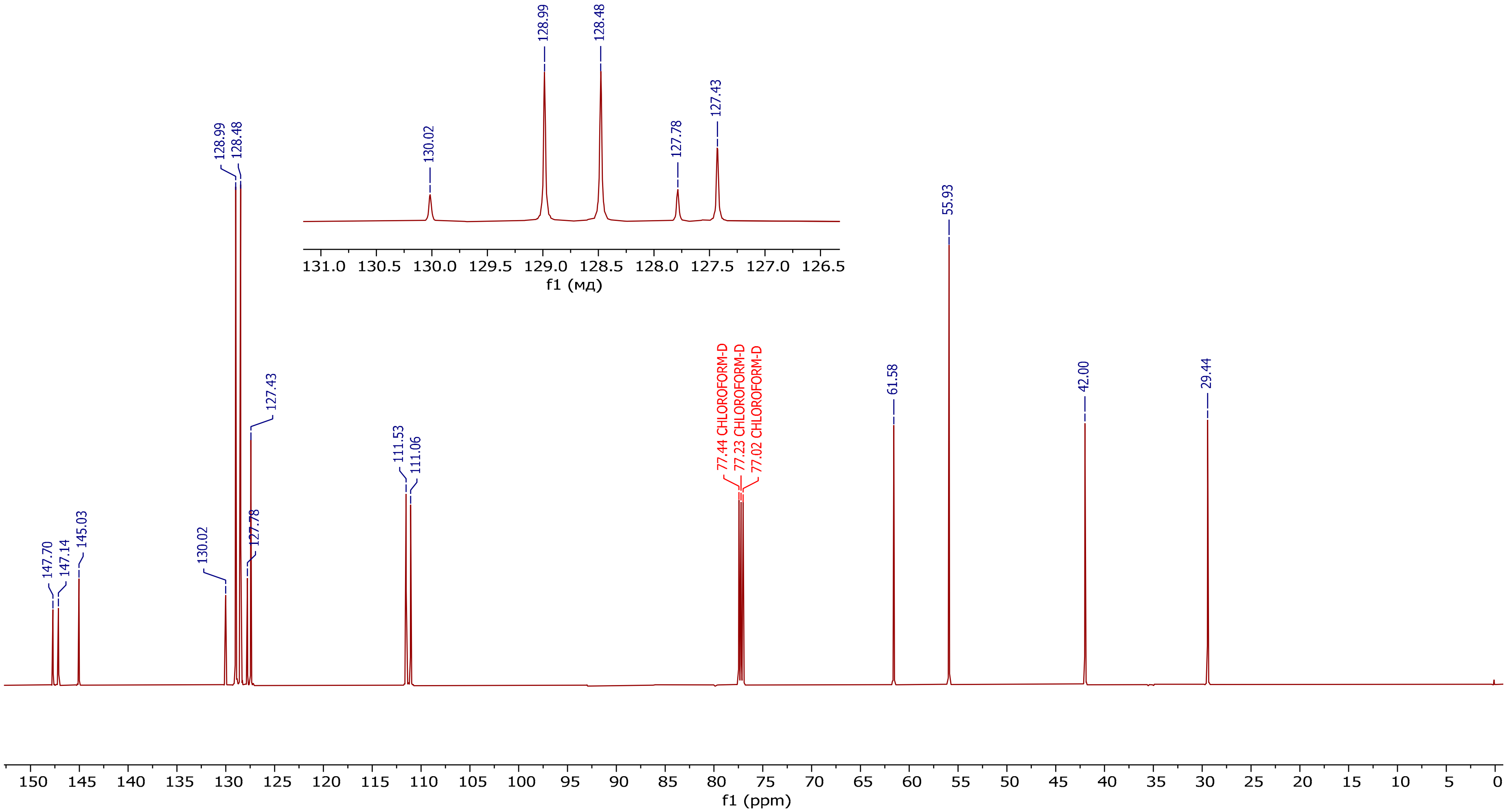

Figure S2. ^{13}C NMR spectrum of compound **3a.**

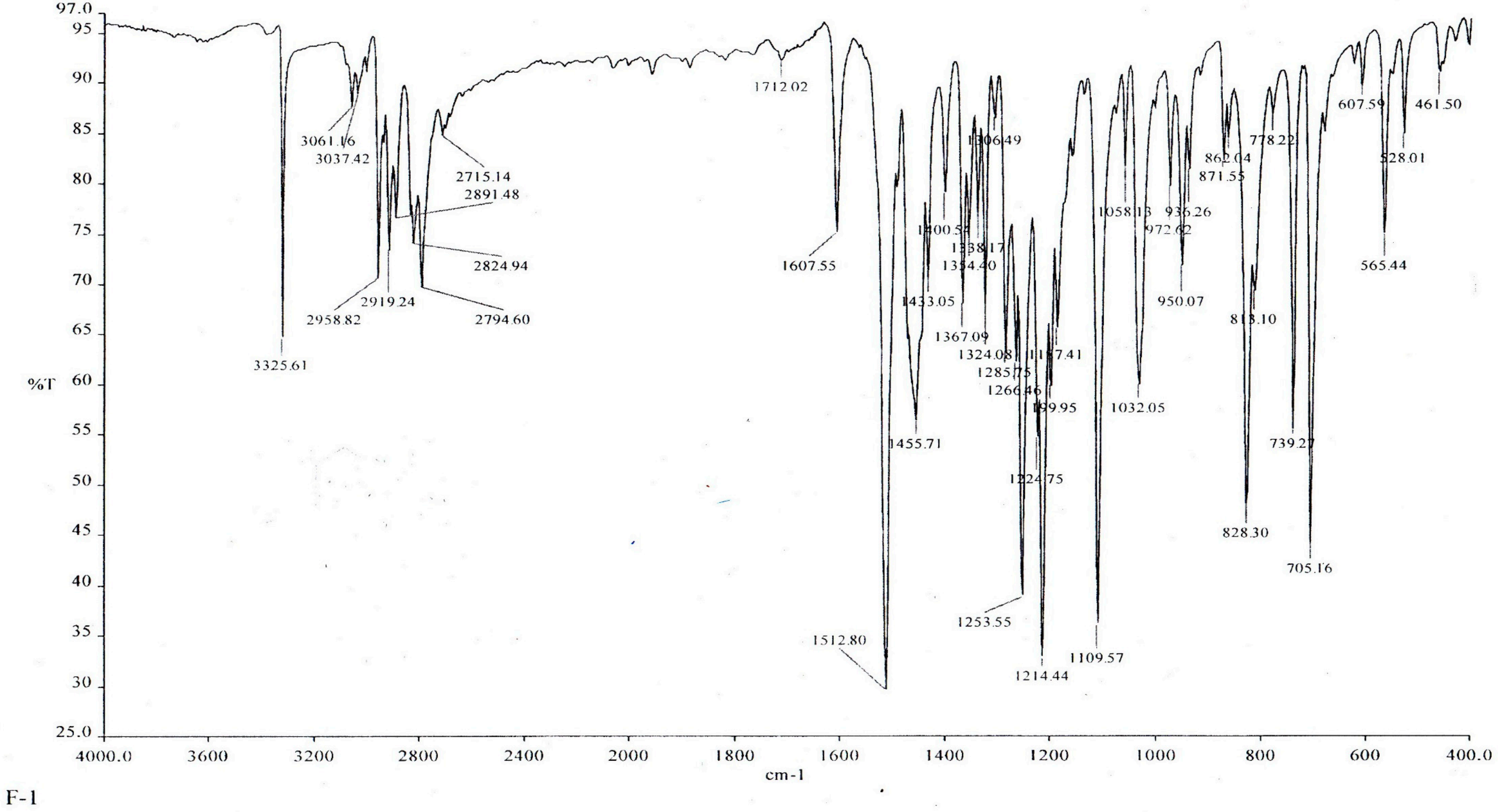

Figure S3. IR spectrum of compound **3a**.

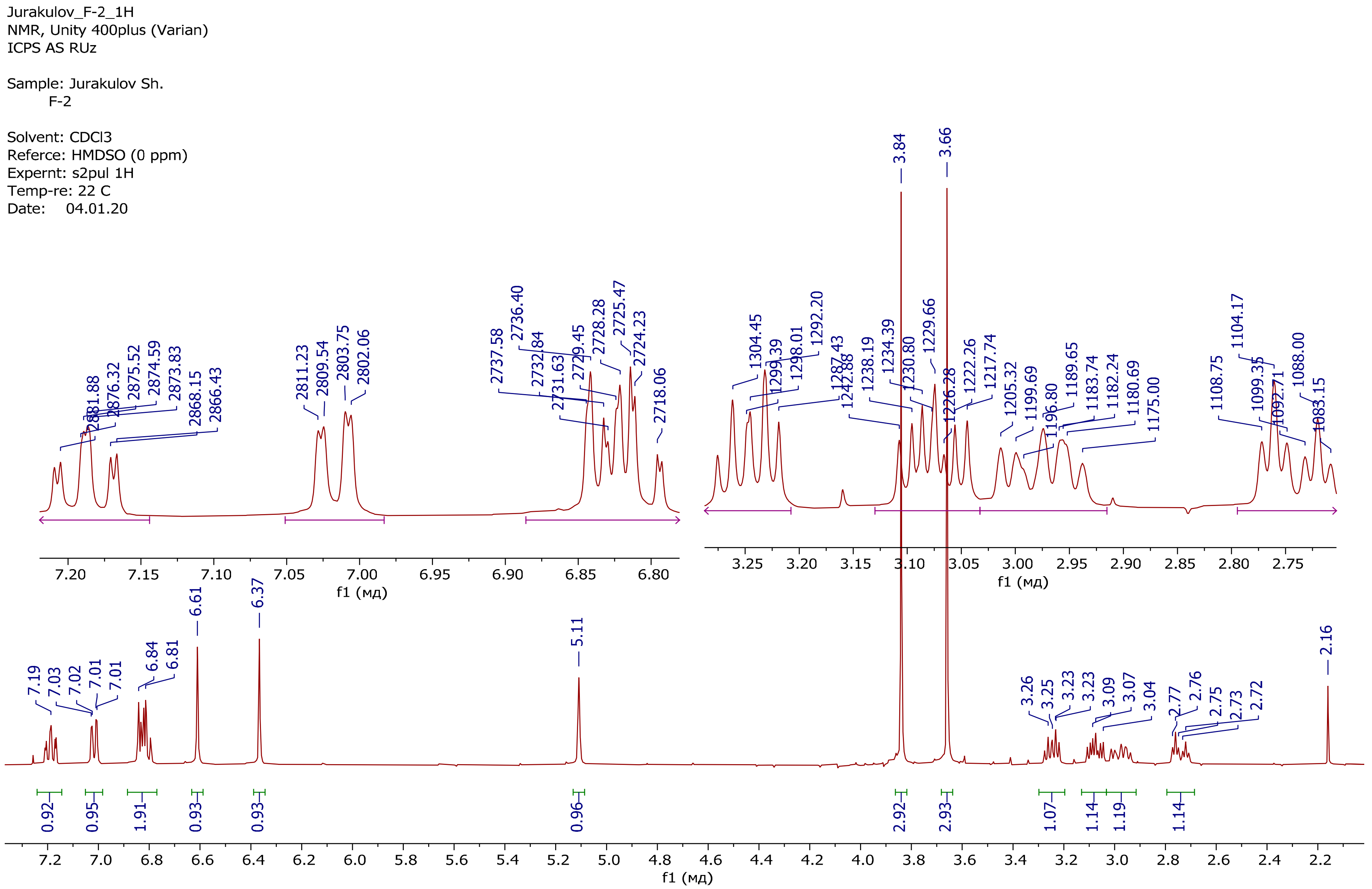

Figure S4. ^{1}H NMR spectrum of compound **3b.**

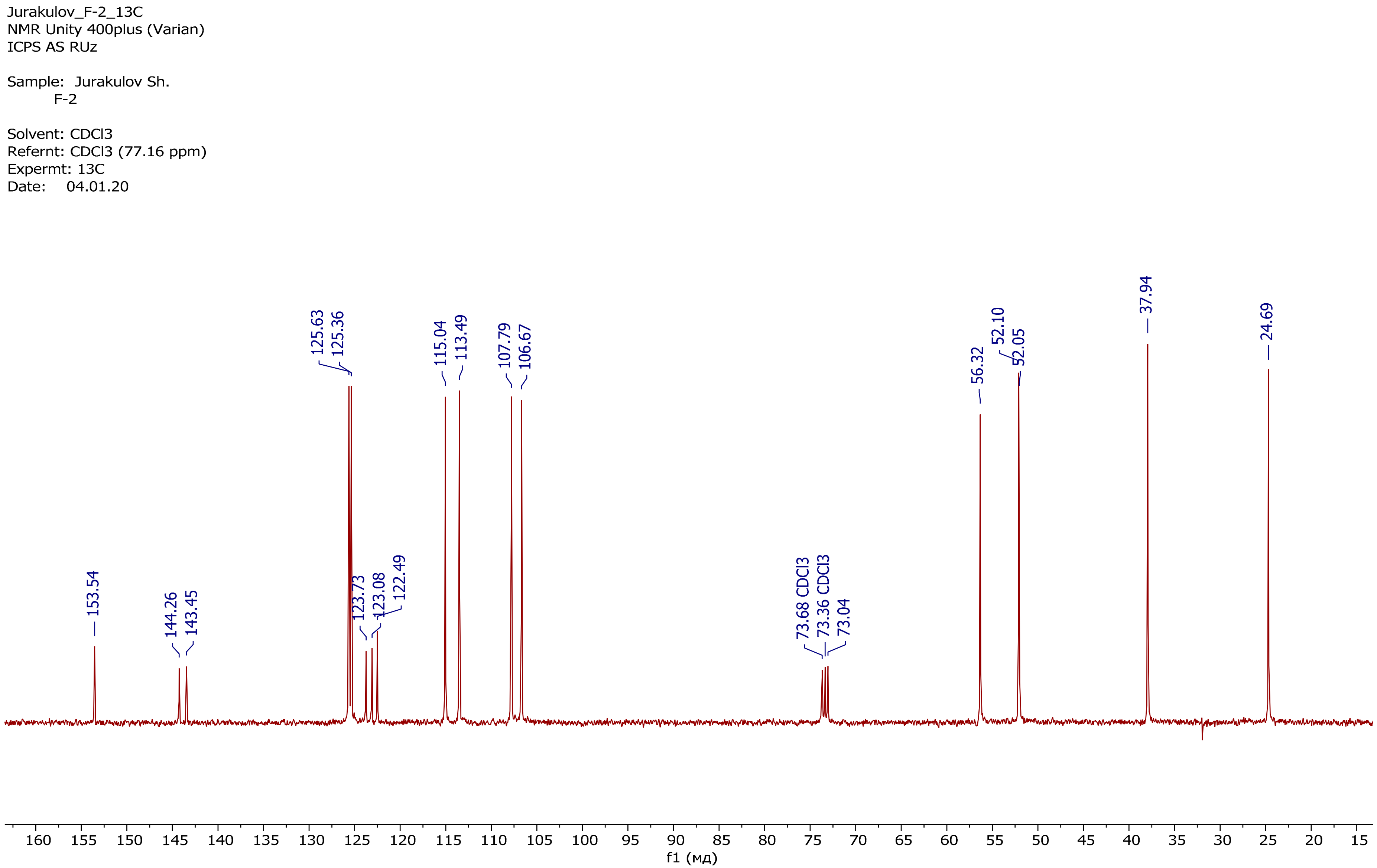

Figure S5. ^{13}C NMR spectrum of compound **3b**.

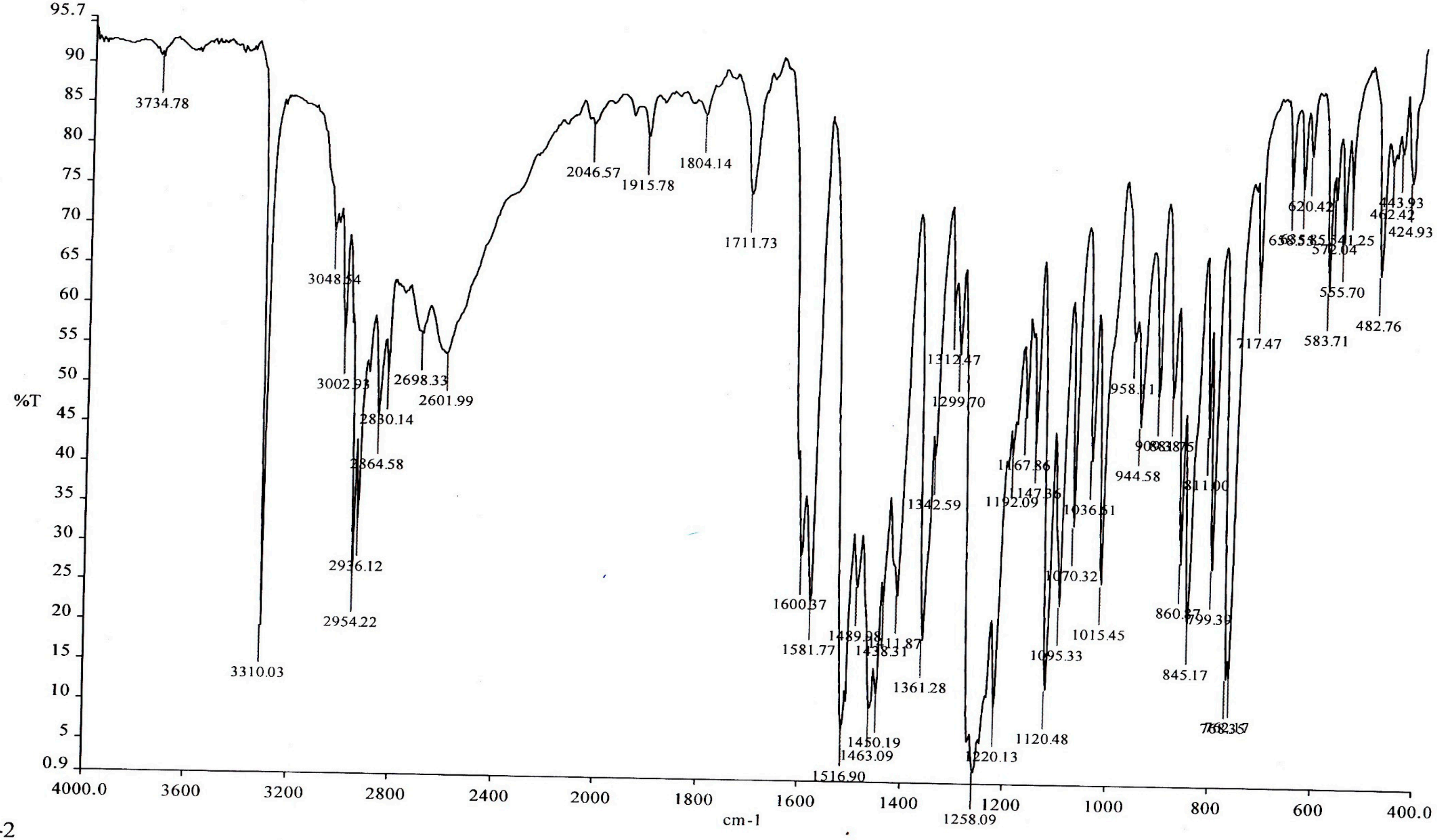

F-2

Figure S6. IR spectrum of compound **3b**.

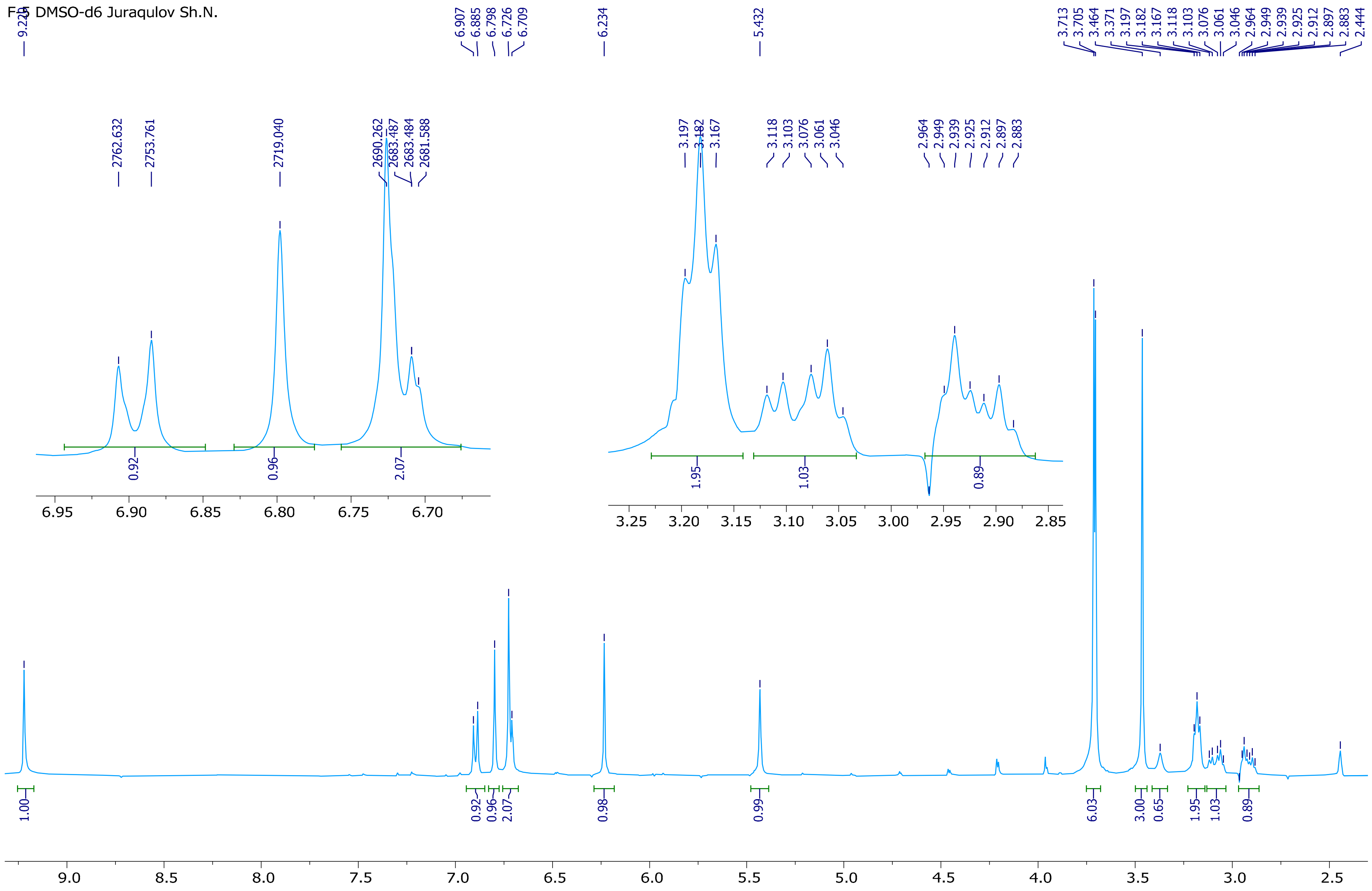

Figure S7. ^{1}H NMR spectrum of compound **3c.**

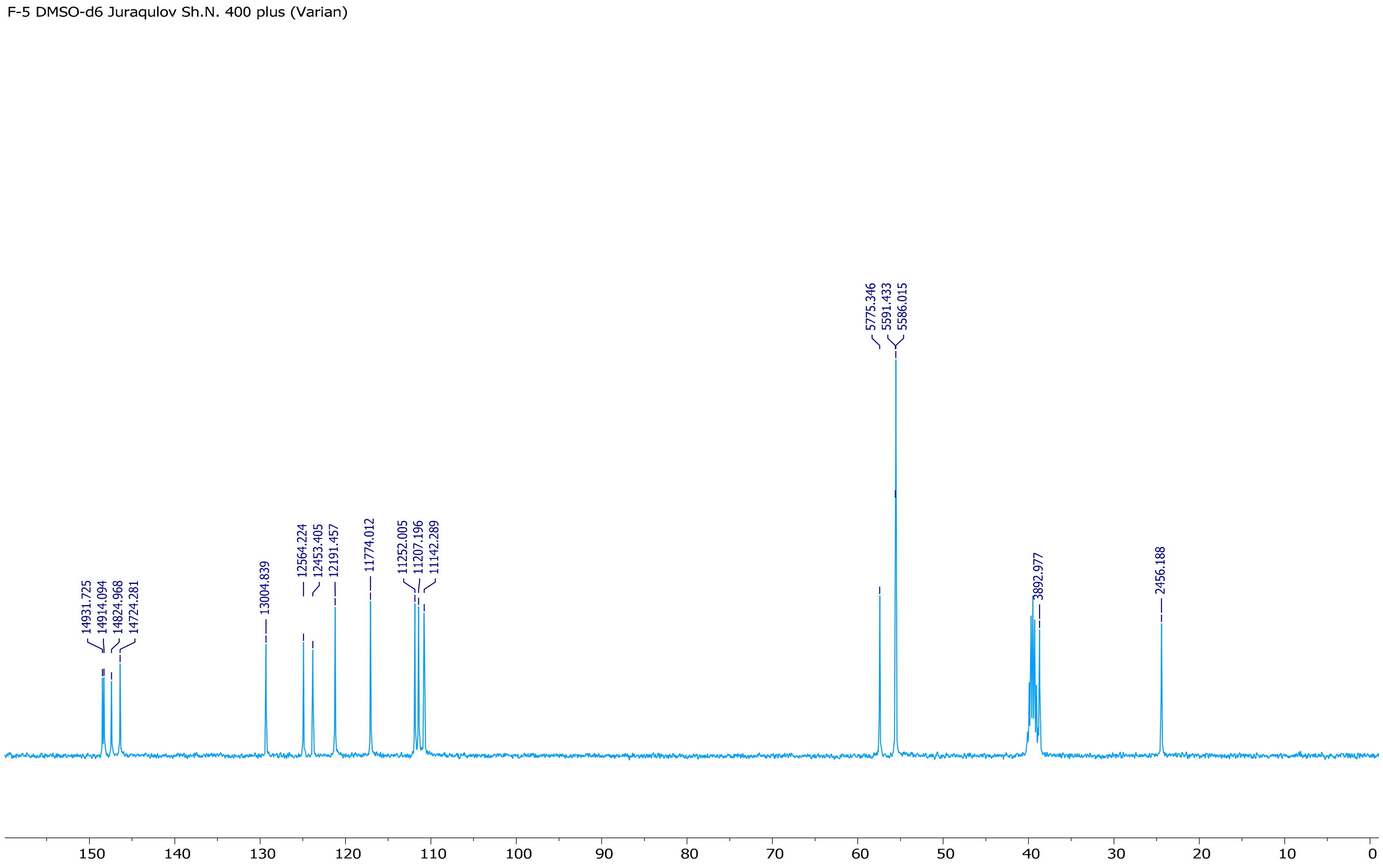

Figure S8. ^{13}C NMR spectrum of compound **3c**.

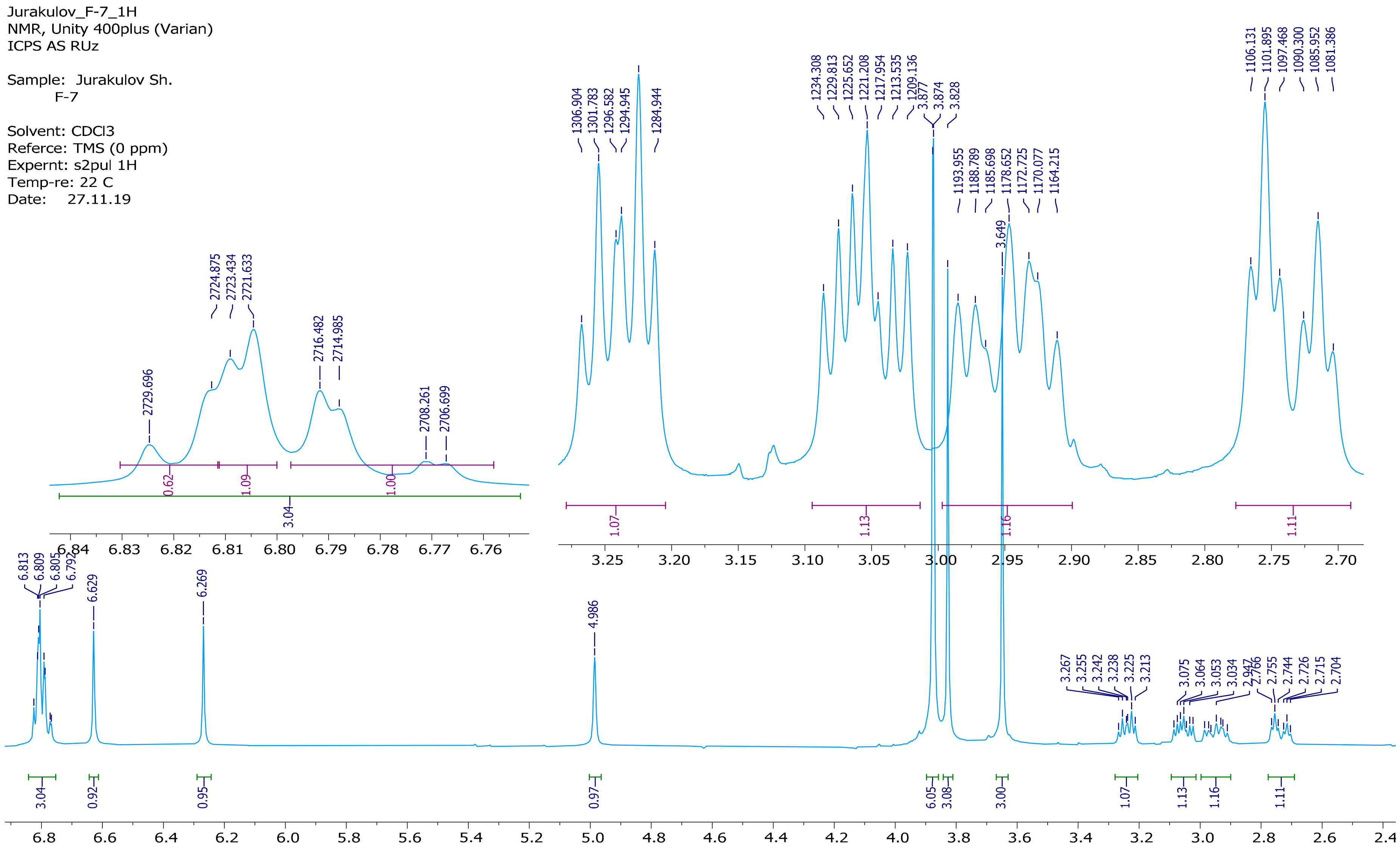

Figure S9. ^{1}H NMR spectrum of compound **3d.**

Jurakulov_F-7_13C
NMR Unity 400plus (Varian)
ICPS AS RUz

Sample: Jurakulov Sh.
F-7

Solvent: CDCl3
Refernt: CDCl3 (77.16 ppm)
Expermt: s2pul 13C
Date: 27.11.19

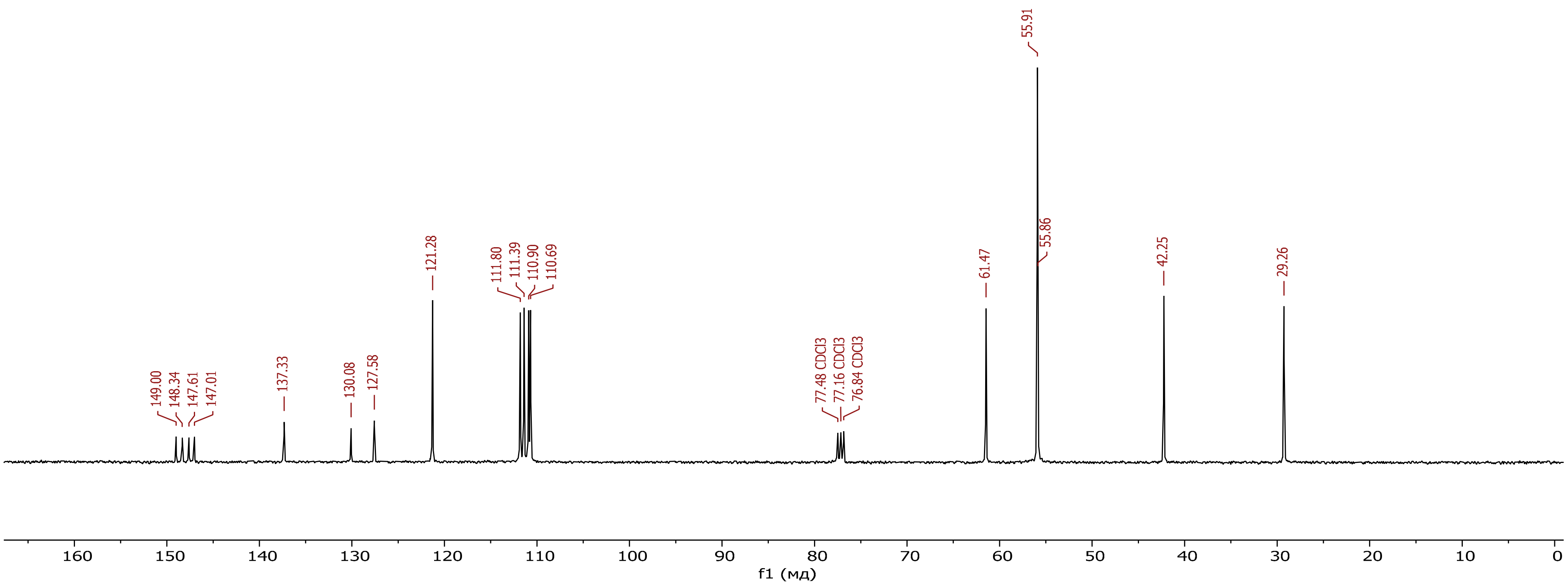

Figure S10. ^{13}C NMR spectrum of compound **3d**.

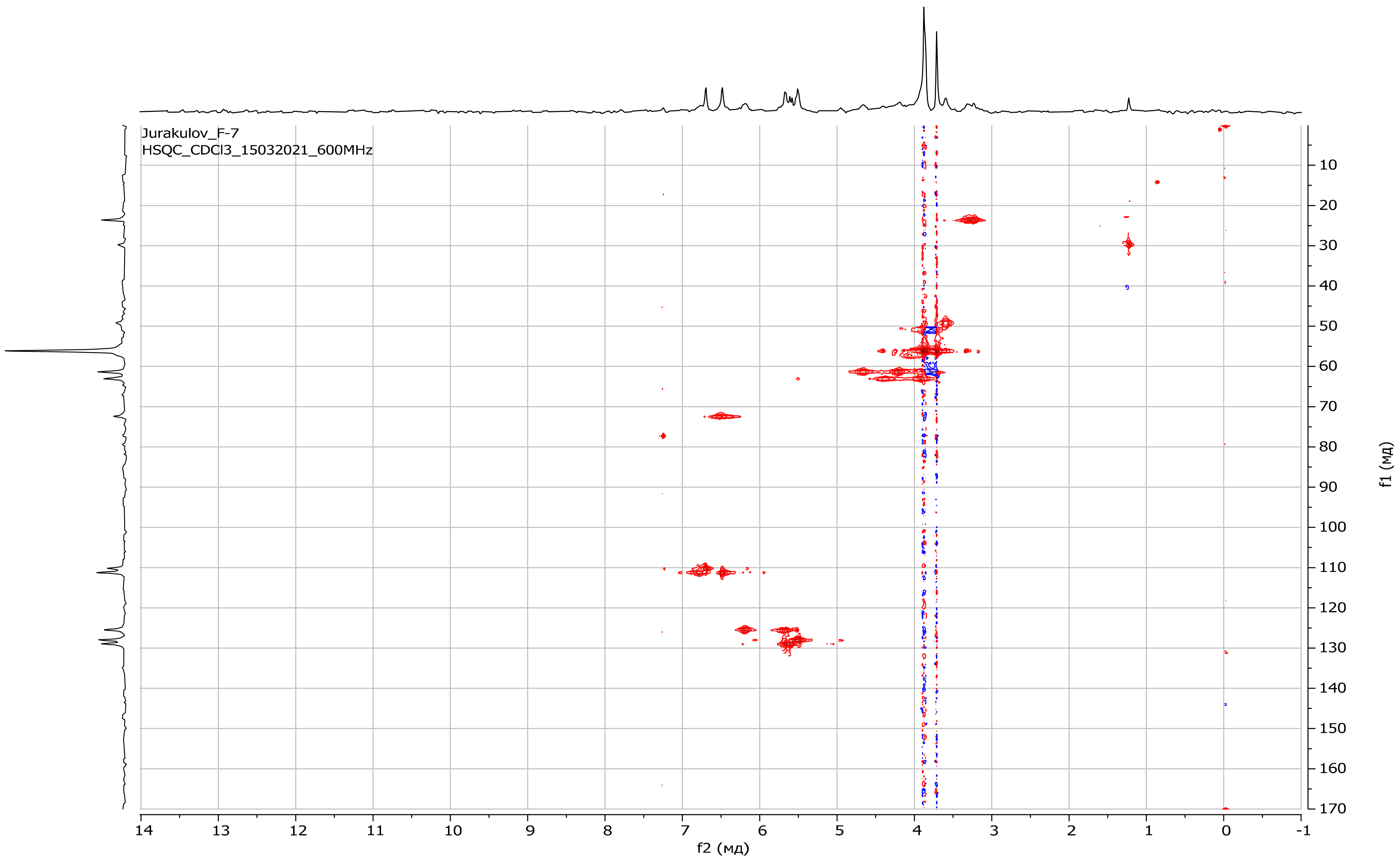

Figure S11. HSQC spectrum of compound **3d.**

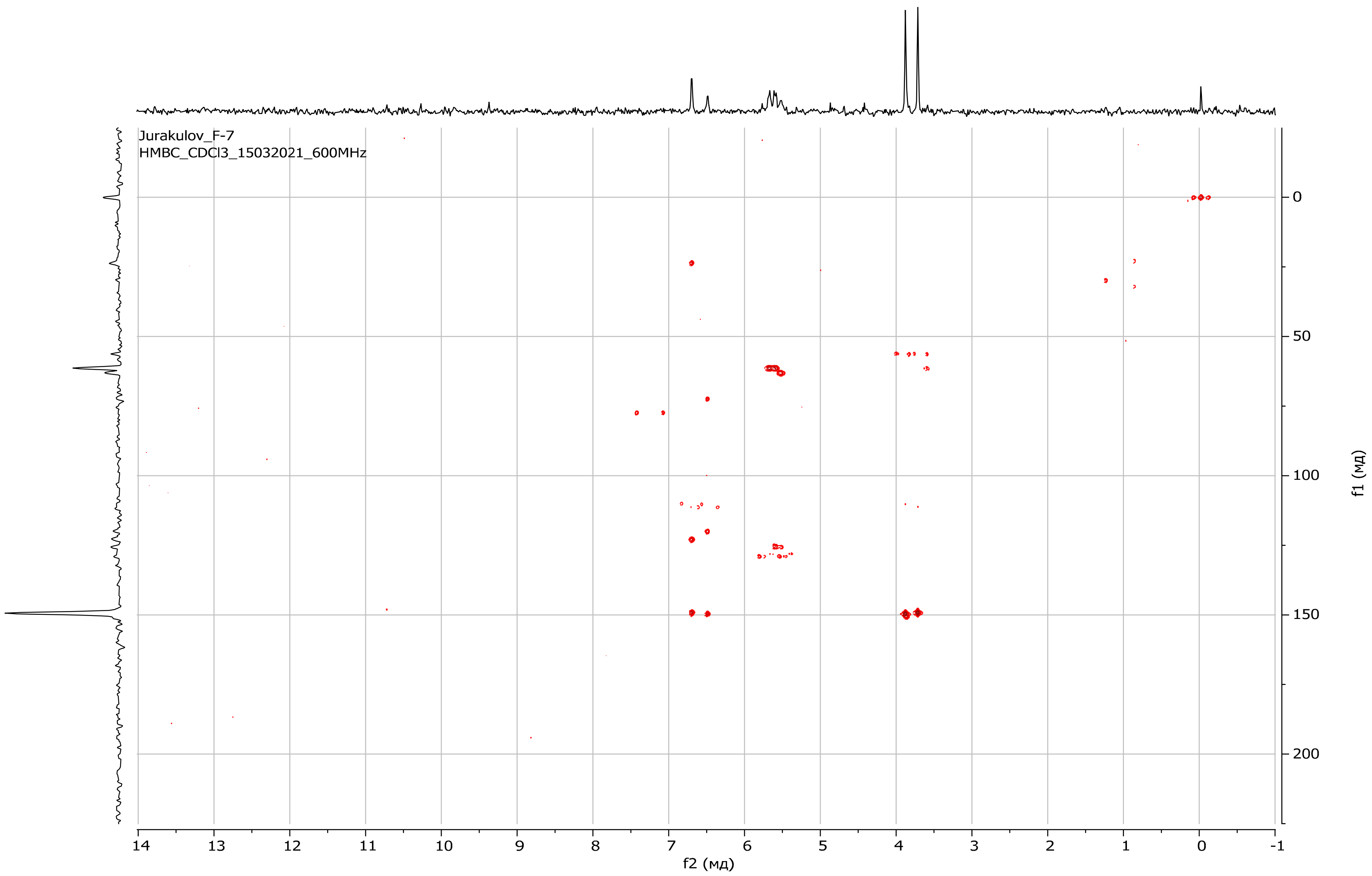

Figure S12. HMBC spectrum of compound **3d.**

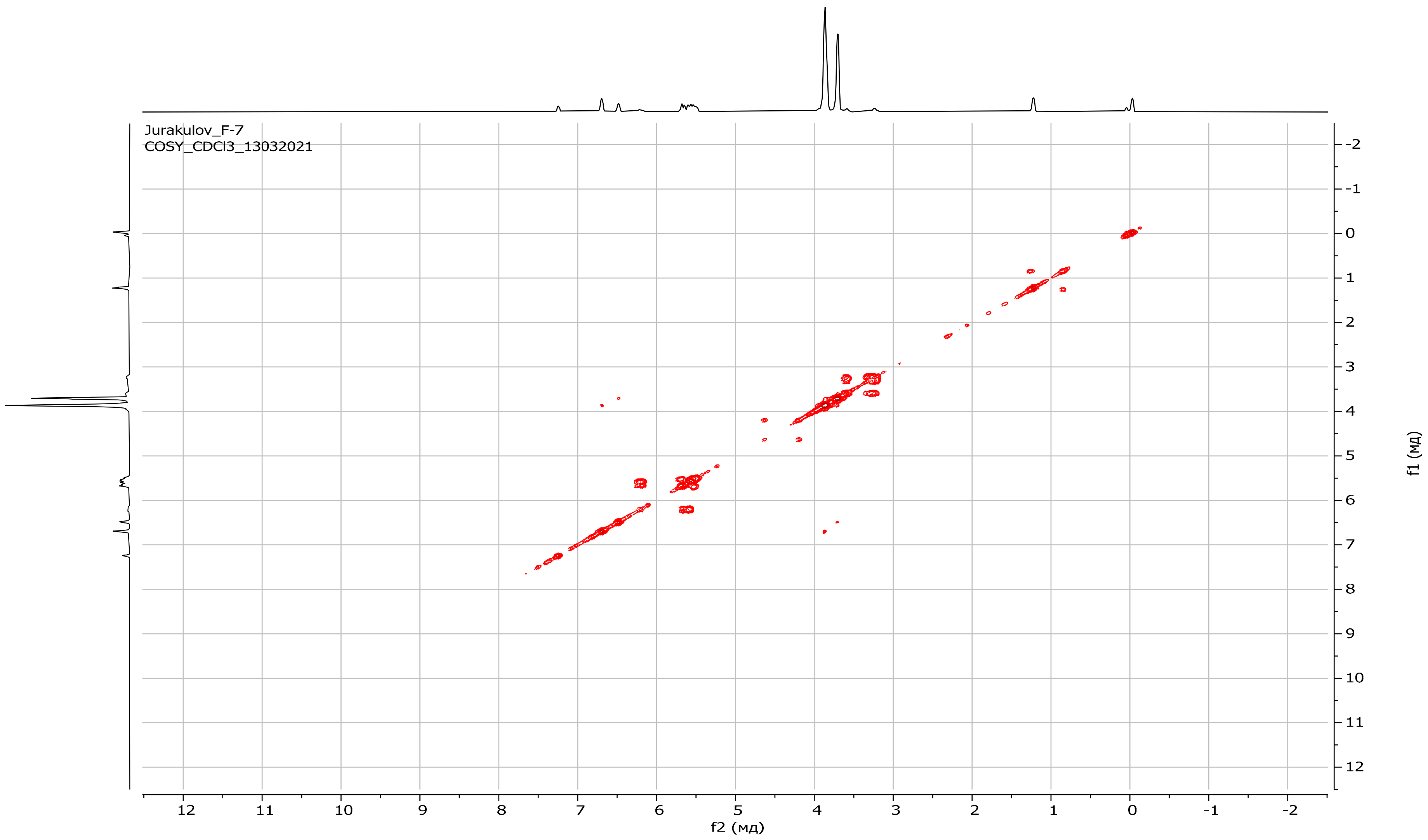

Figure S13. COSY spectrum of compound **3d.**

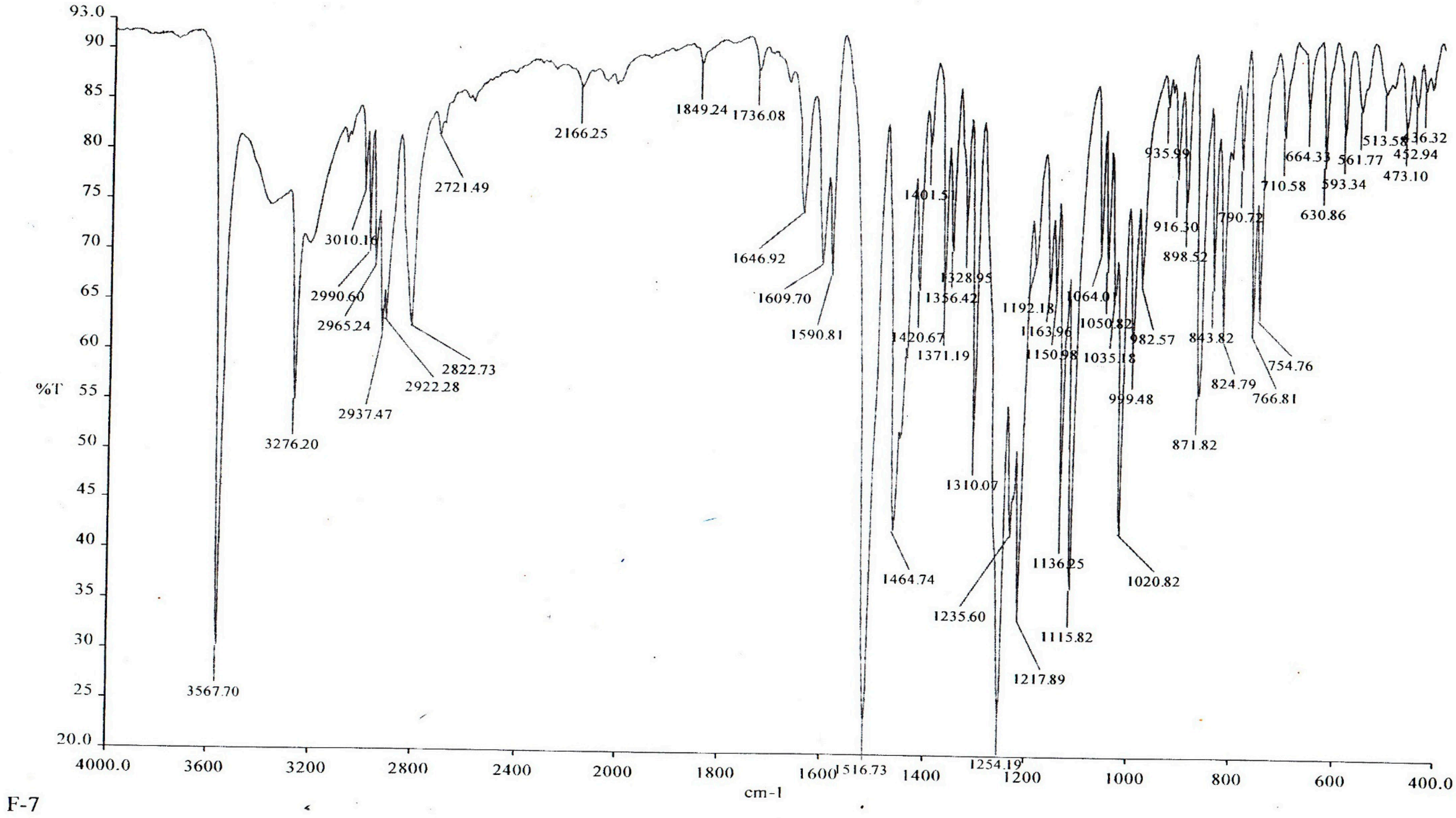

Figure S14. IR spectrum of compound **3d**.

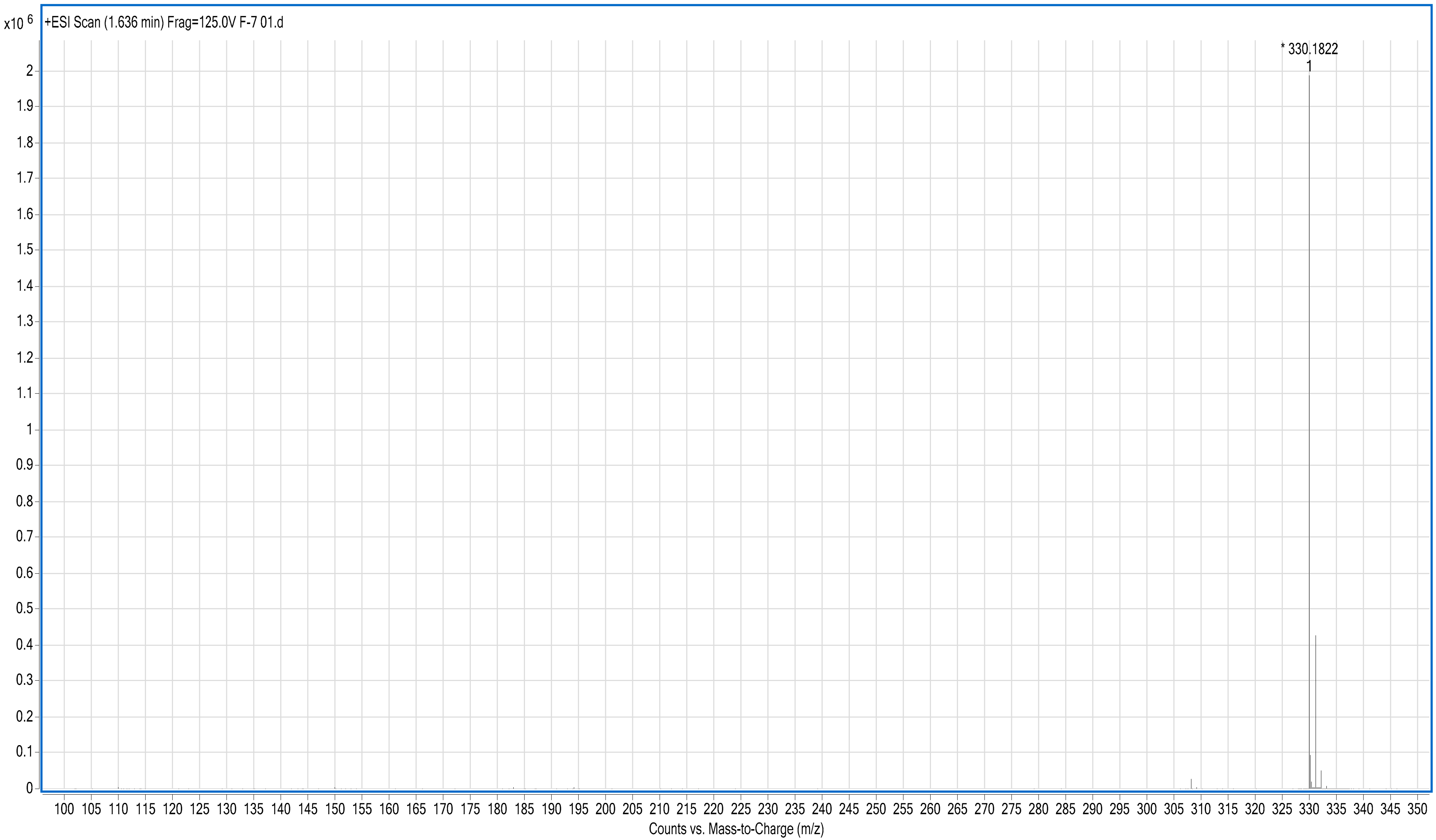

Figure S15. +ESI- mass- spectrum of compound **3d** *(m/z* 330 $[M+H]^+$).

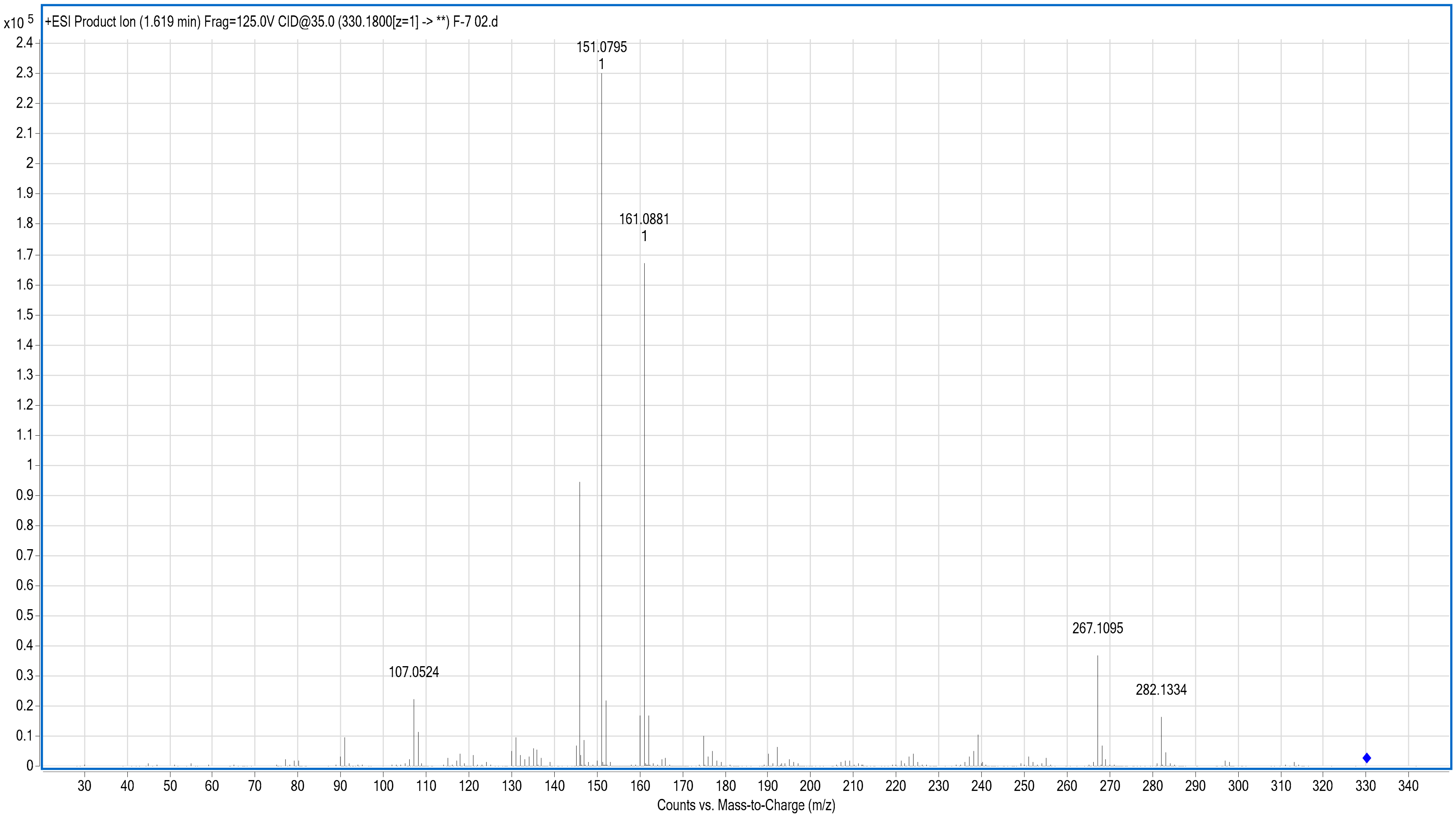

Figure S16. +ESI-mass- spectrum of compound **3d**.

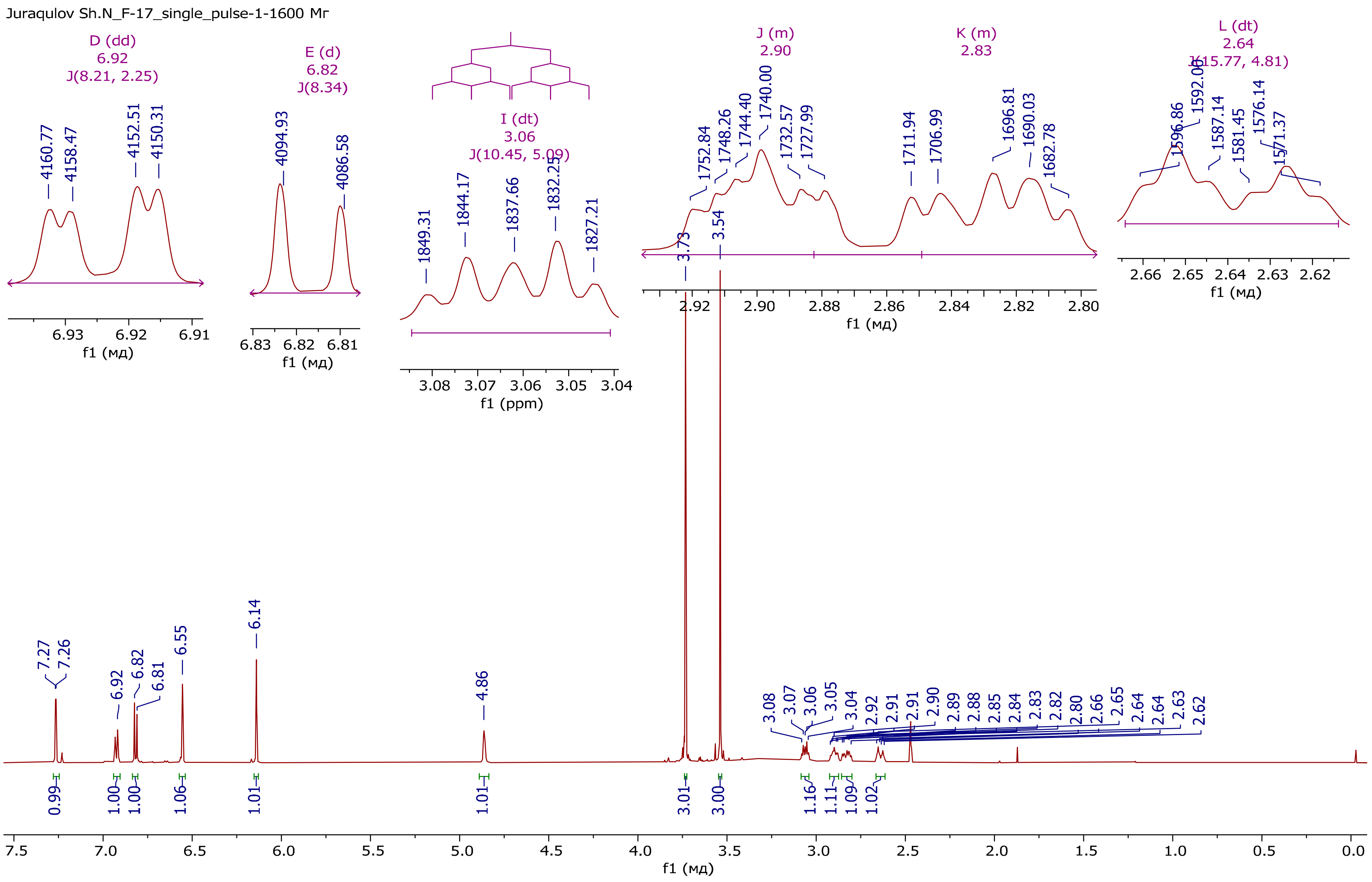

Figure S17. ^{1}H NMR spectrum of compound **3e**.

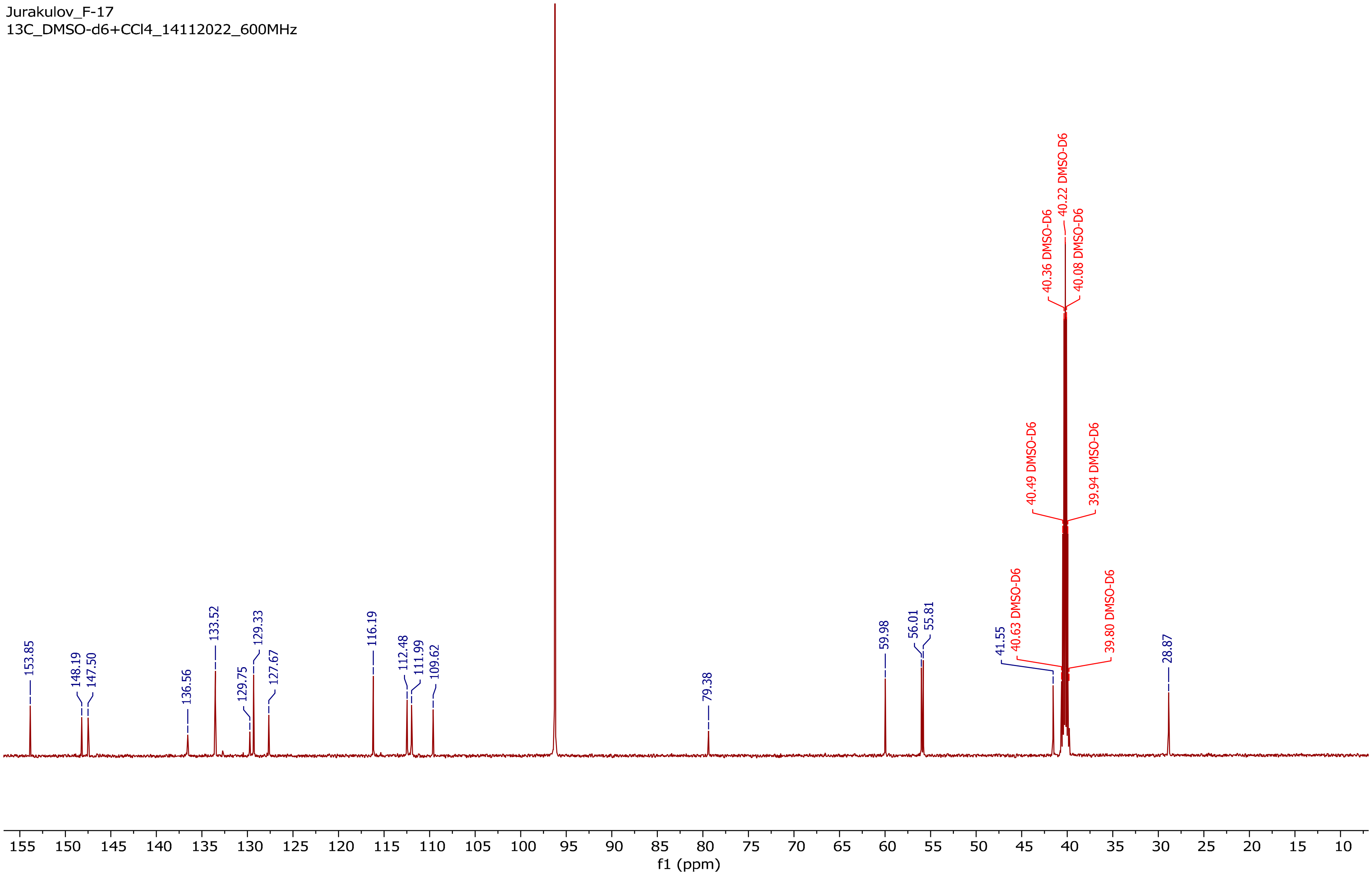

Figure S18. ^{13}C NMR spectrum of compound **3e**

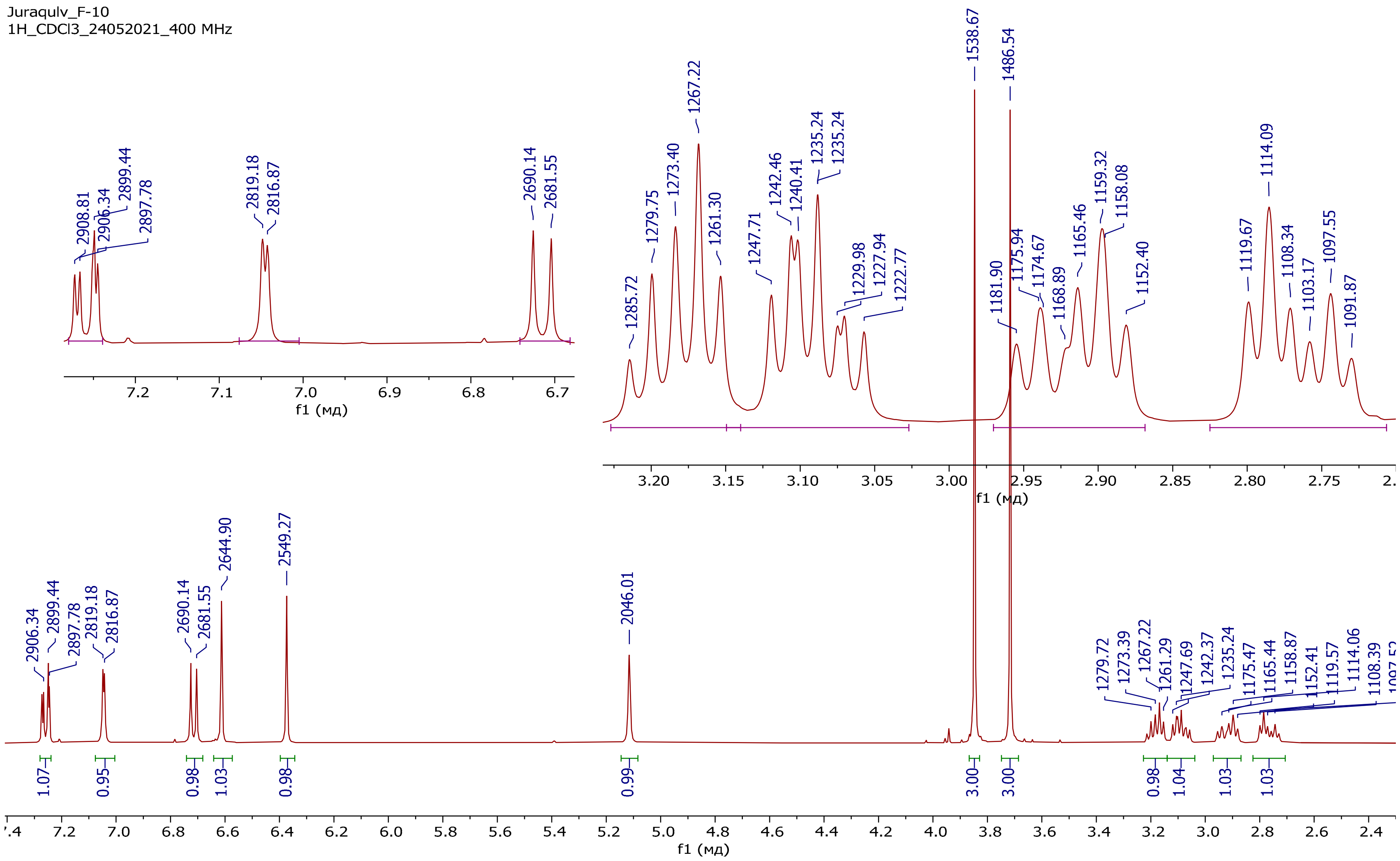

Figure S19. ^{1}H NMR spectrum of compound **3f**.

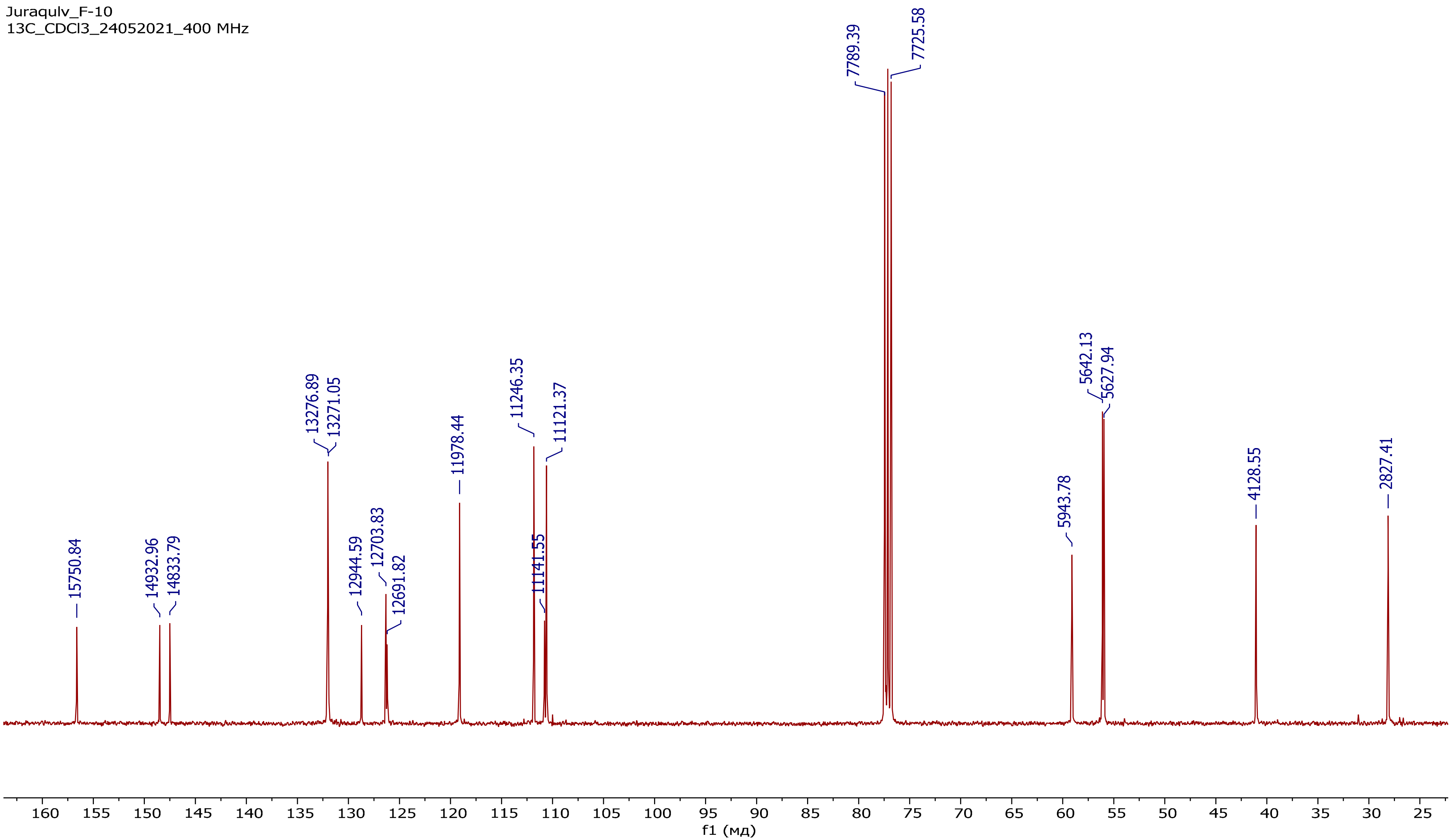

Figure S20. ^{13}C NMR spectrum of compound **3f**.

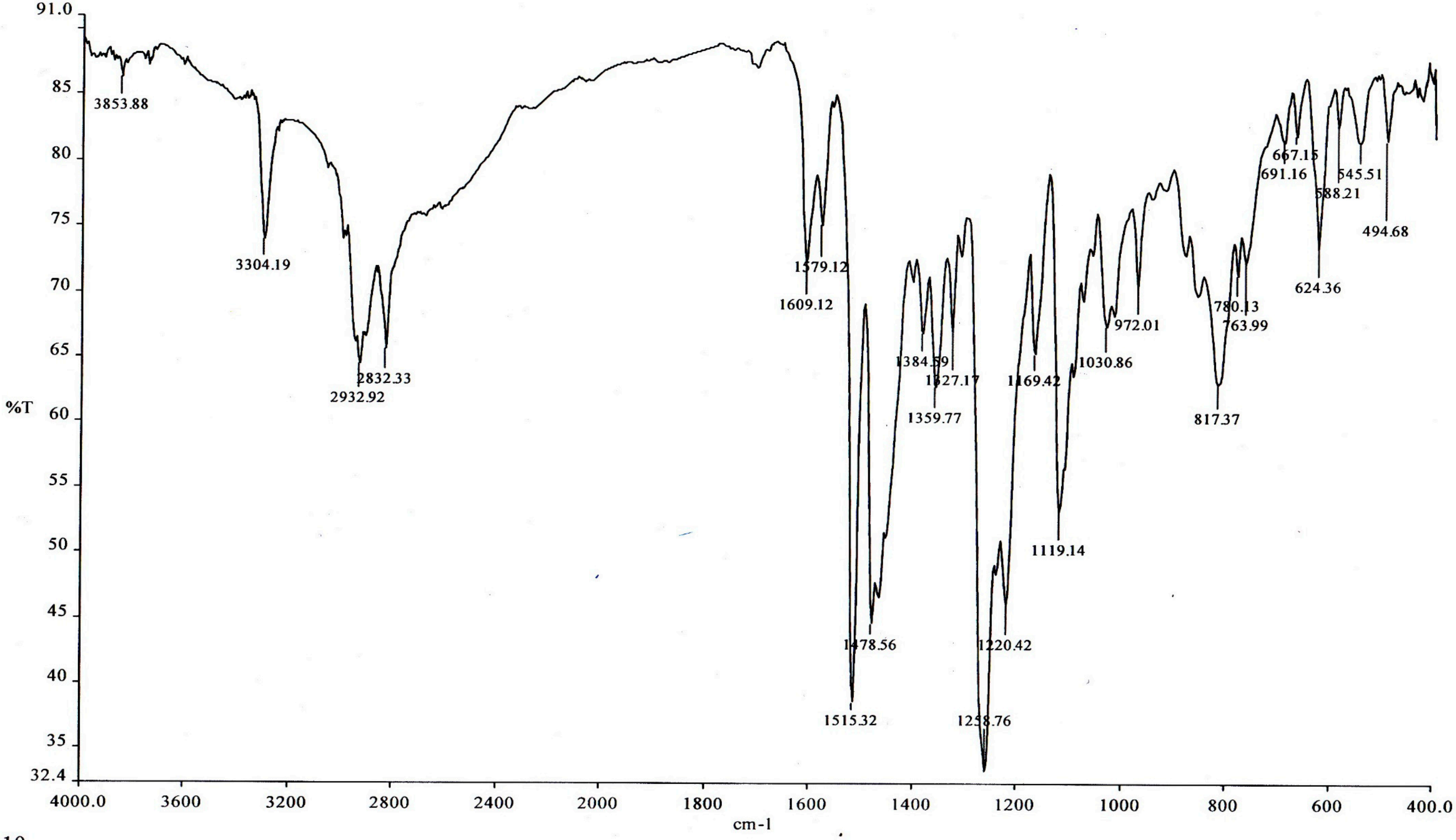

F-10

Figure S21. IR spectrum of compound **3f**.

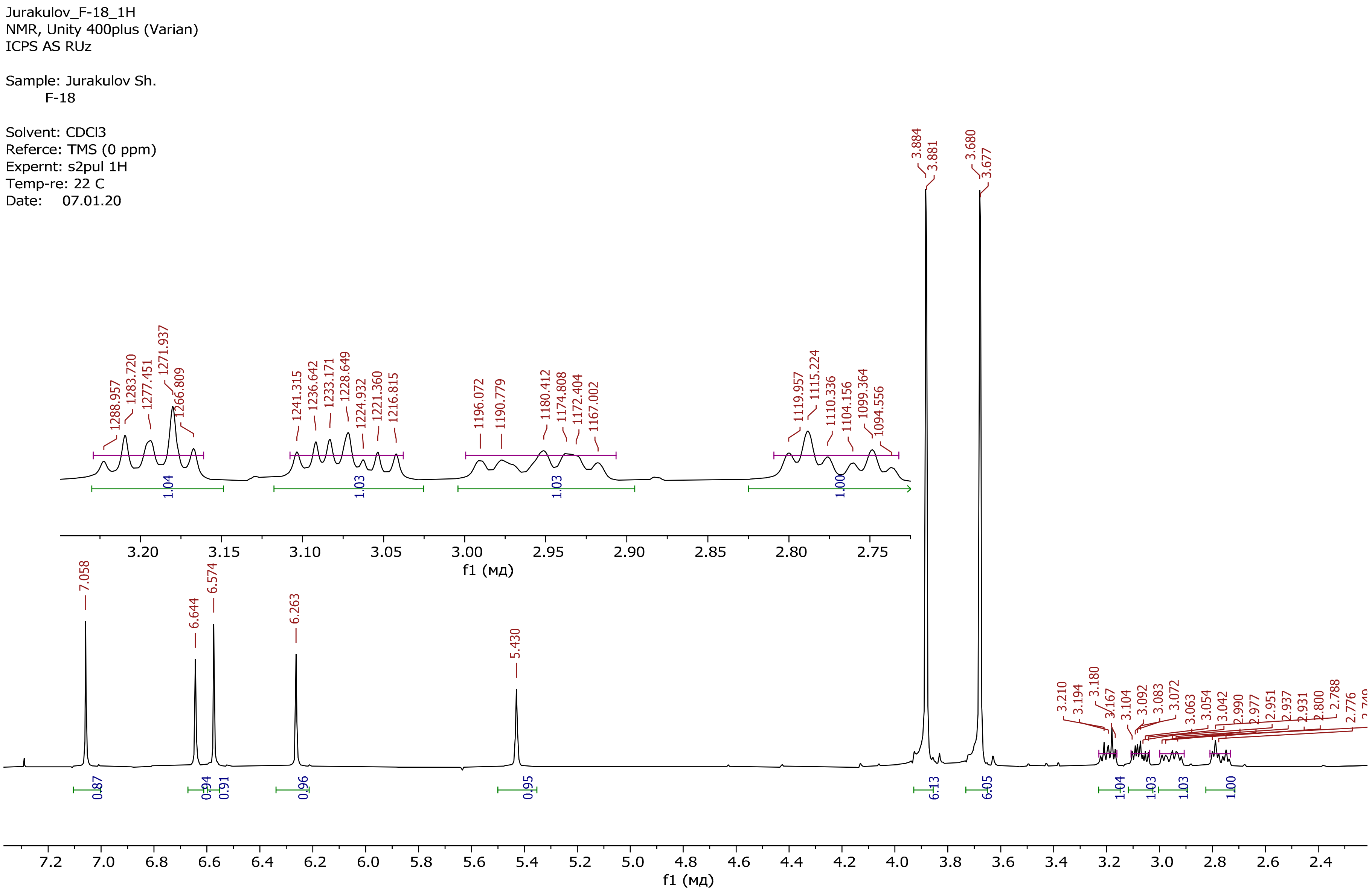

Figure S22. ^{1}H NMR spectrum of compound **3g**.

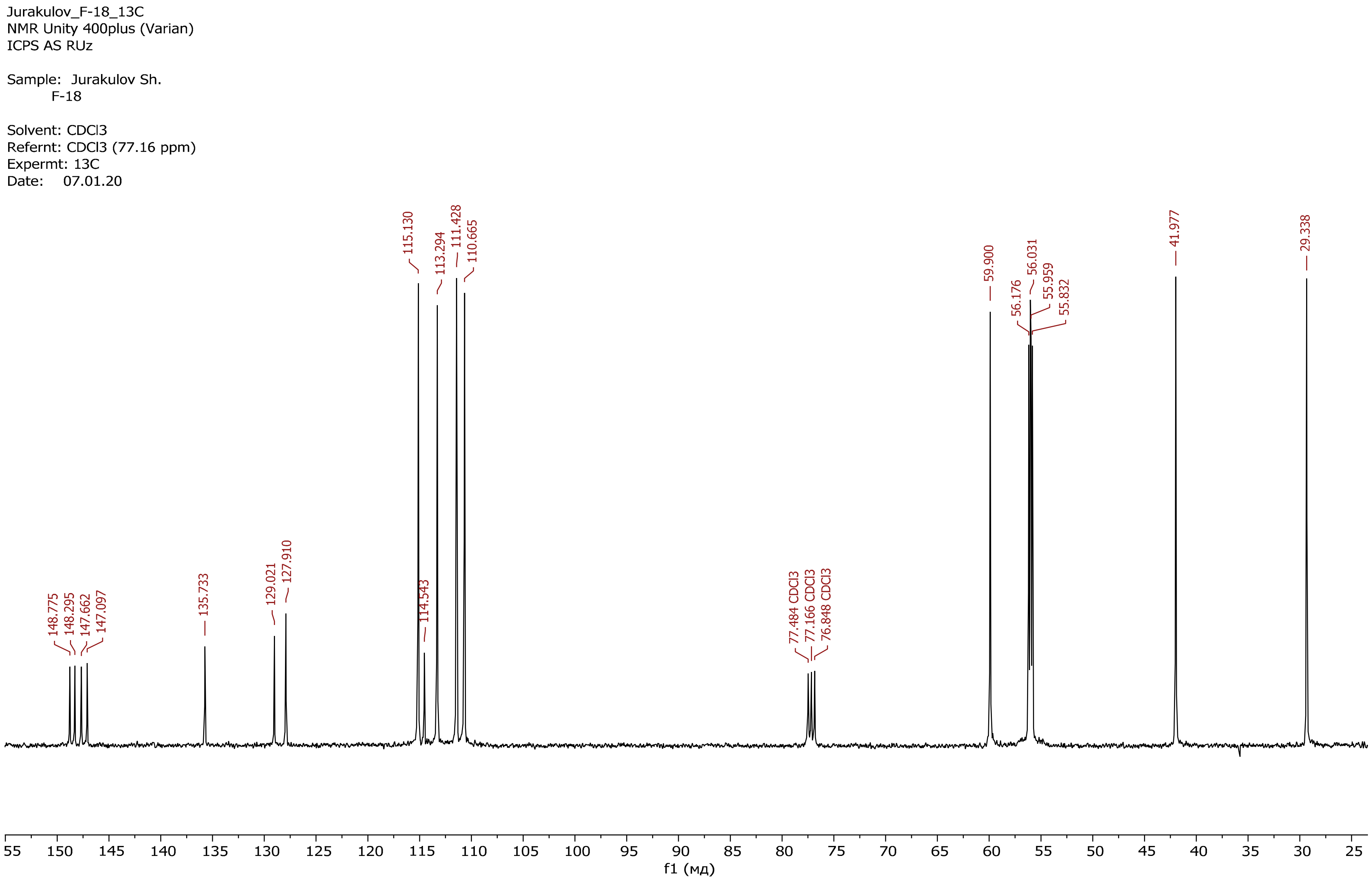

Figure S23. ^{13}C NMR spectrum of compound **3g**.

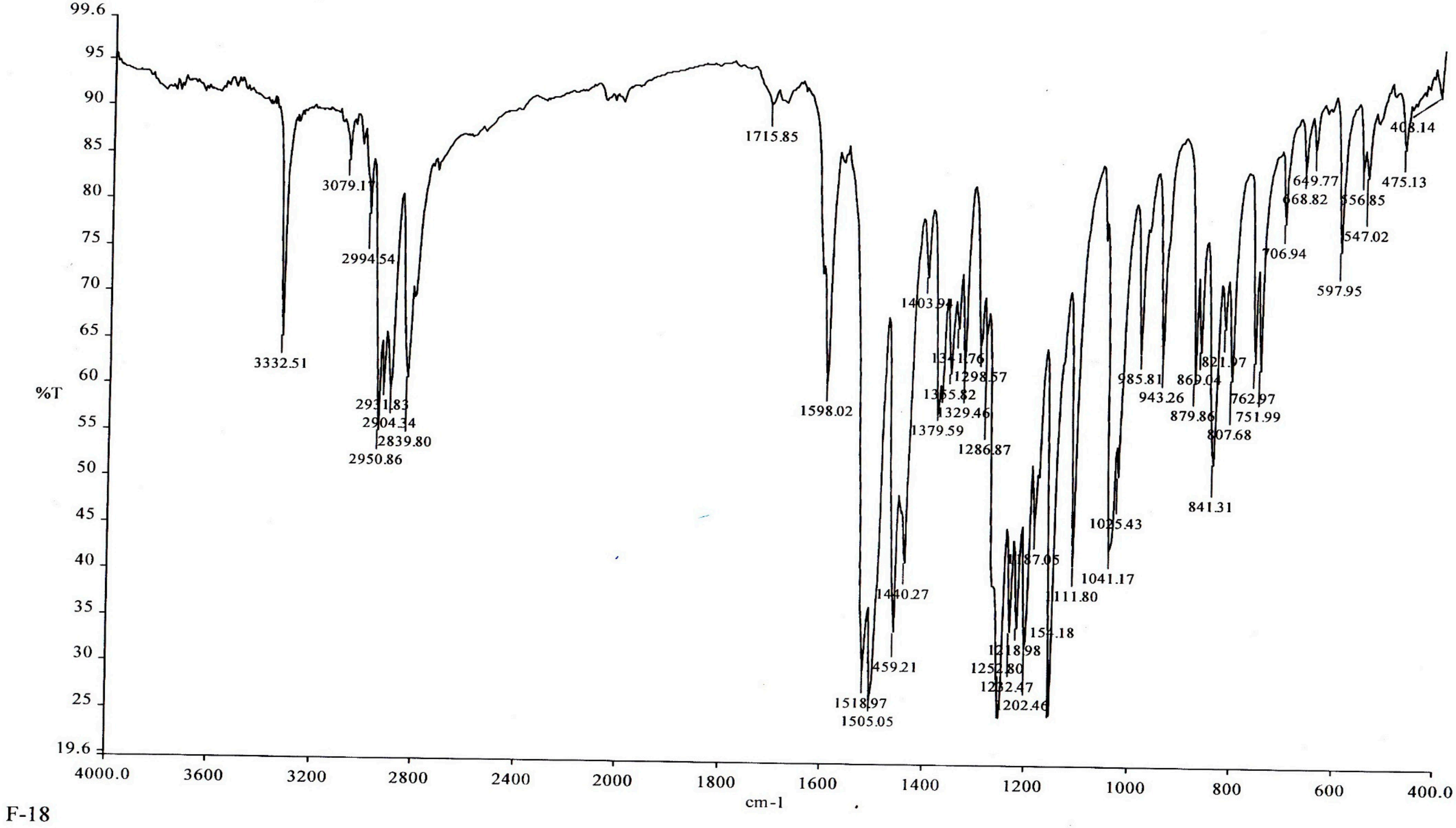

Figure S24. IR spectrum of compound **3g**.

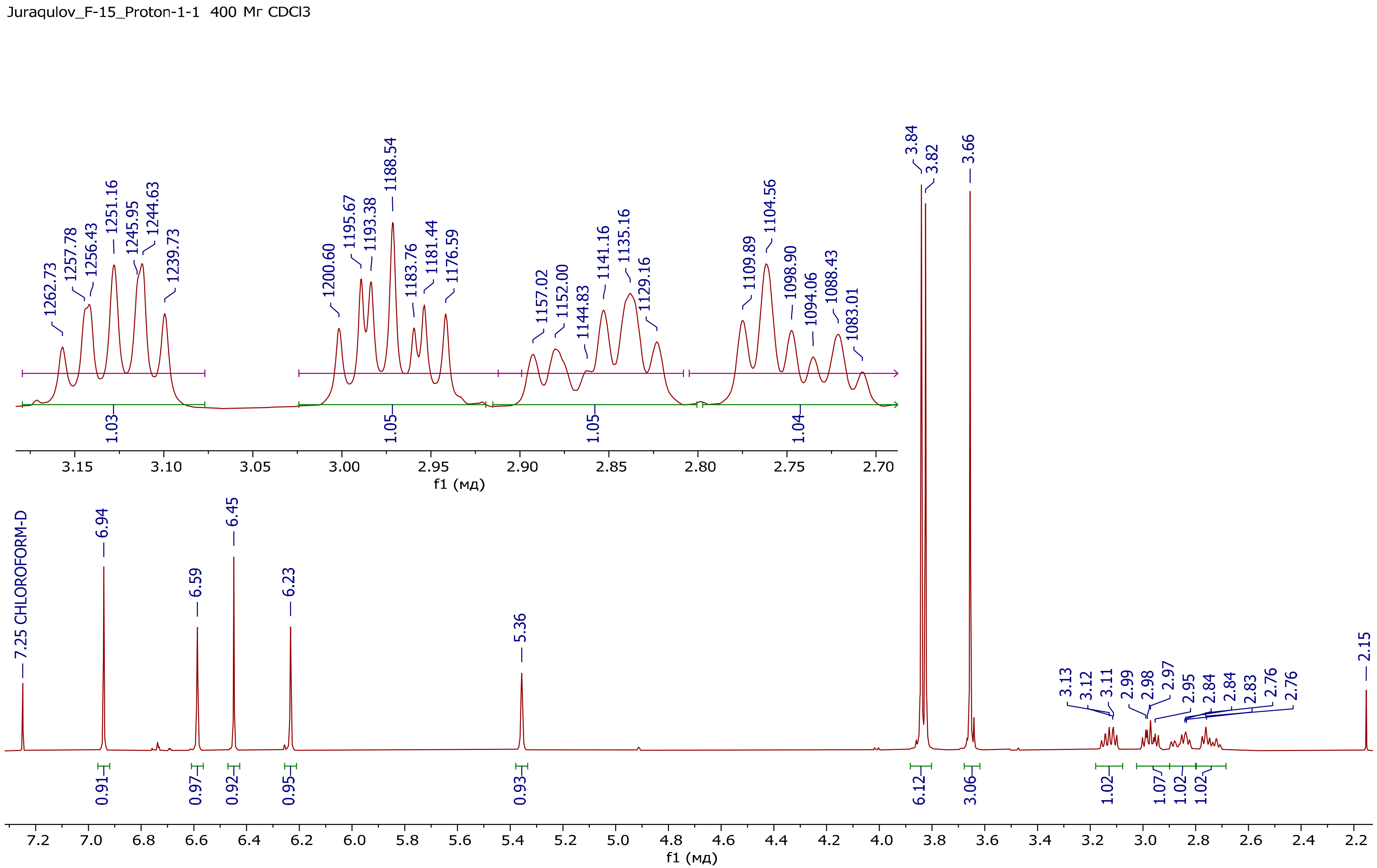

Figure S25. ^{1}H NMR spectrum of compound **3h**.

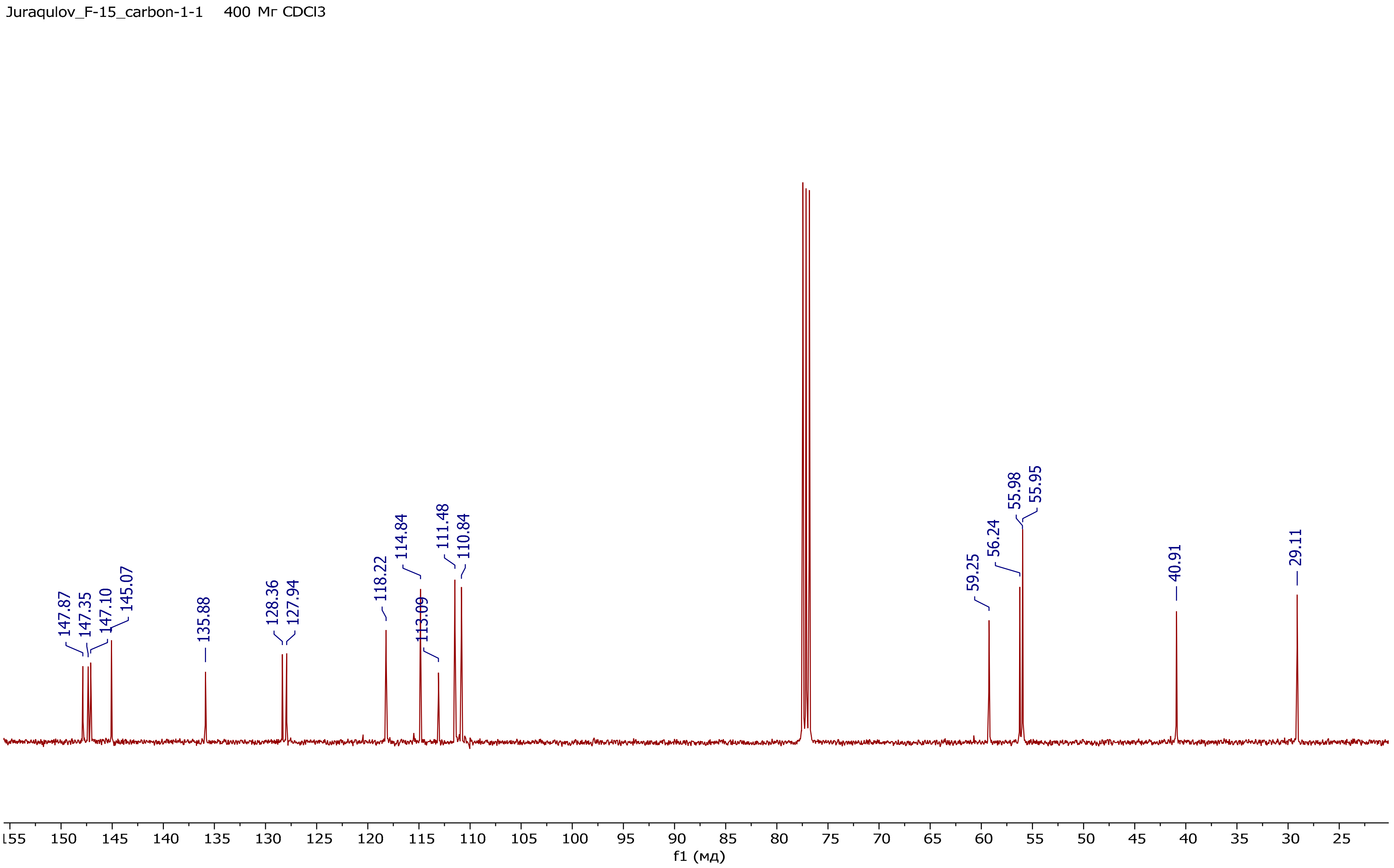

Figure S26. ^{13}C NMR spectrum of compound **3h**.

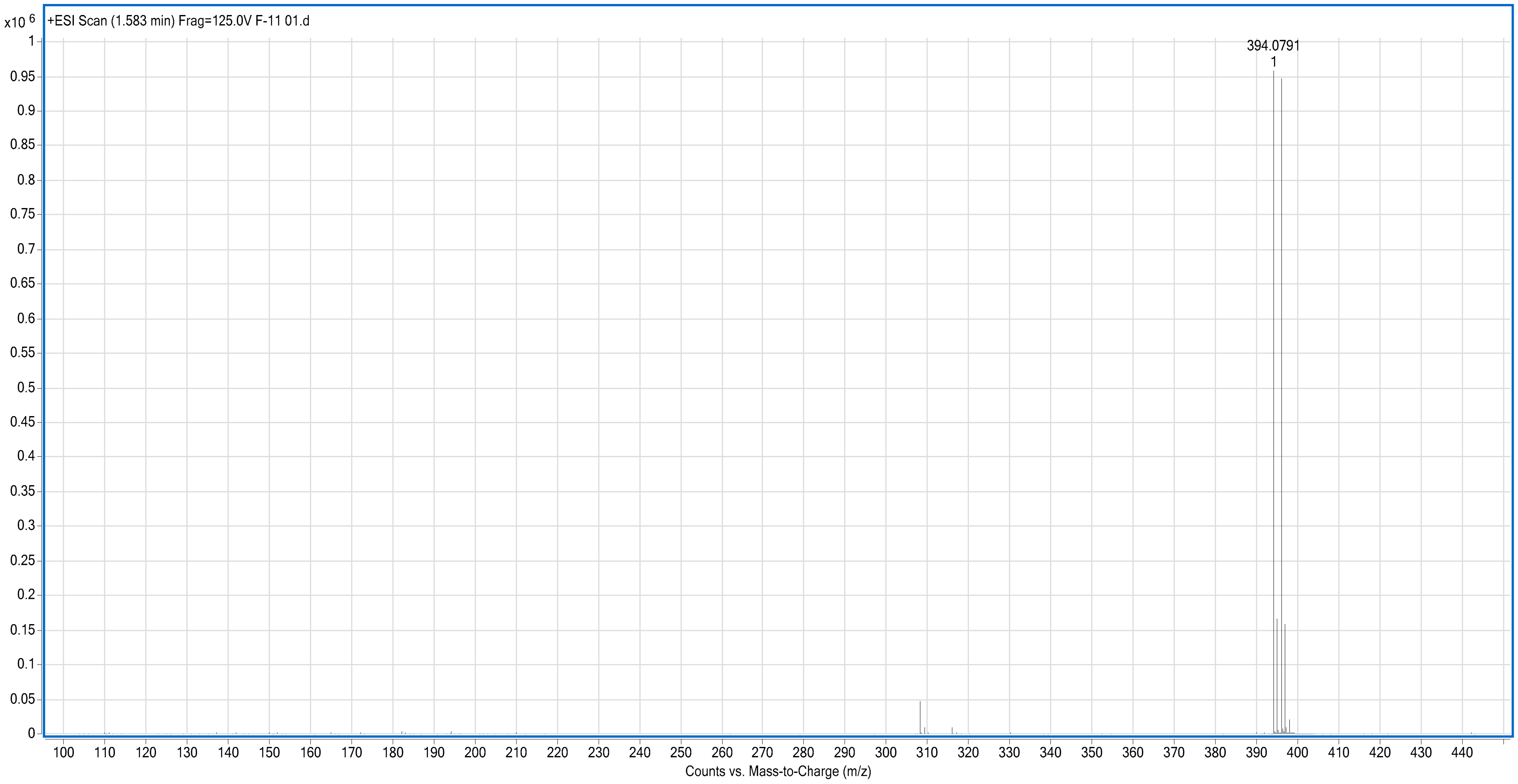

Figure S27. +ESI-mass- spectrum of compound **3h** *(m/z* 394 $(M+H)^+$ from the isotope bromine 79).

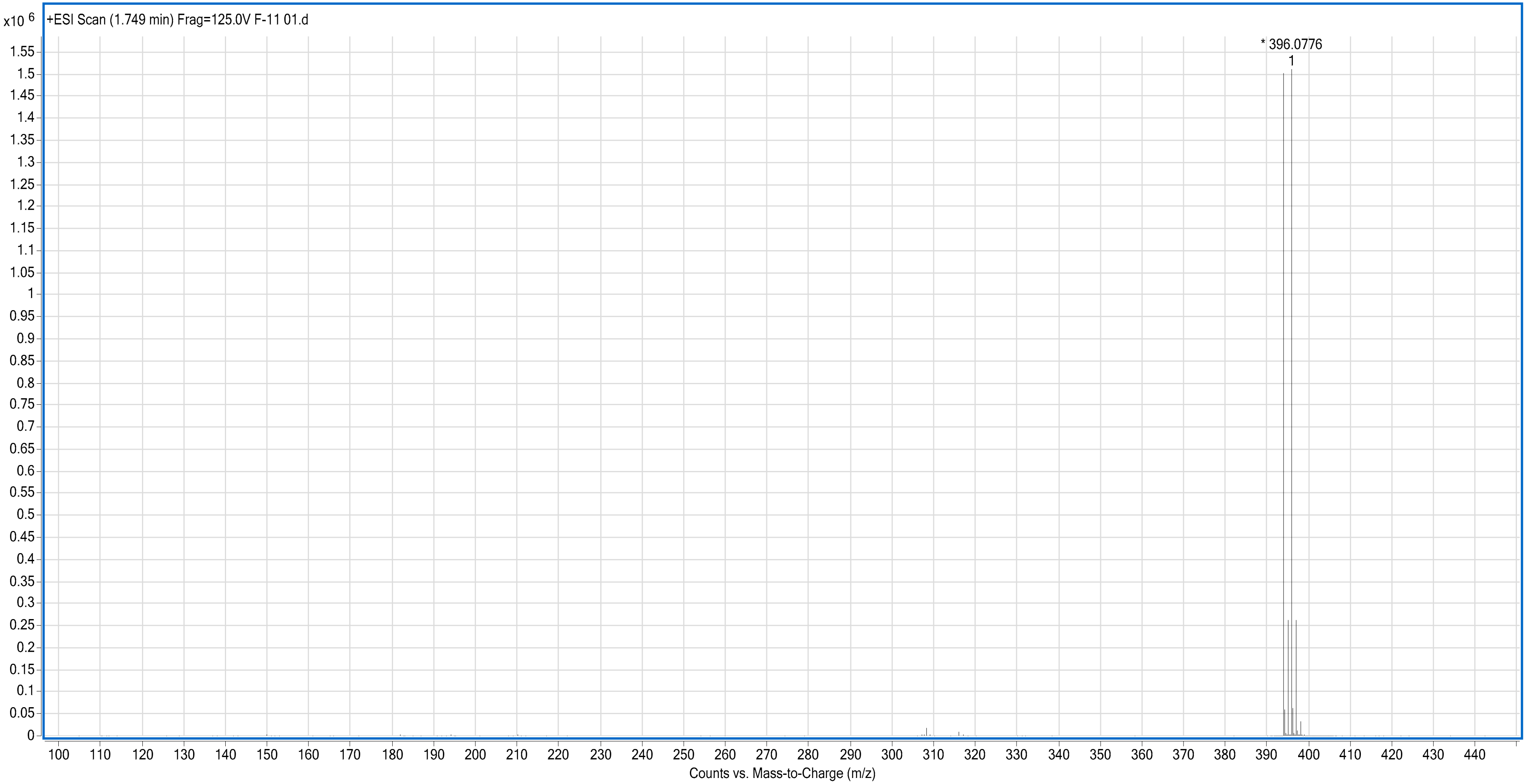

Figure S28. +ESI-mass- spectrum of compound **3h** *(m/z* 396 $(M+H)^{+}$ from the isotope bromine 81).

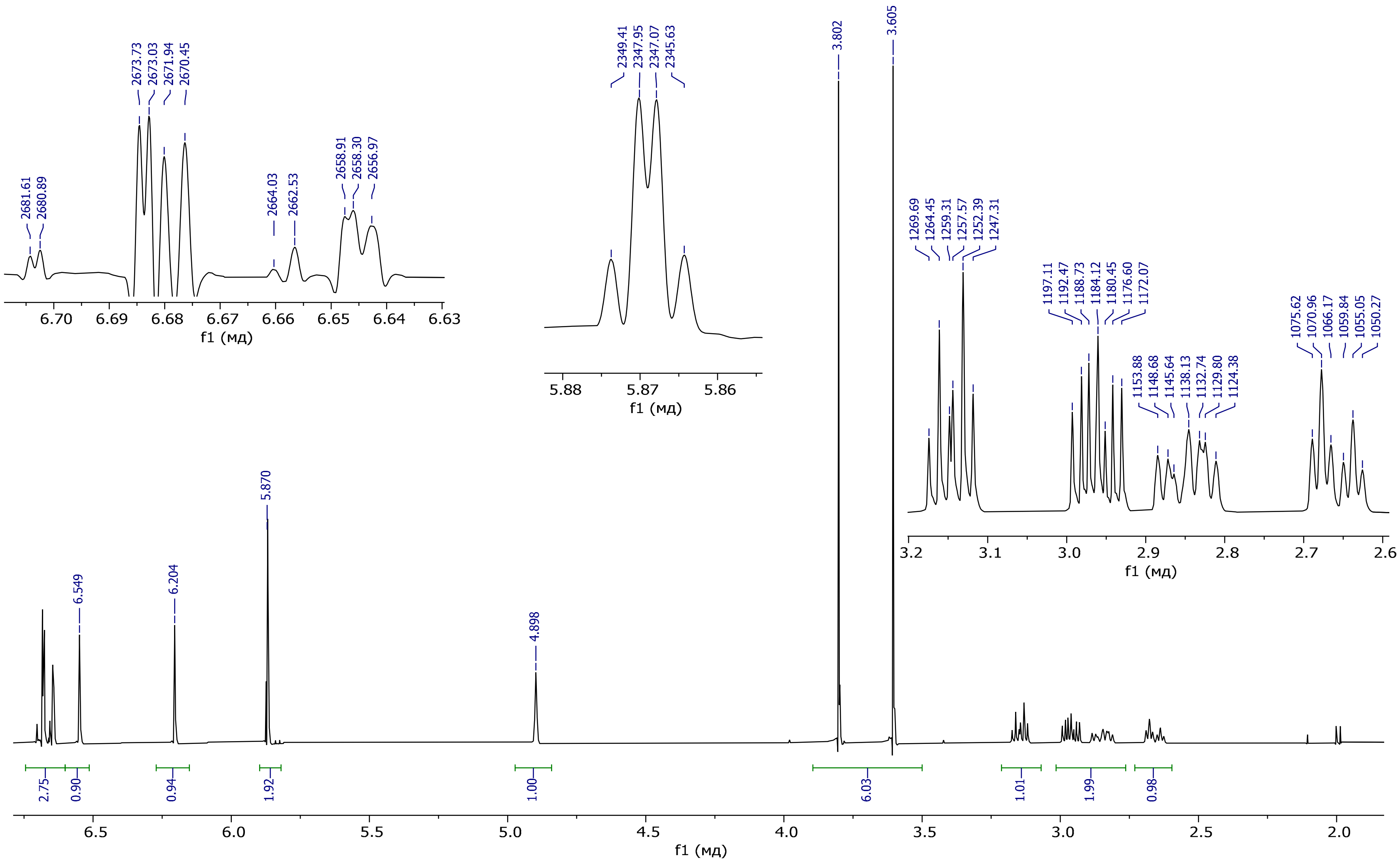

Figure S29. ^{1}H NMR spectrum of compound **3i**.

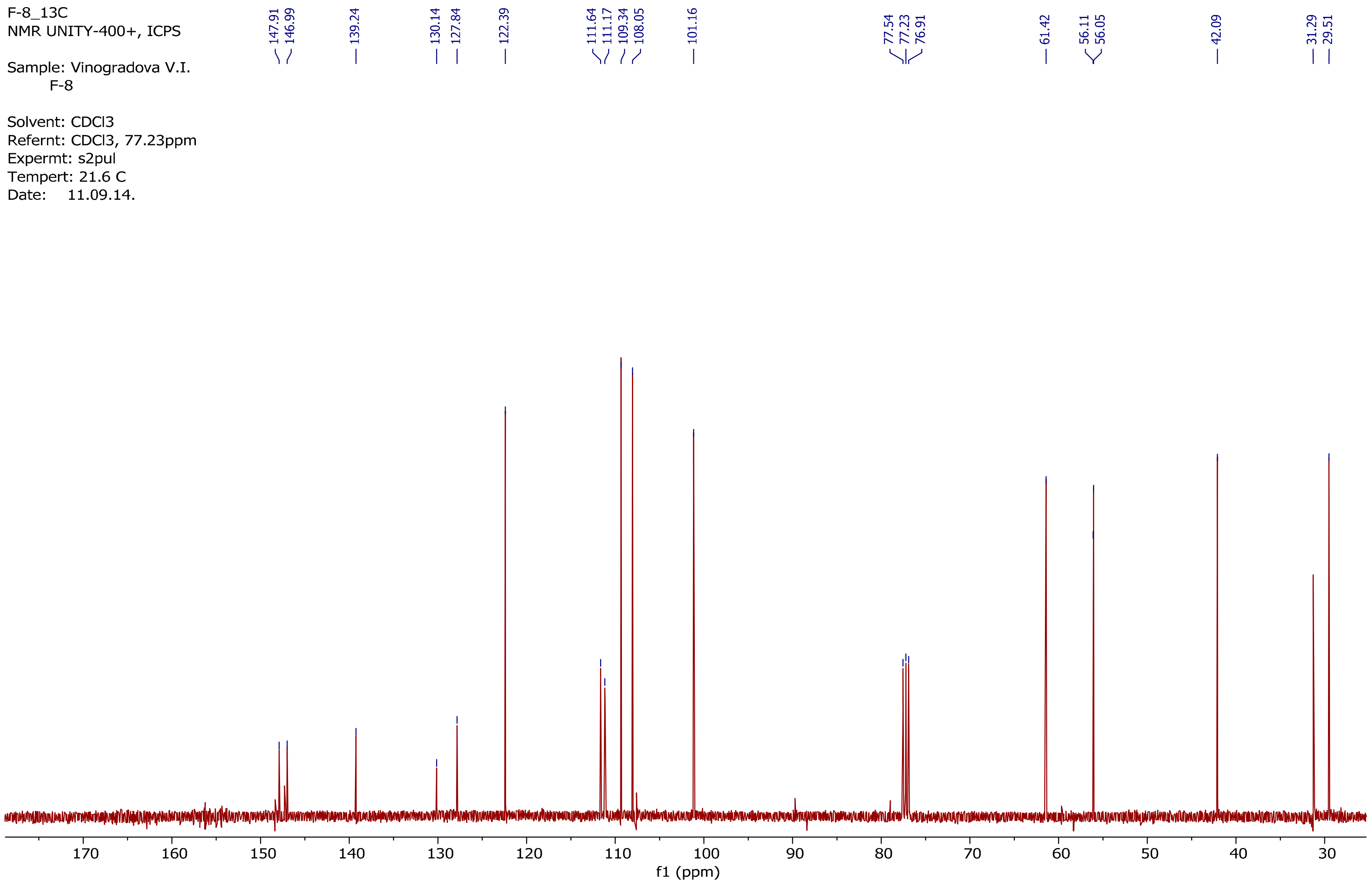

Figure S30. ^{13}C NMR spectrum of compound **3i**.

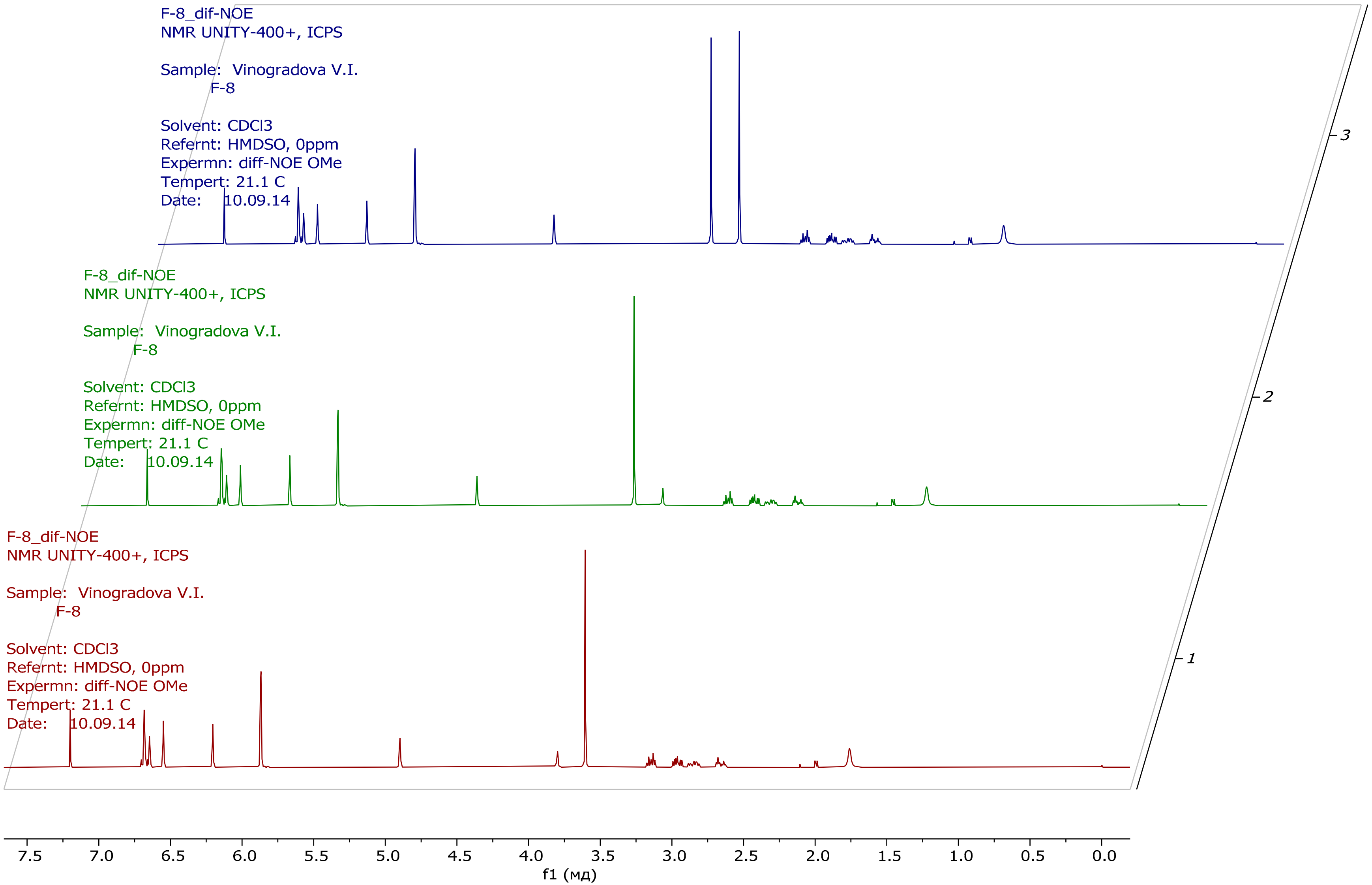

Figure S31. Diff-NOE spectrum of compound **3i.**

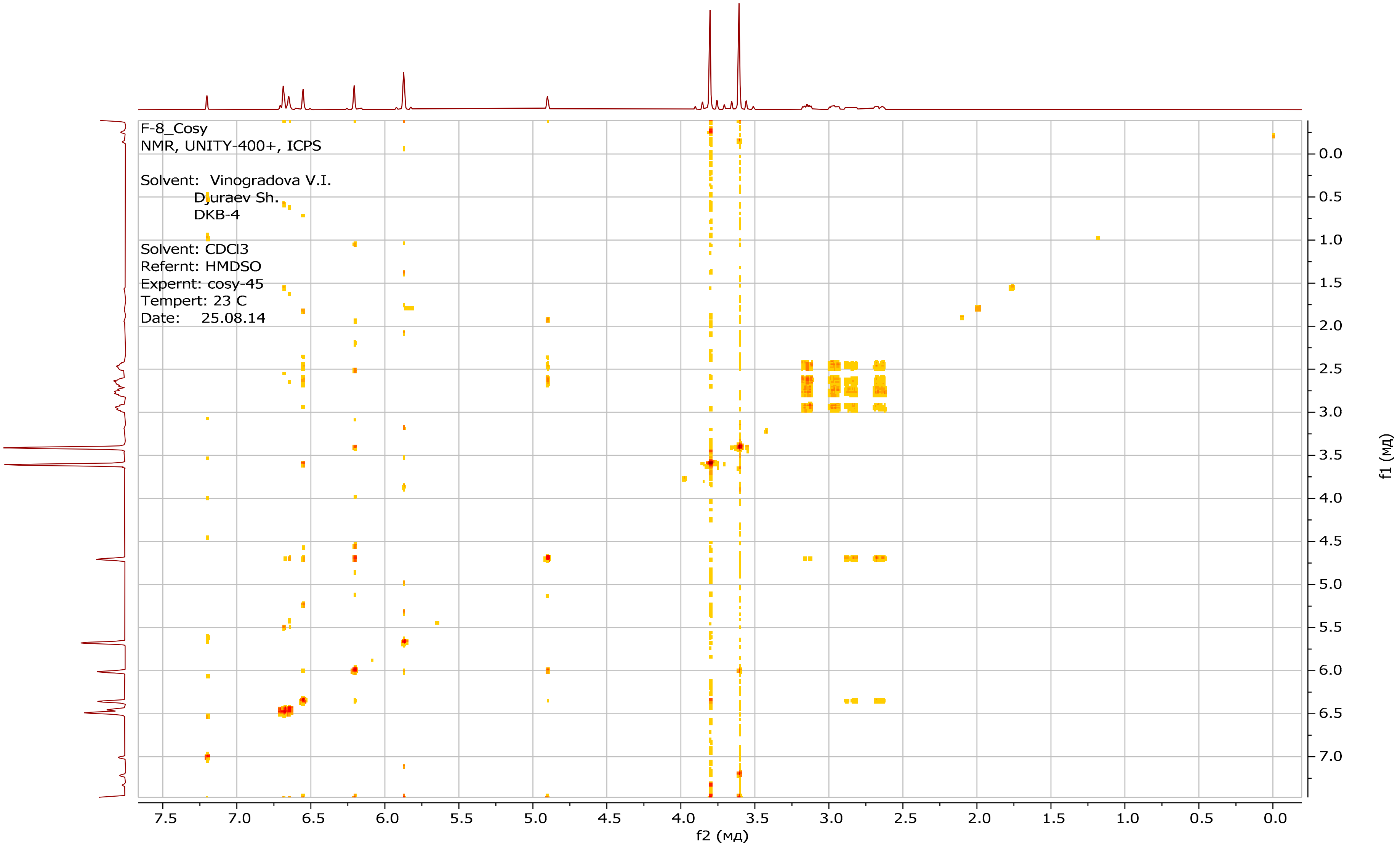

Figure S32. COSY spectrum of compound **3i.**

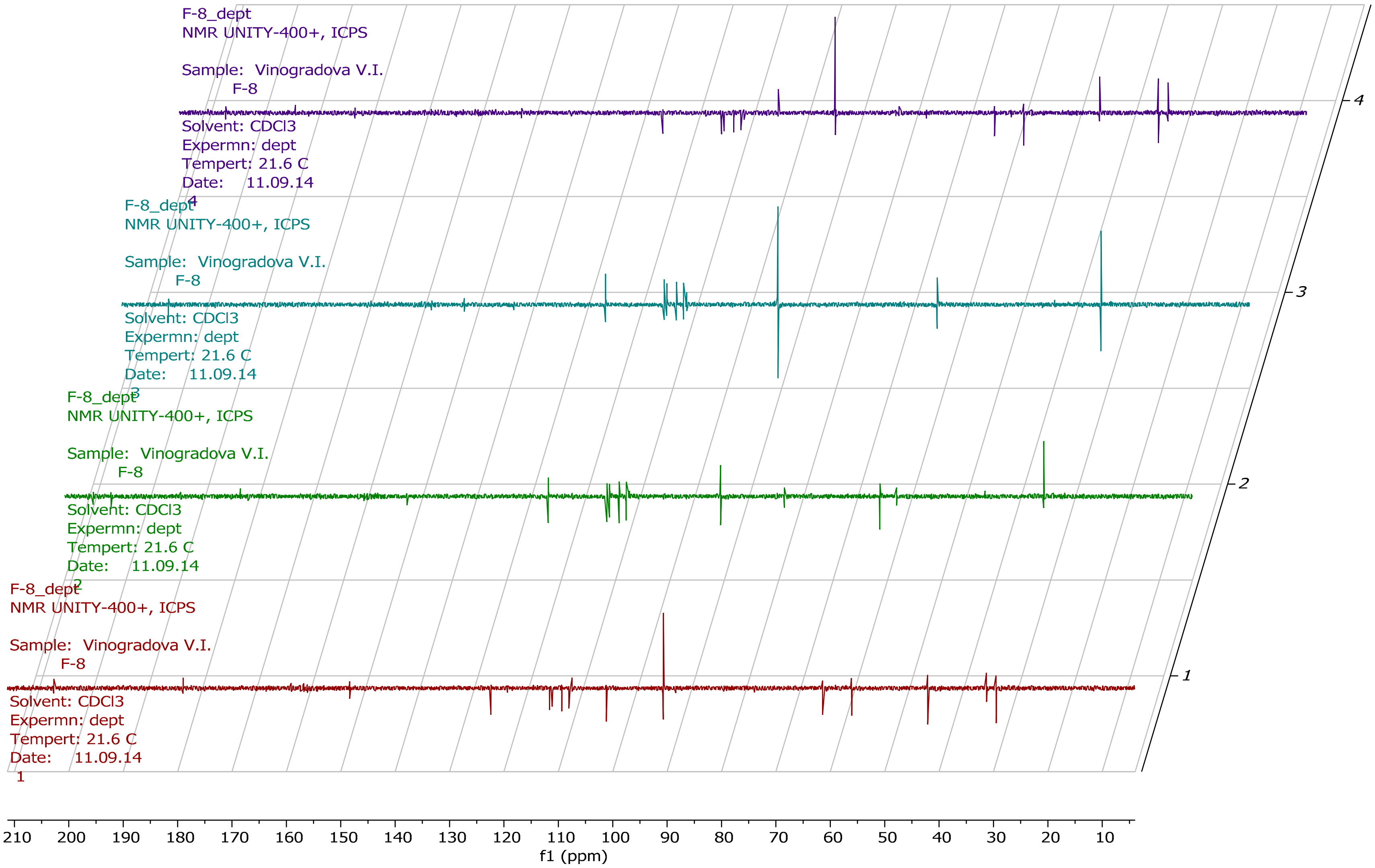

Figure S33. Dept spectrum of compound **3i.**

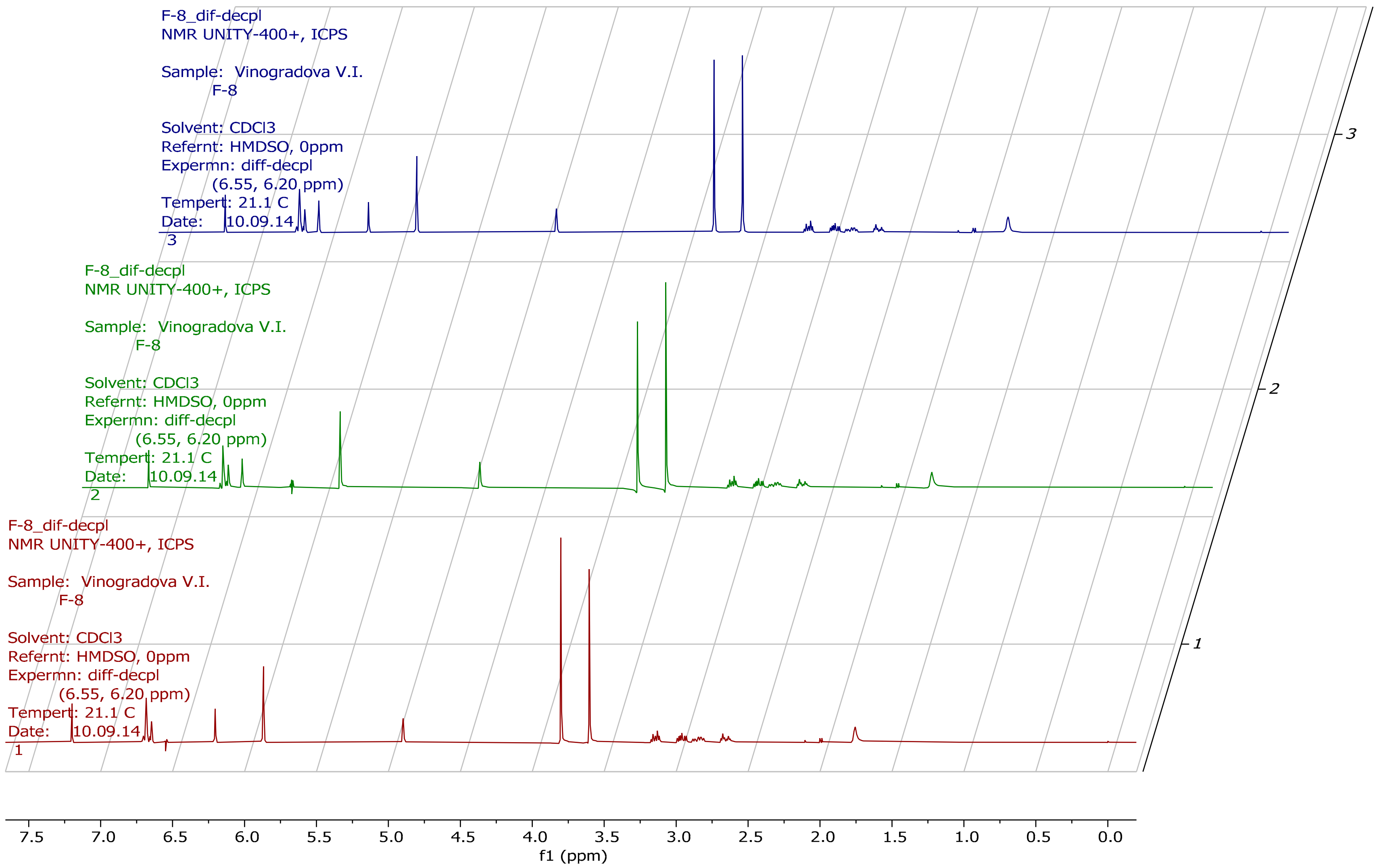

Figure S34. Diff-decpl spectrum of compound **3i.**

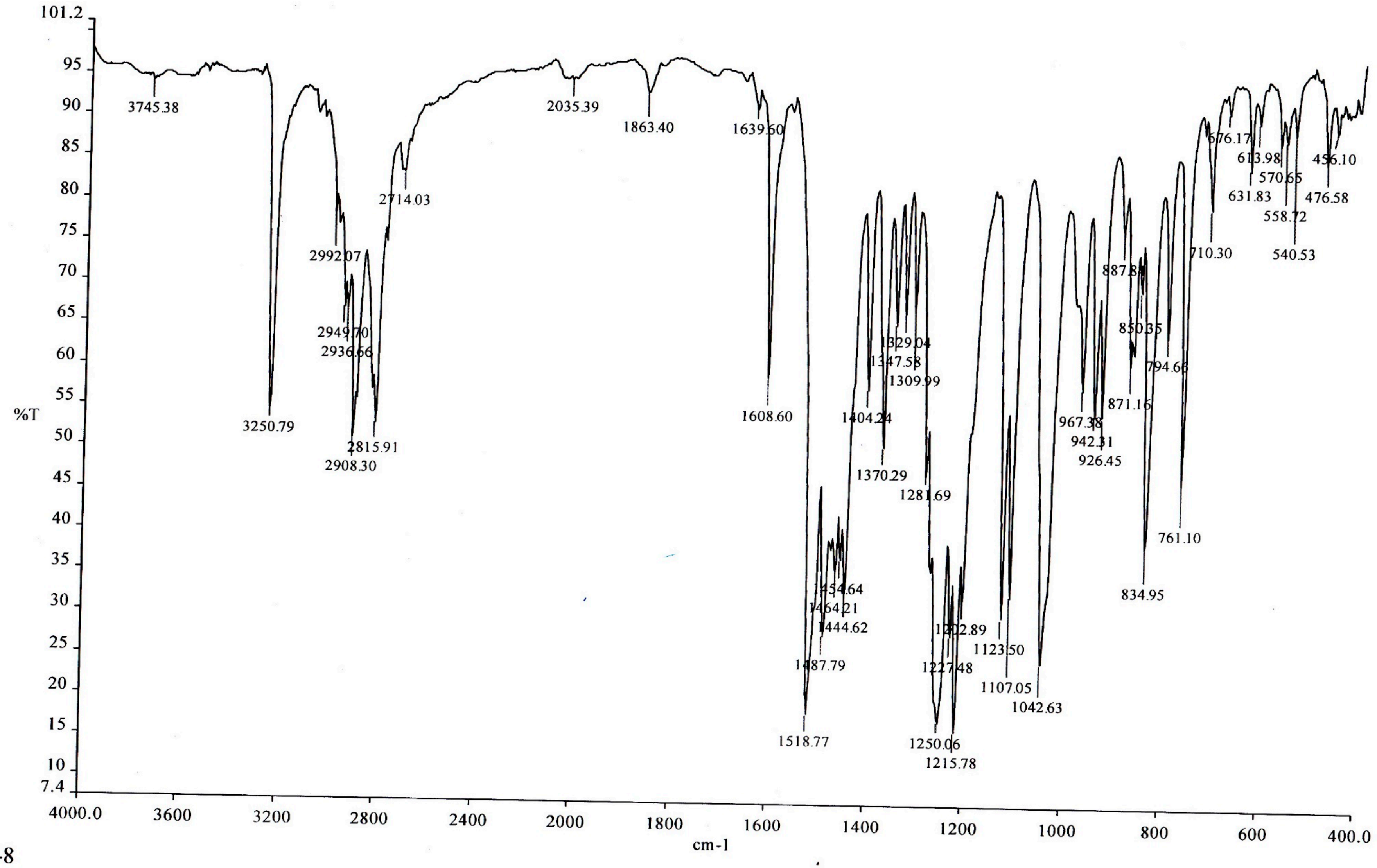

F-8

Figure S35. IR spectrum of compound **3i**.

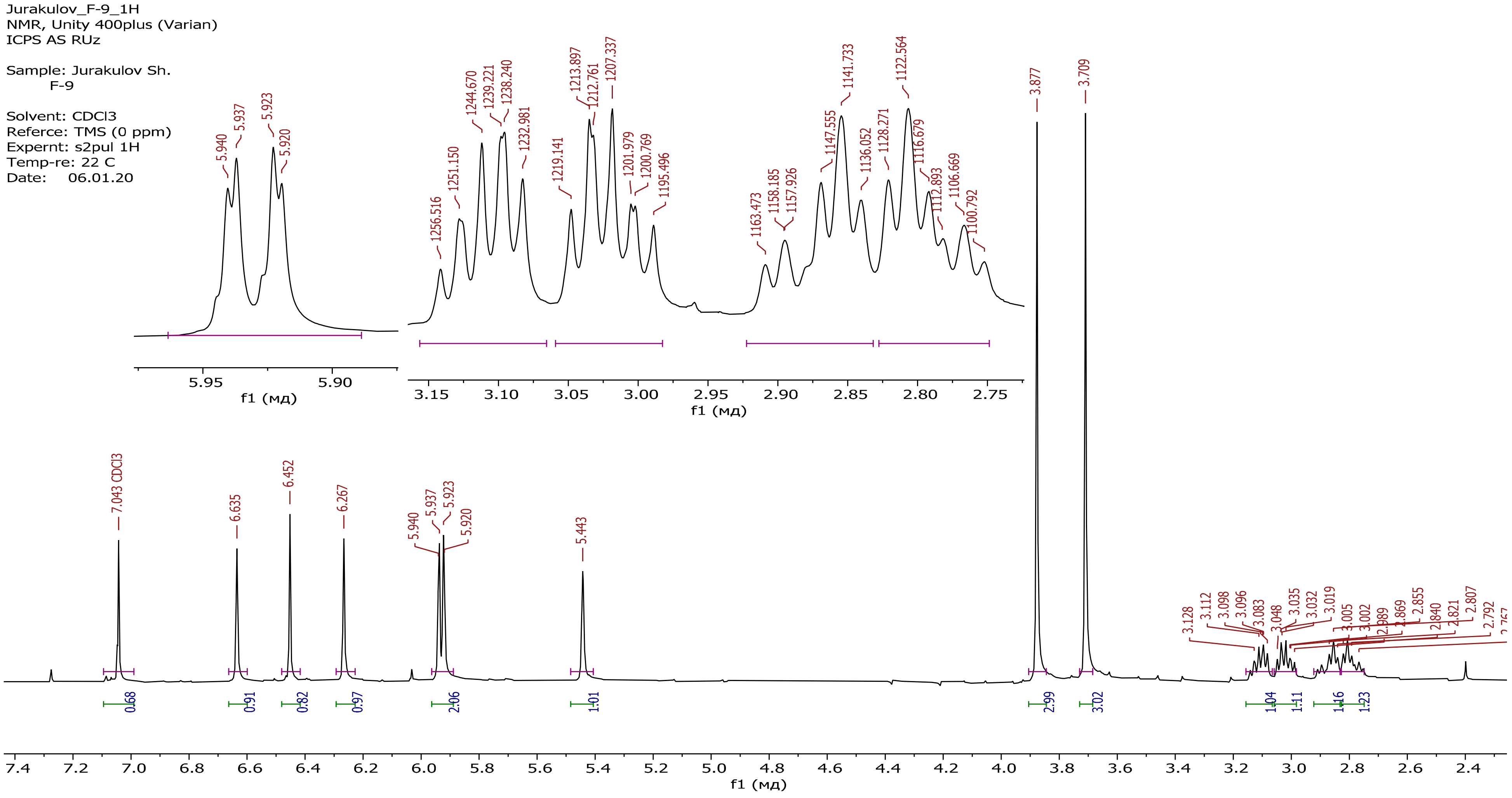

Figure S36. ^{1}H NMR spectrum of compound **3j**.

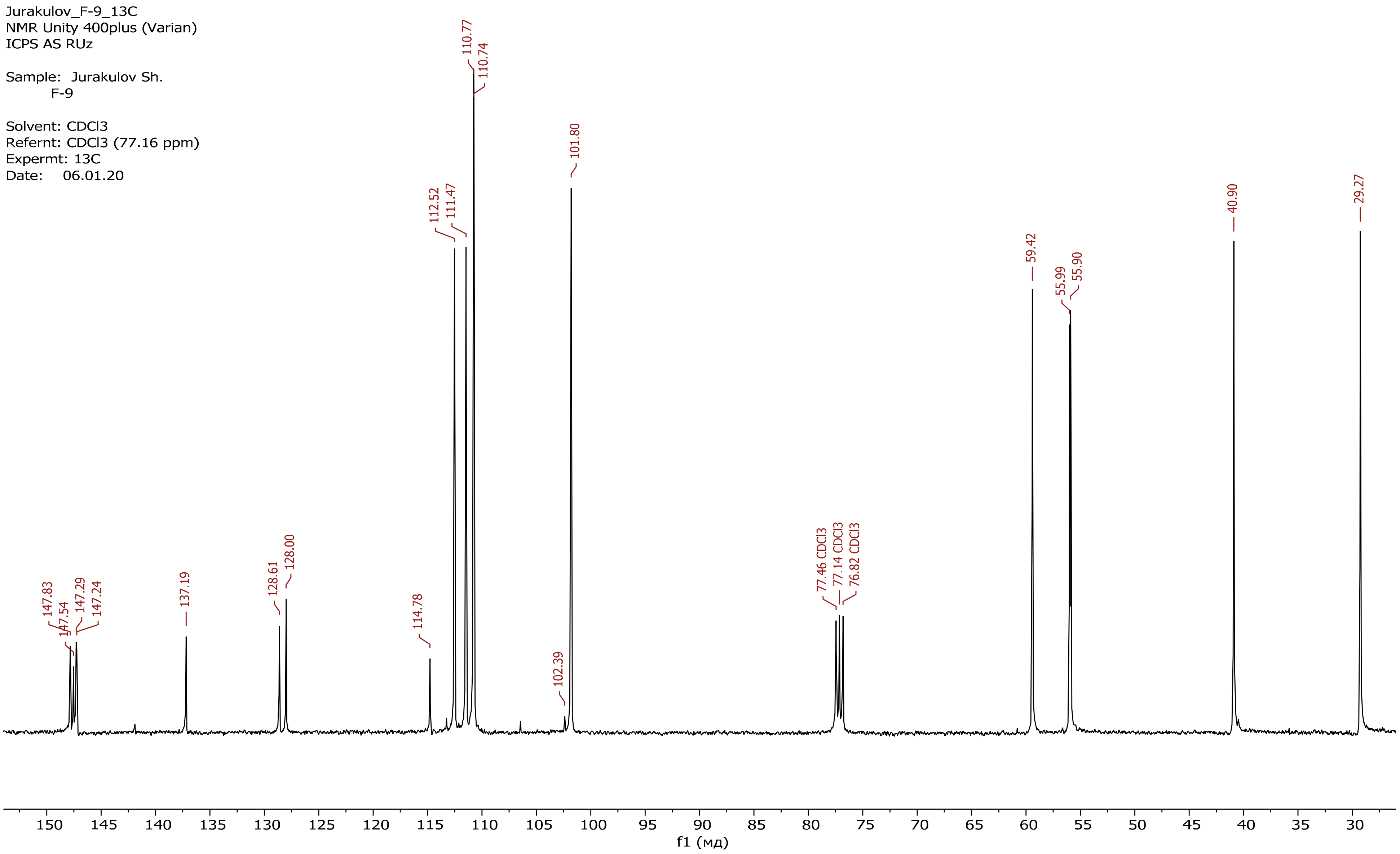

Figure S37. ^{13}C NMR spectrum of compound **3j**.

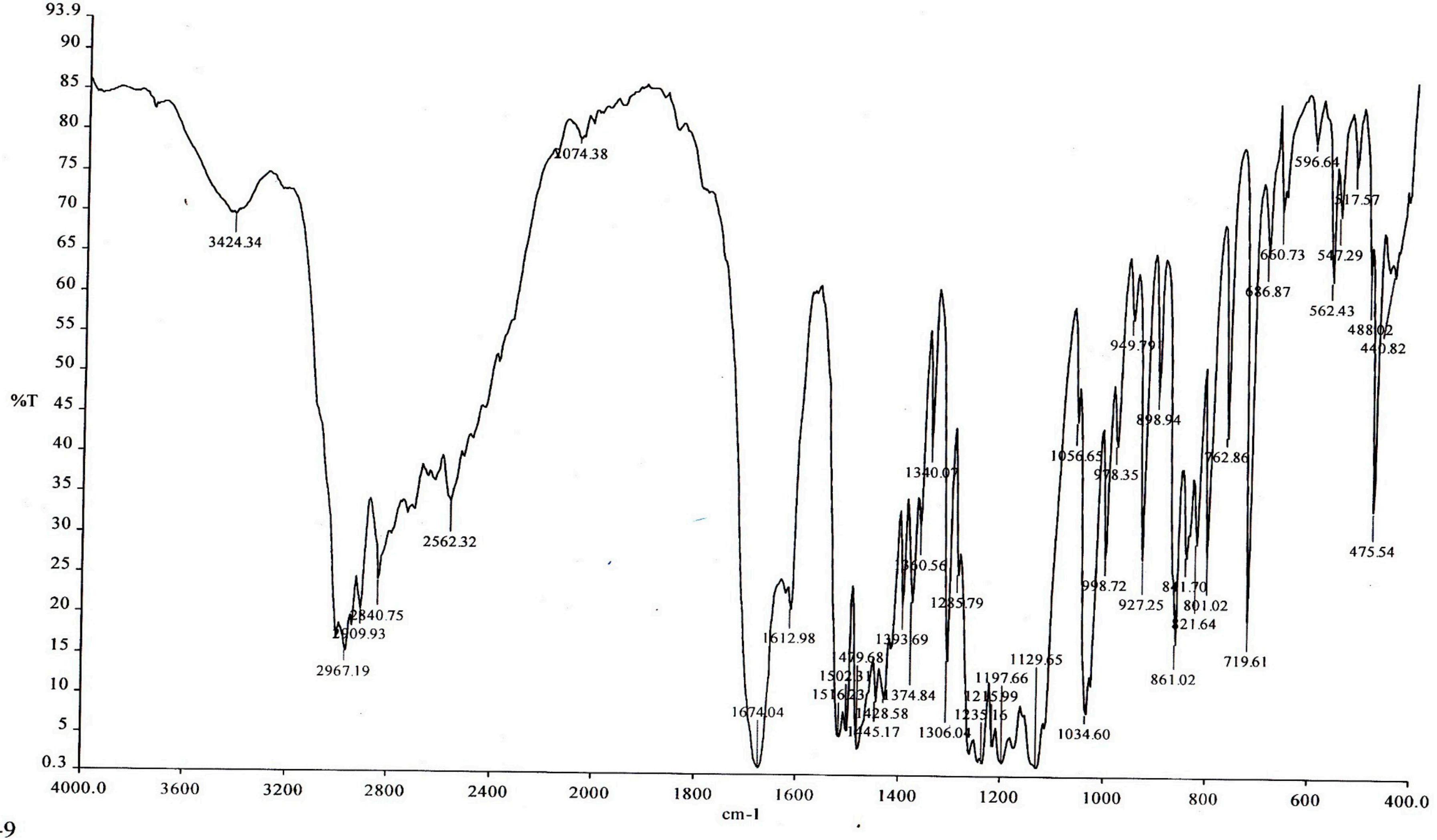

F-9

Figure S38. IR spectrum of compound **3j**.

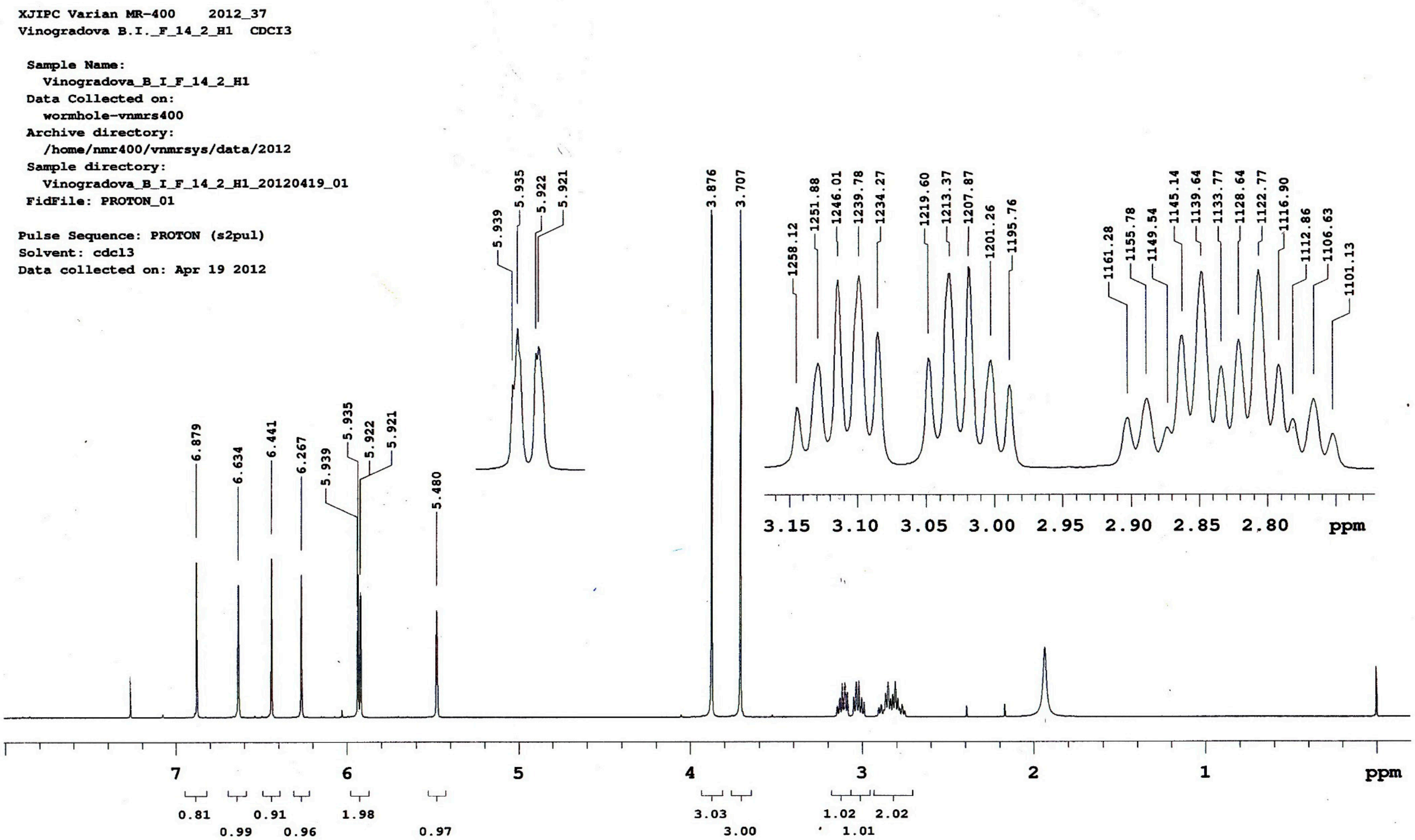

Figure S39. ^{1}H NMR spectrum of compound **3k**.

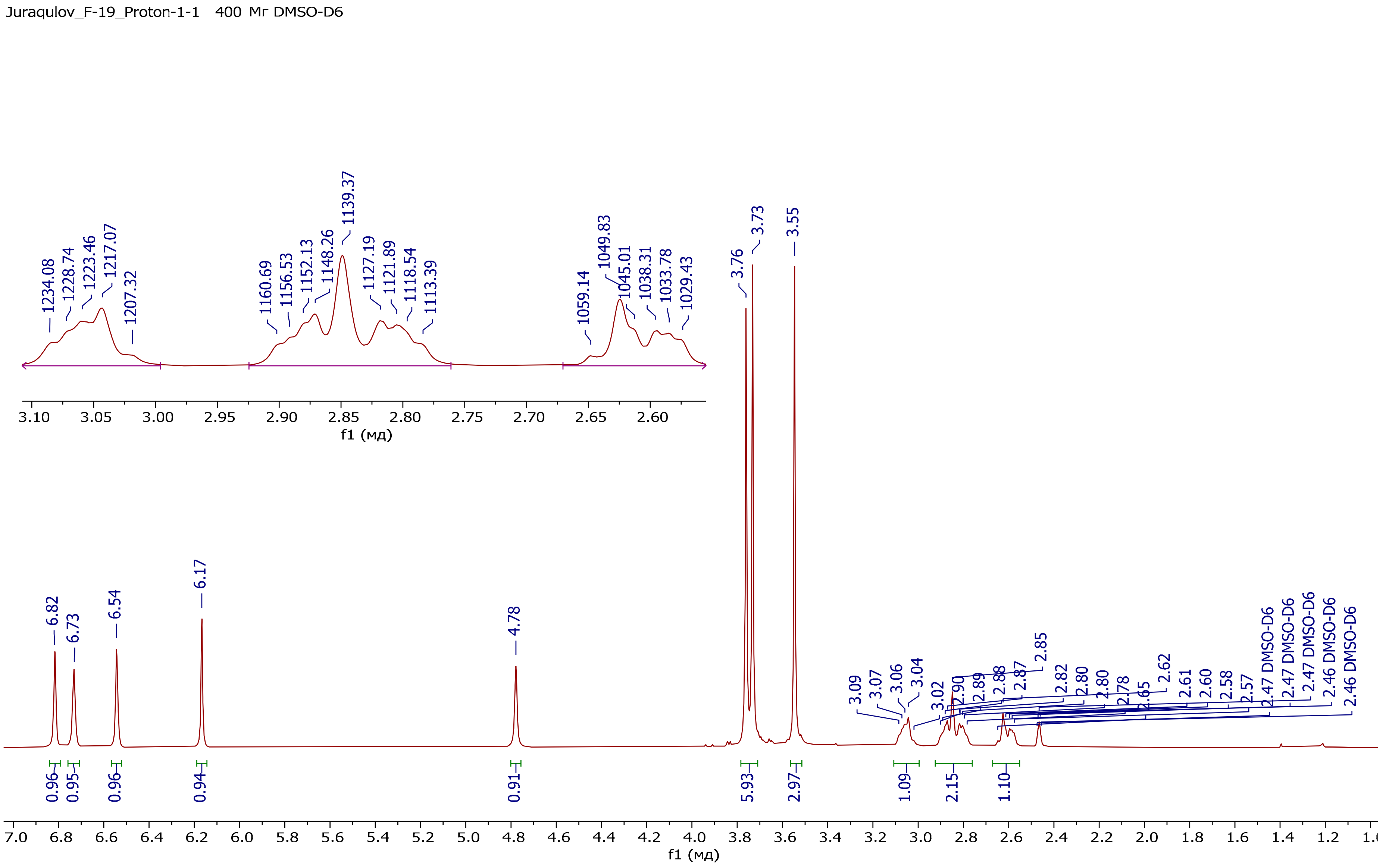

Figure S40. ^{1}H NMR spectrum of compound **3l**.

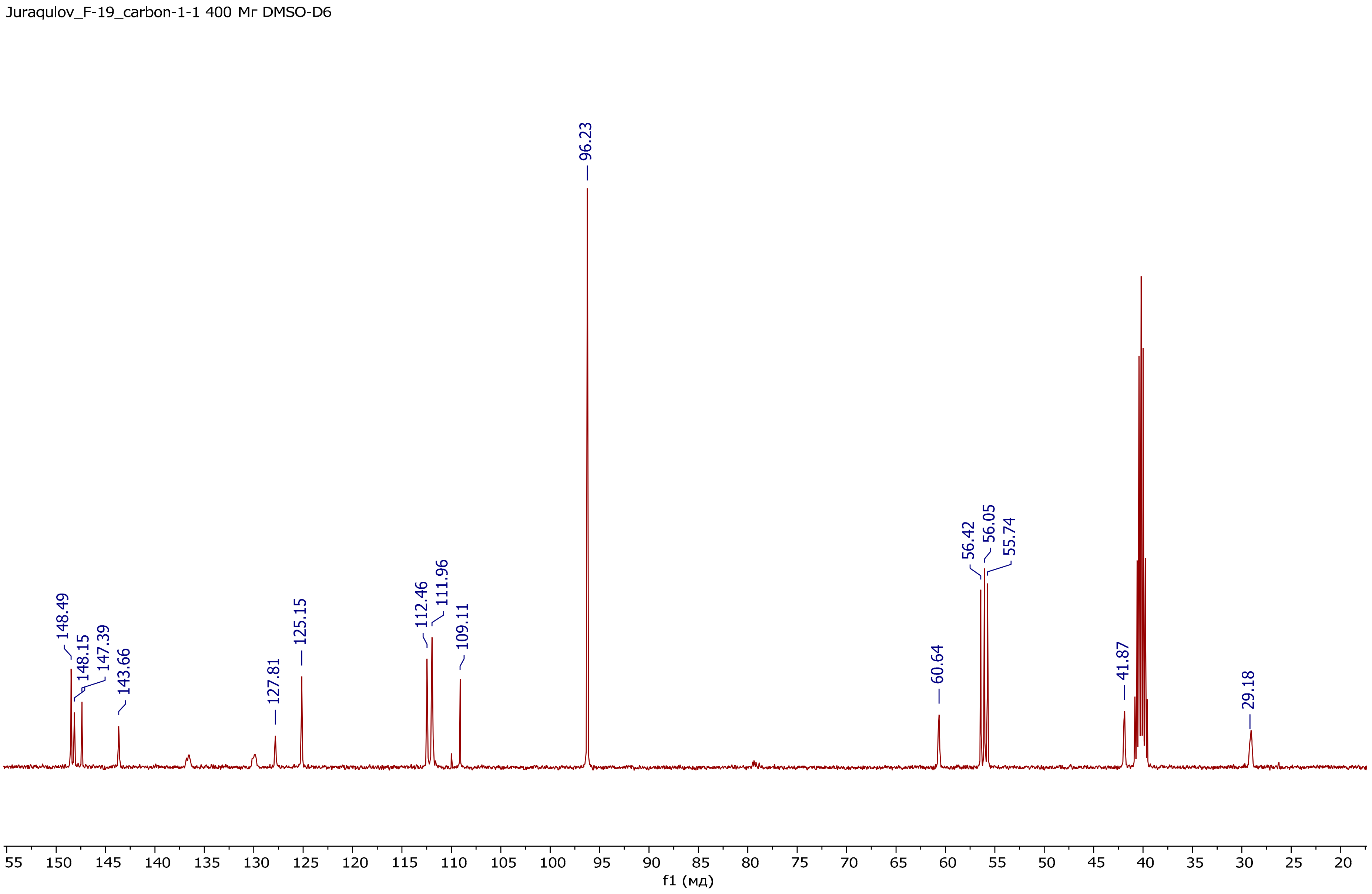

Figure S41. ^{13}C NMR spectrum of compound **3l**.

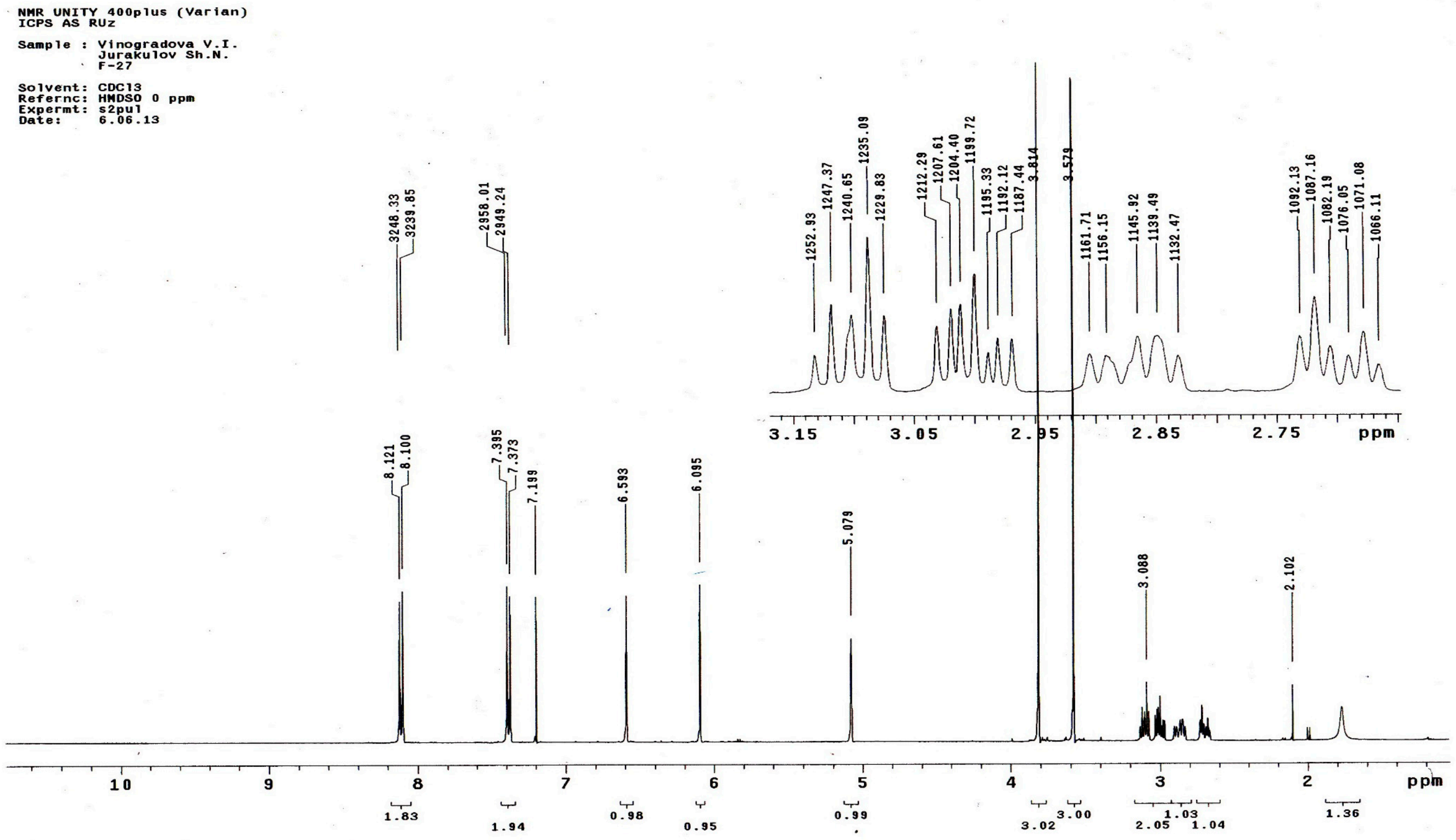

Figure S42. ^{1}H NMR spectrum of compound **3m**.

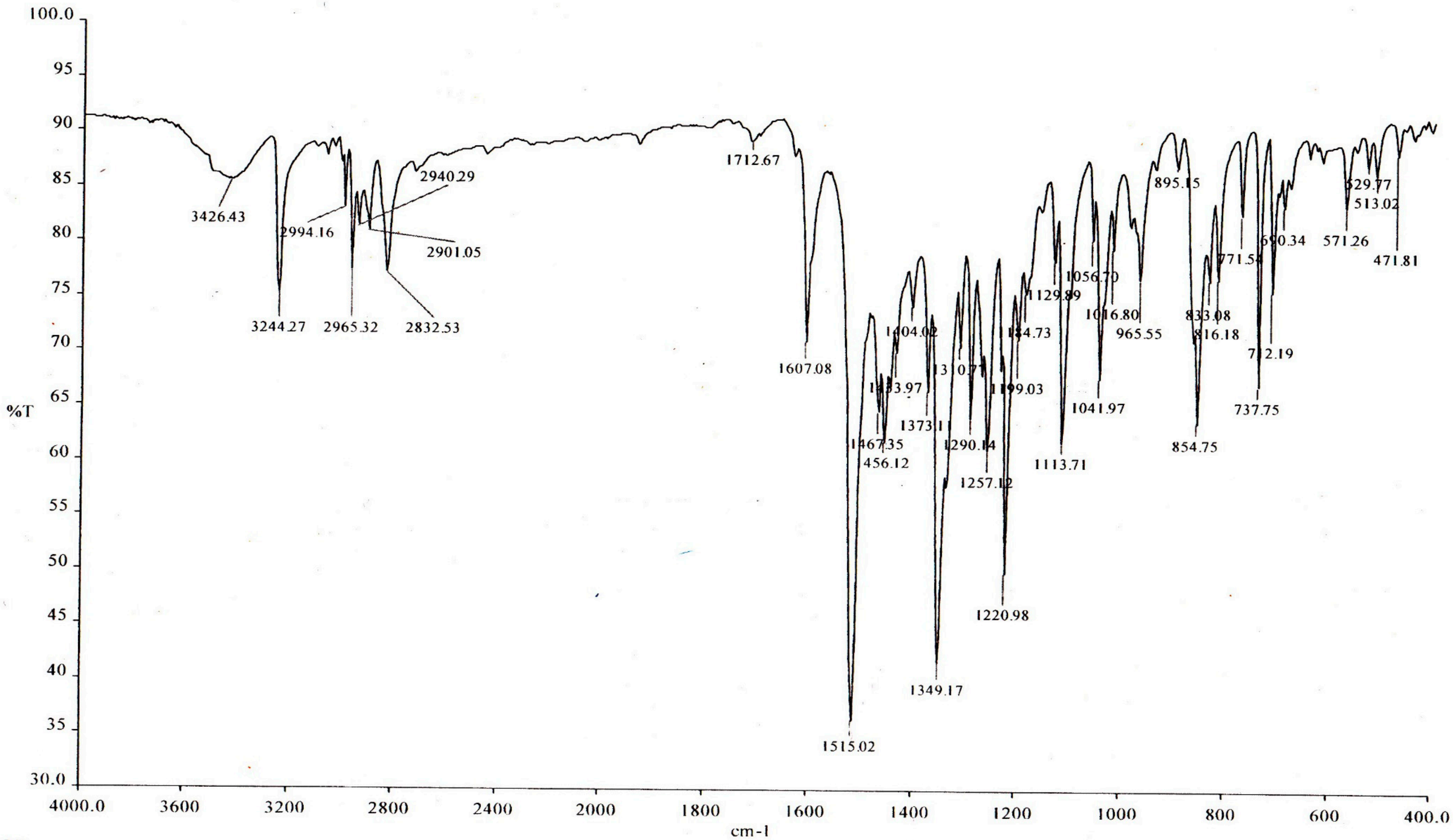

F-27

Figure S43. IR spectrum of compound **3m**.

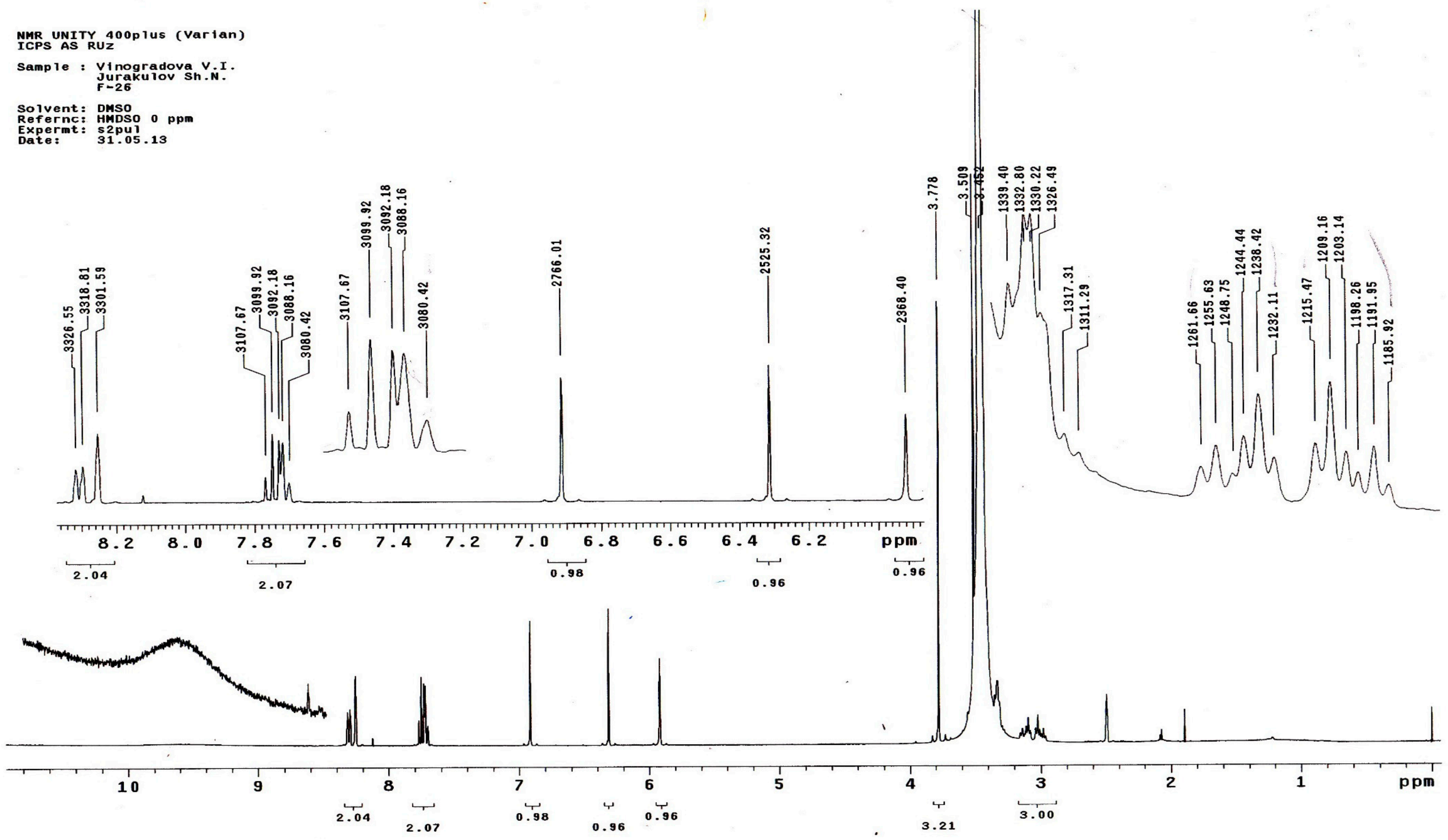

Figure S44. ^{1}H NMR spectrum of compound **3n**.

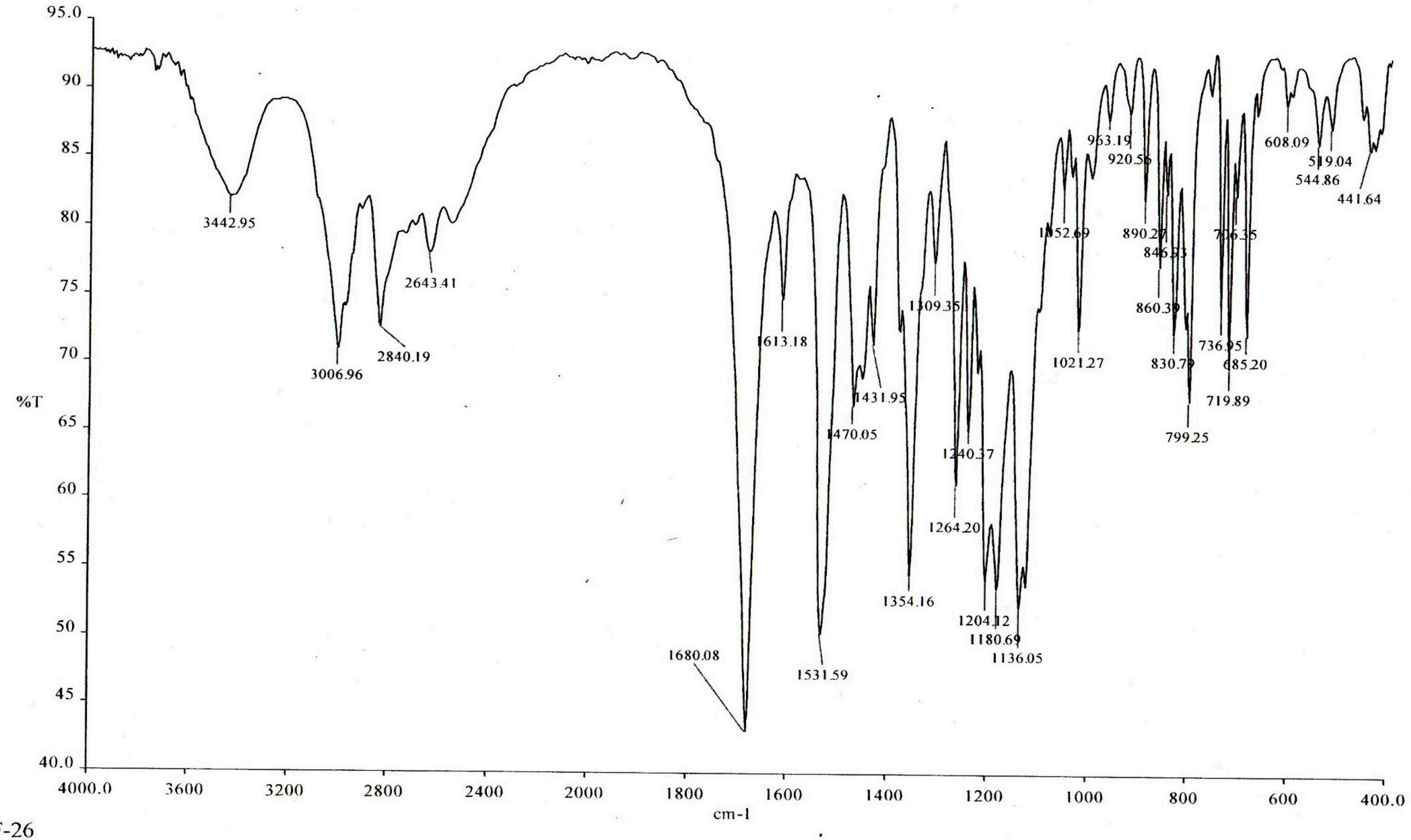

F-26

Figure S45. IR spectrum of compound **3n**.

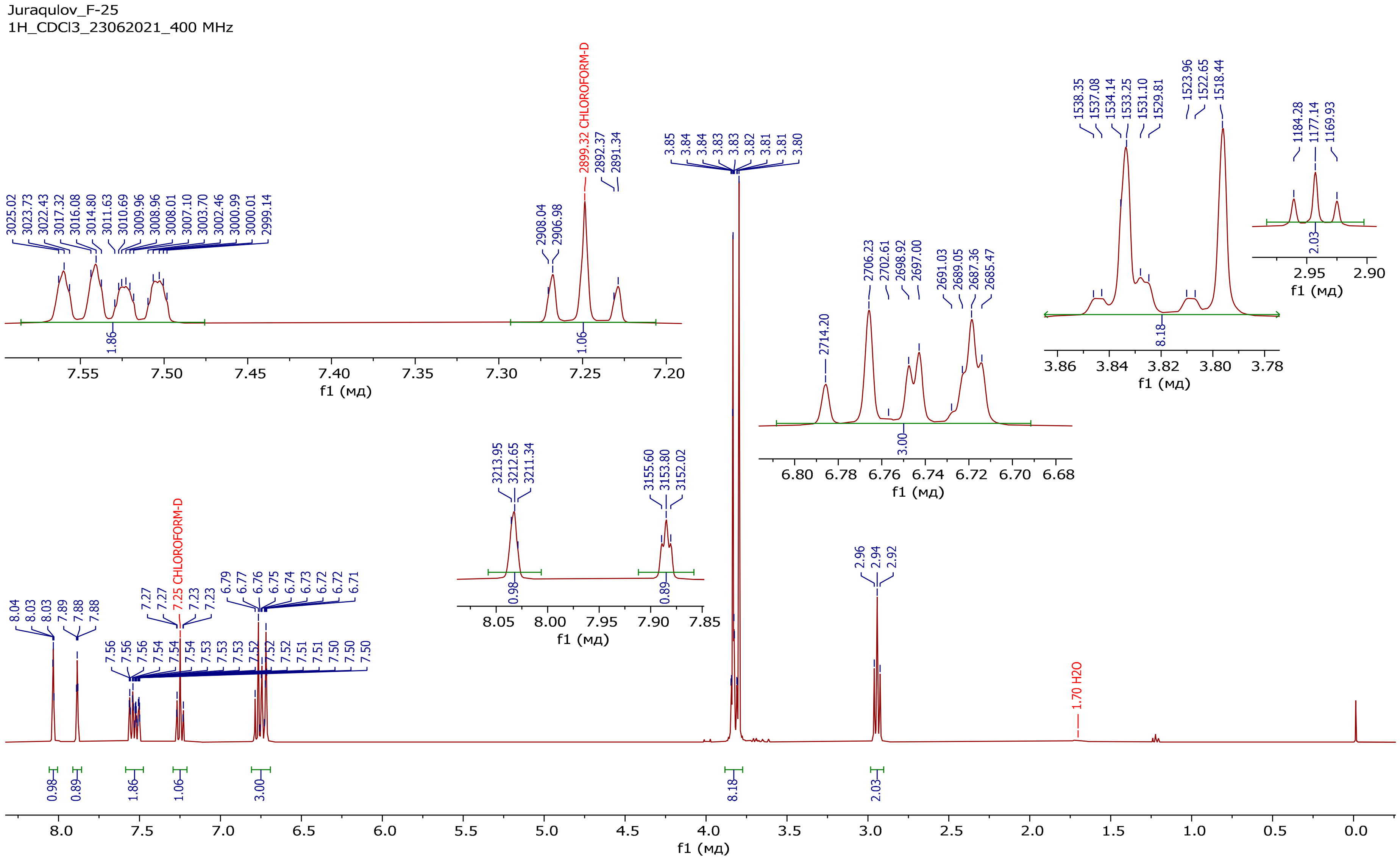

Figure S46. ¹H NMR spectrum of compound **3o**.

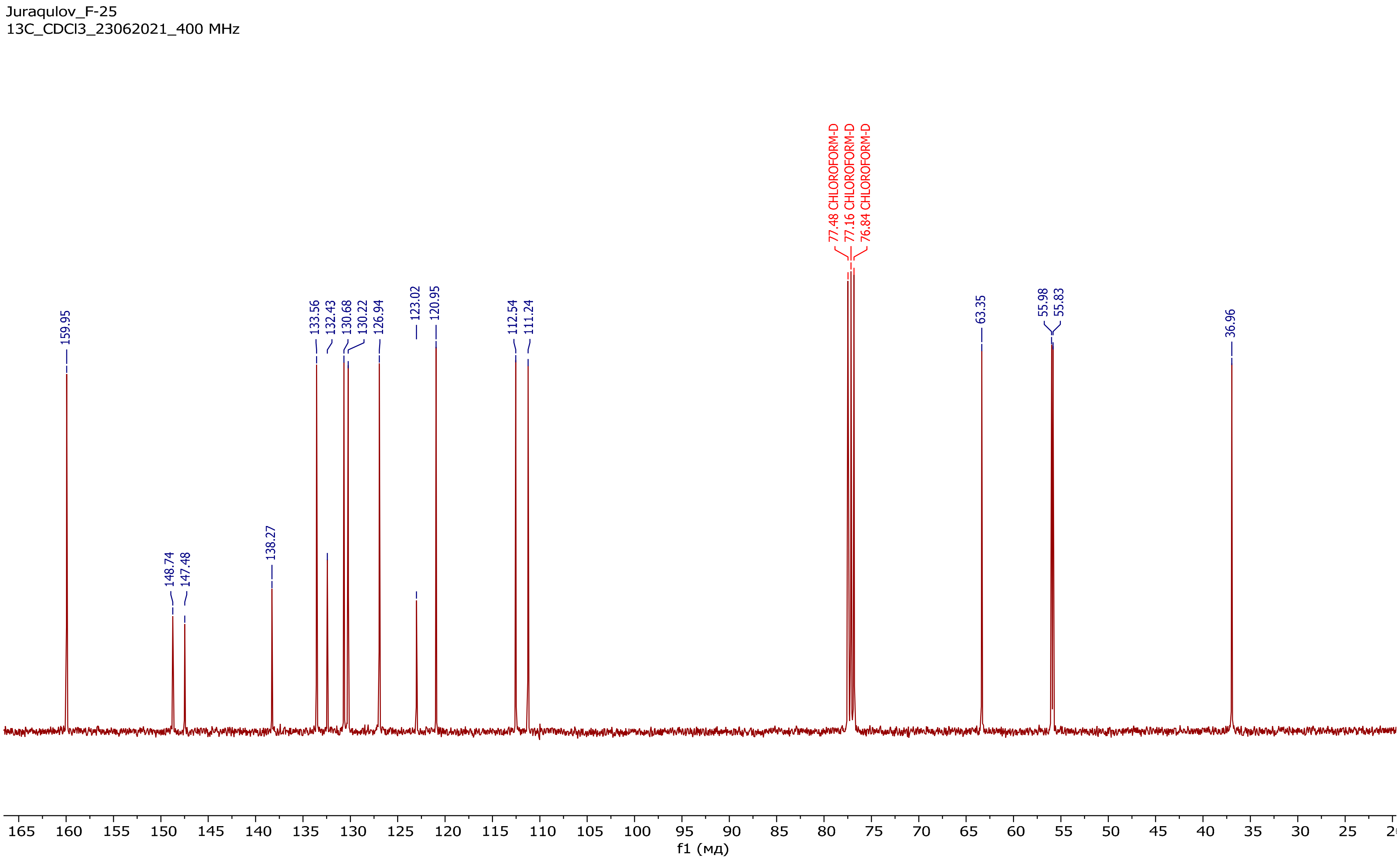

Figure S47. ^{13}C NMR spectrum of compound **3o**.

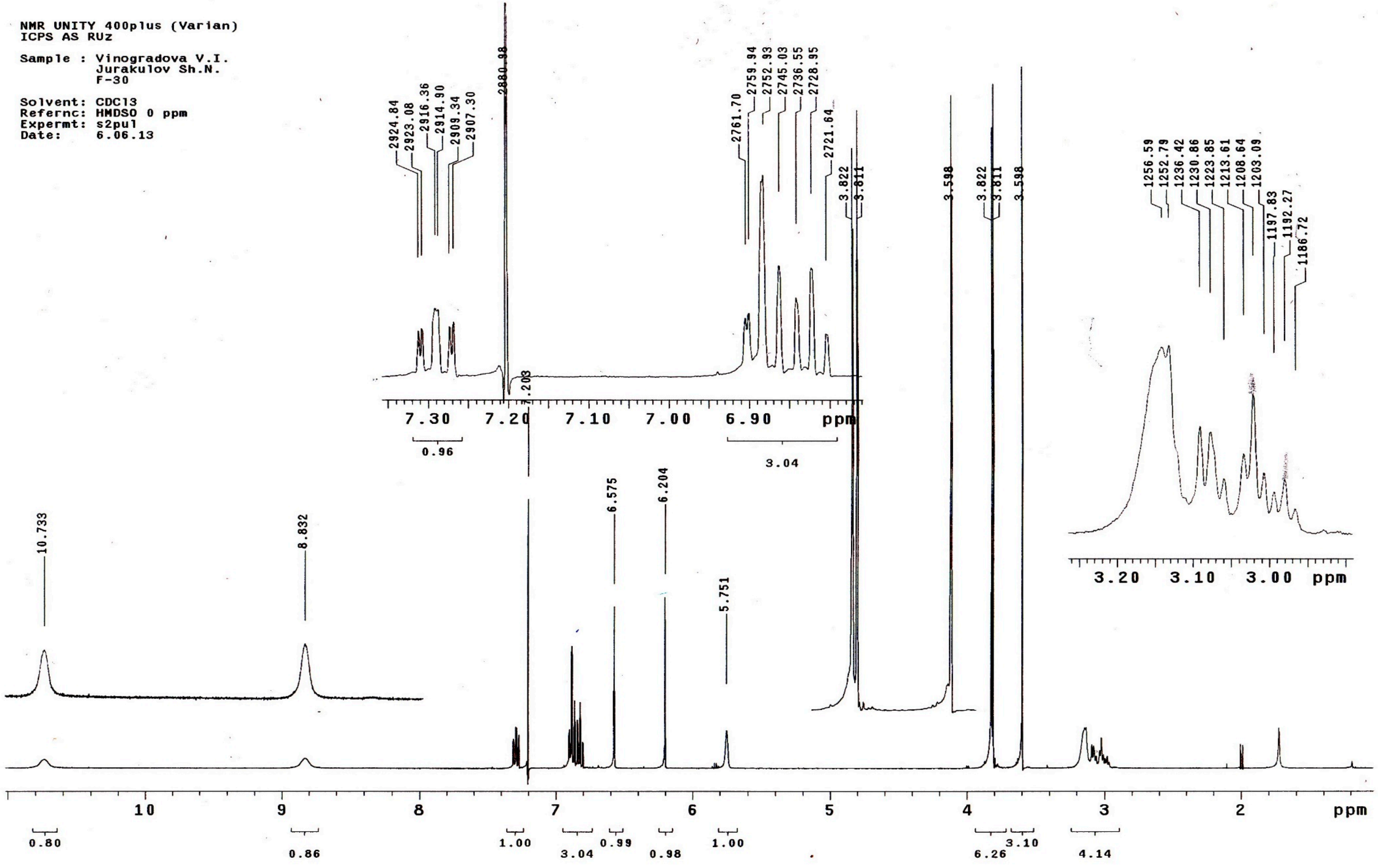

Figure S48. ^{1}H NMR spectrum of compound **3p**.

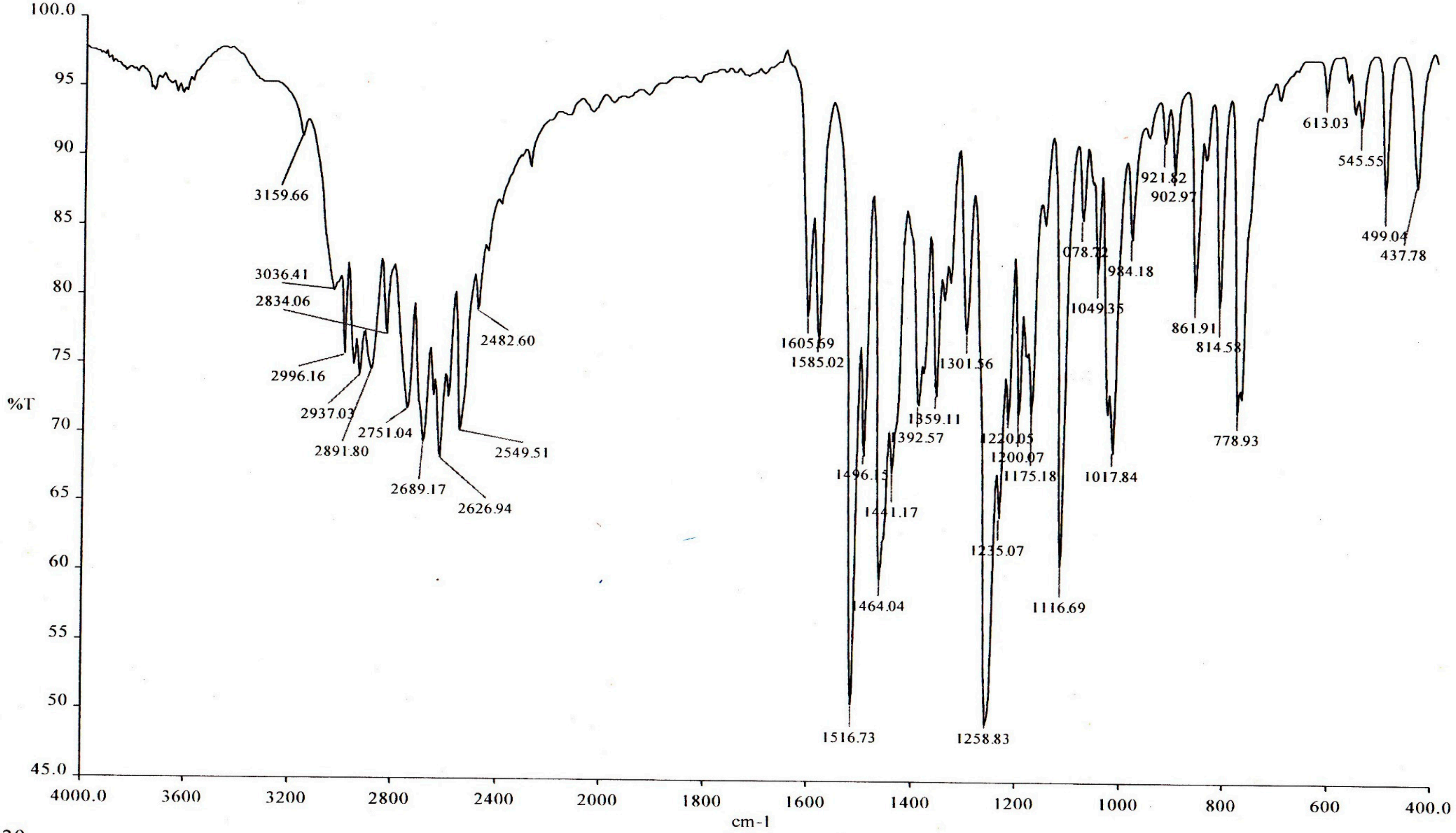

F-30

Figure S49. IR spectrum of compound **3n**.

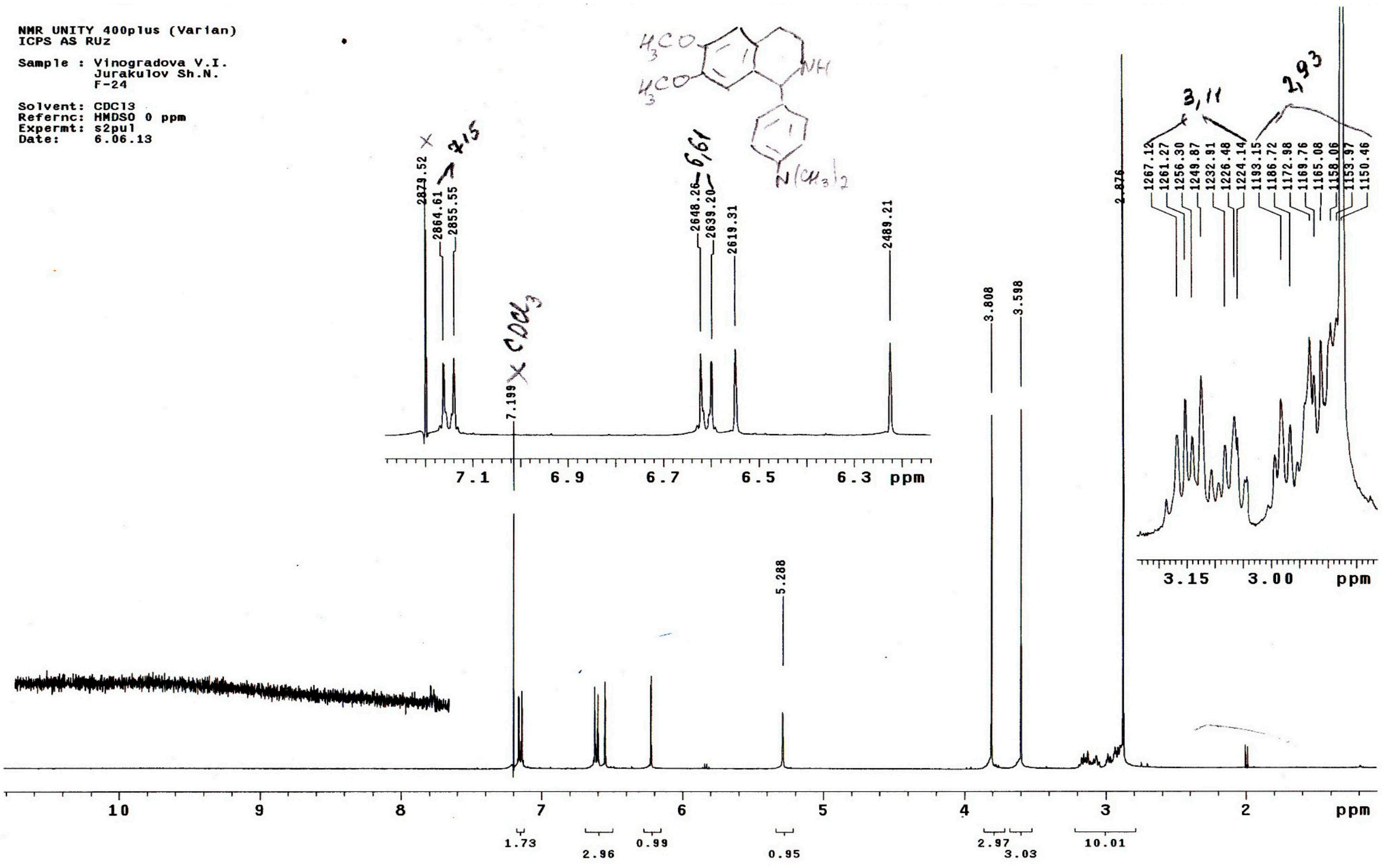

Figure S50. ^{1}H NMR spectrum of compound **3q**.

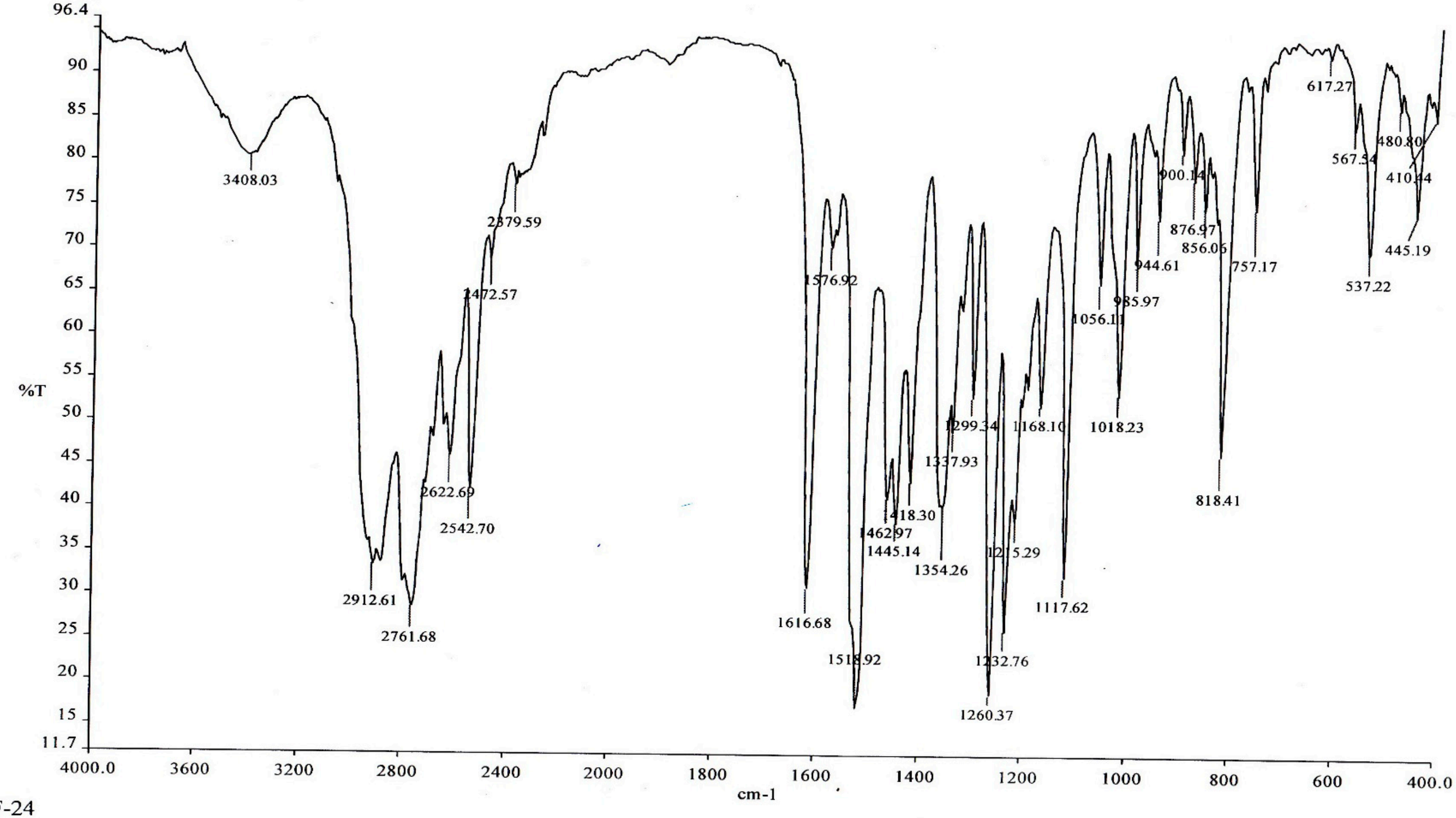

F-24

Figure S51. IR spectrum of compound **3q**.

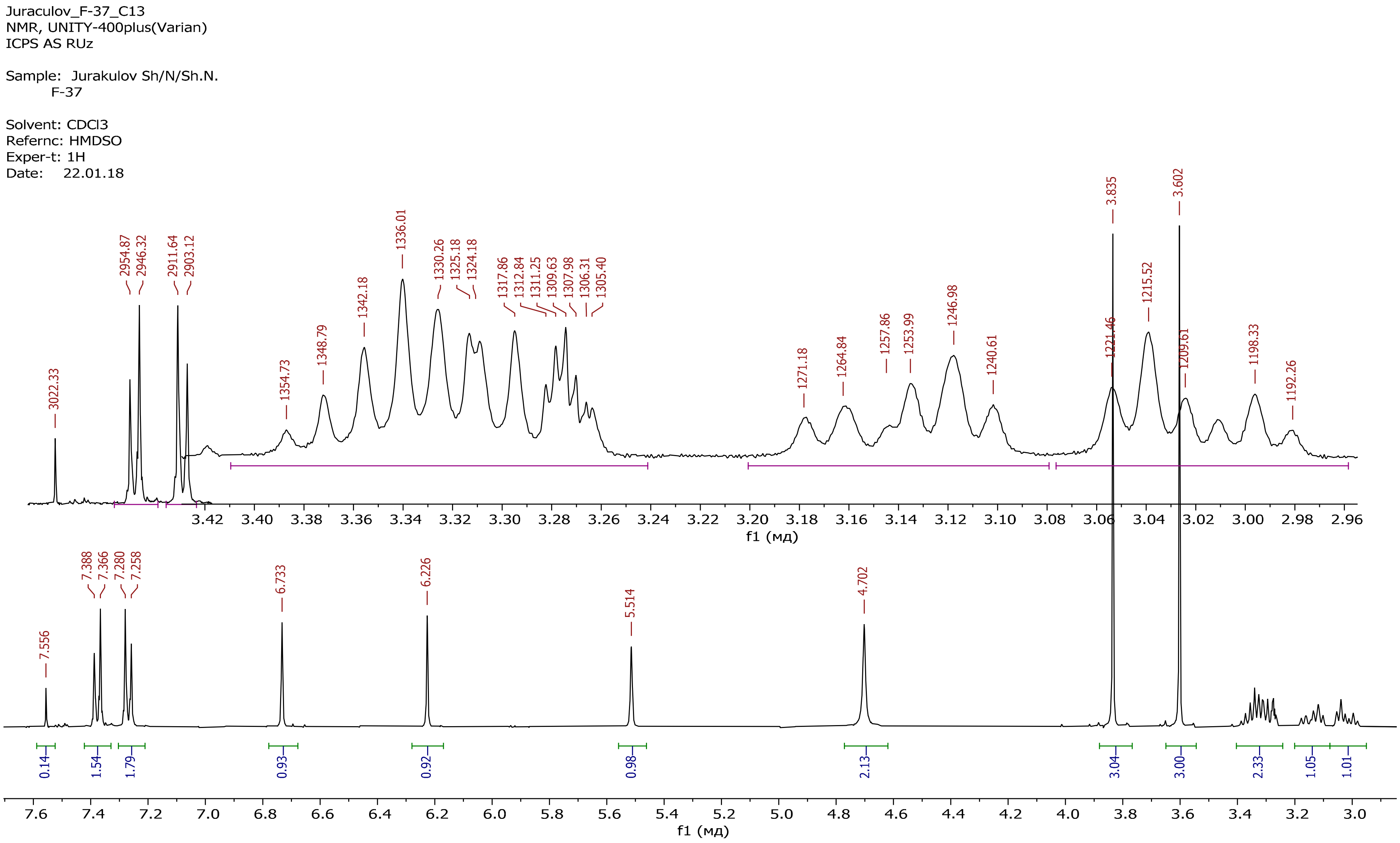

Figure S52. ^{1}H NMR spectrum of compound **3r**.

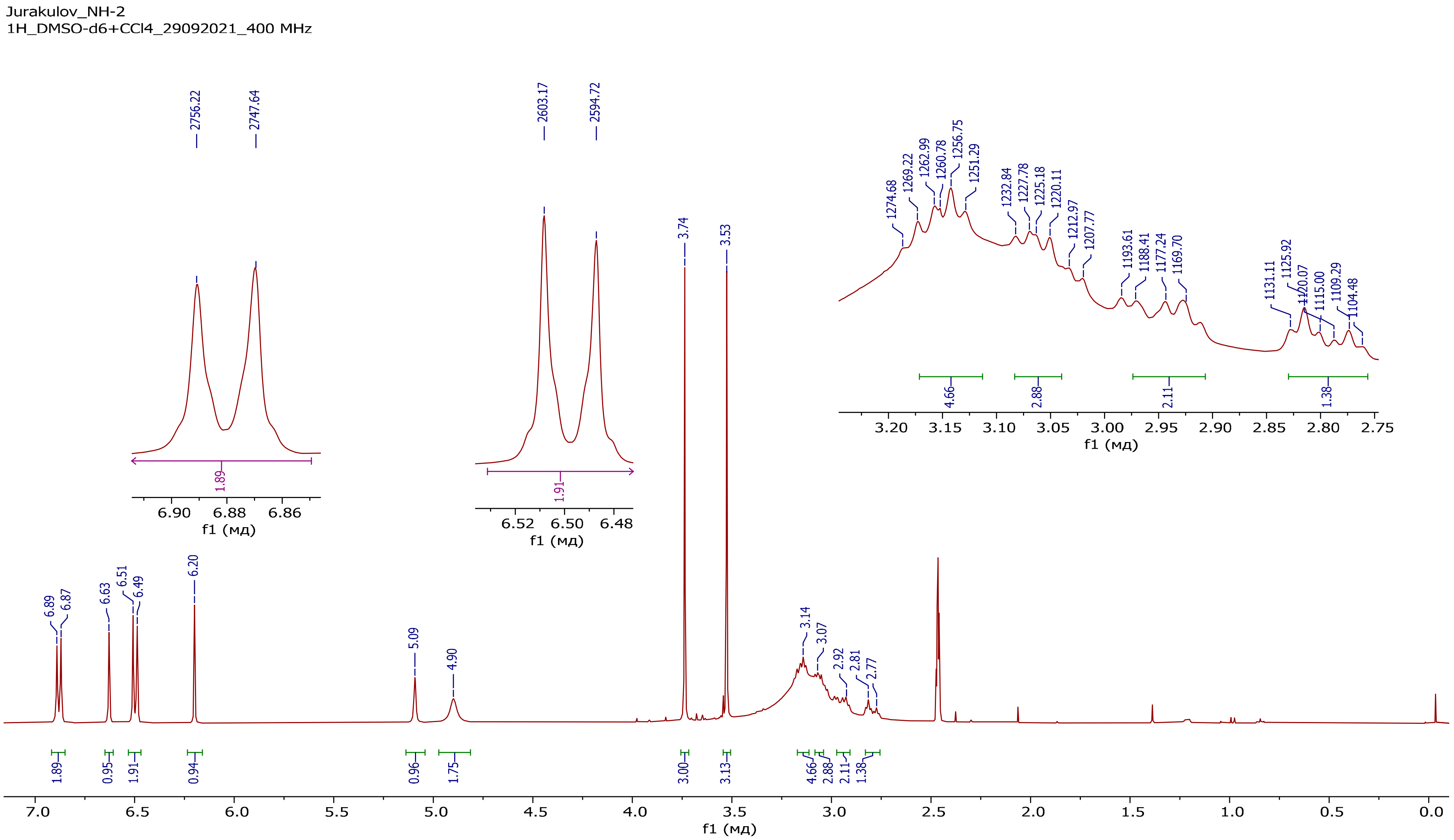

Figure S53. ^{1}H NMR spectrum of compound **4a**.

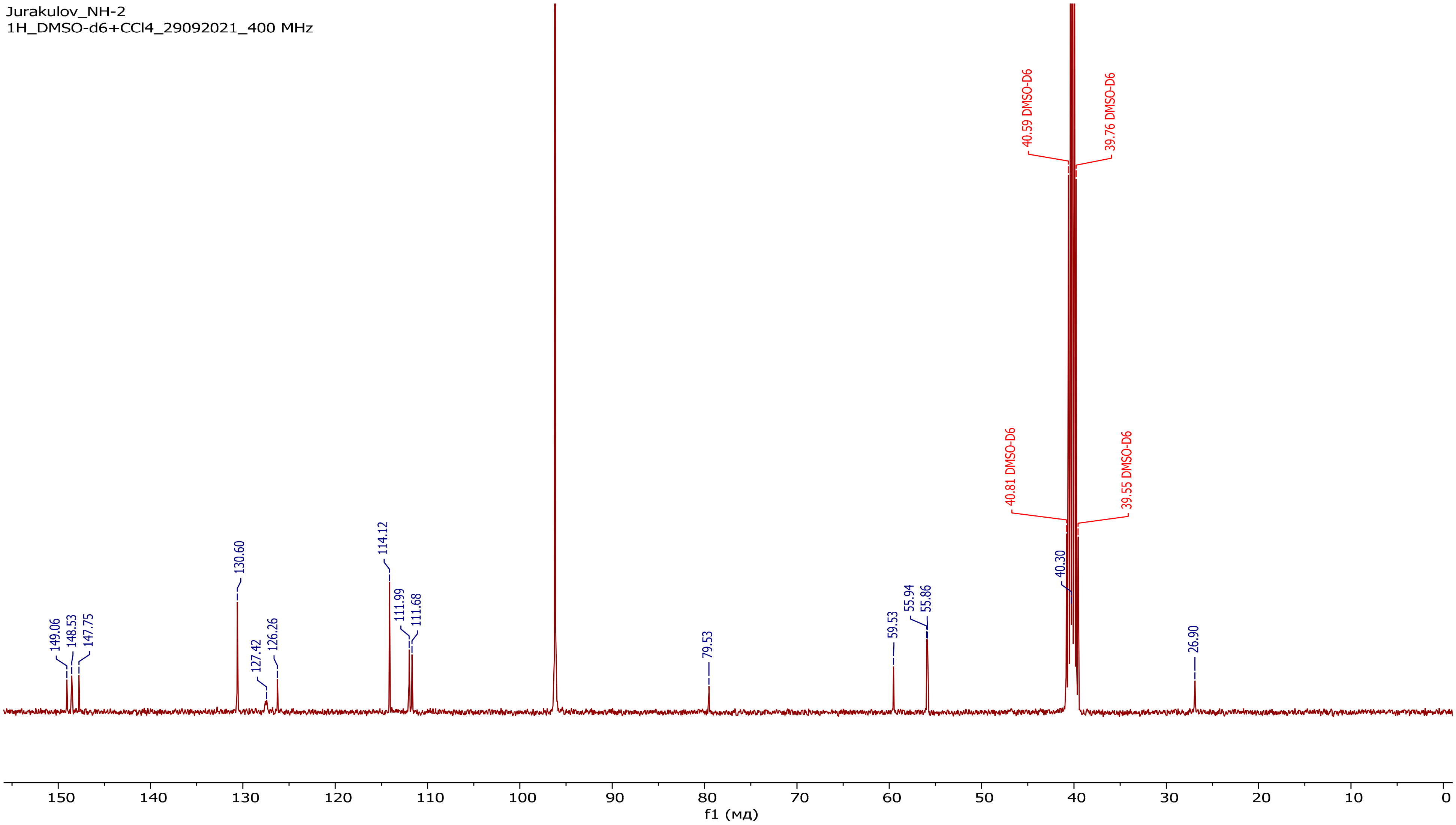

Figure S54. ^{13}C NMR spectrum of compound **4a**.

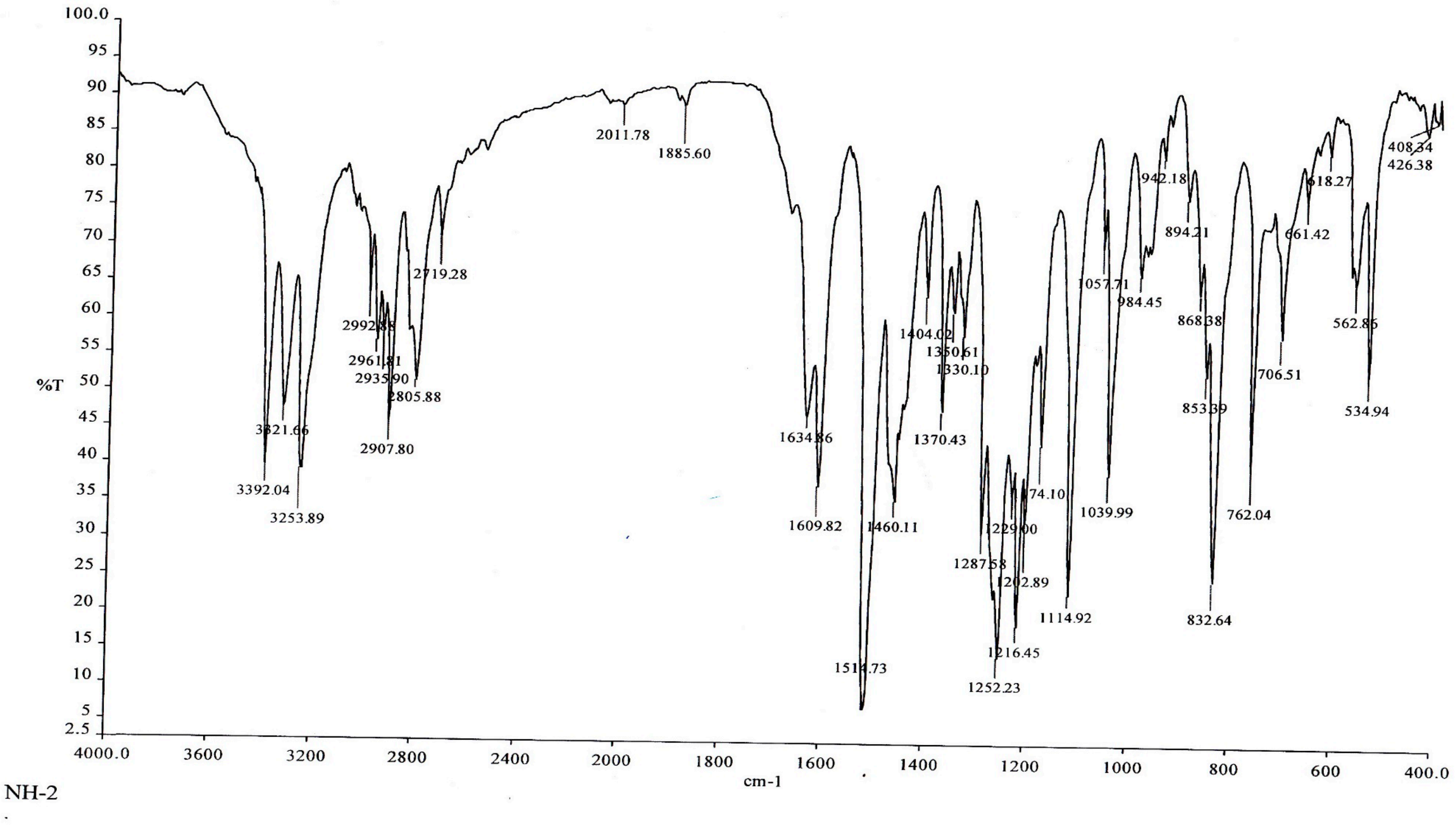

Figure S55. IR spectrum of compound **4a**.

Jurakulov_NH-1 — 1H_DMSO-d6+CCl4_29092021_400 MHz

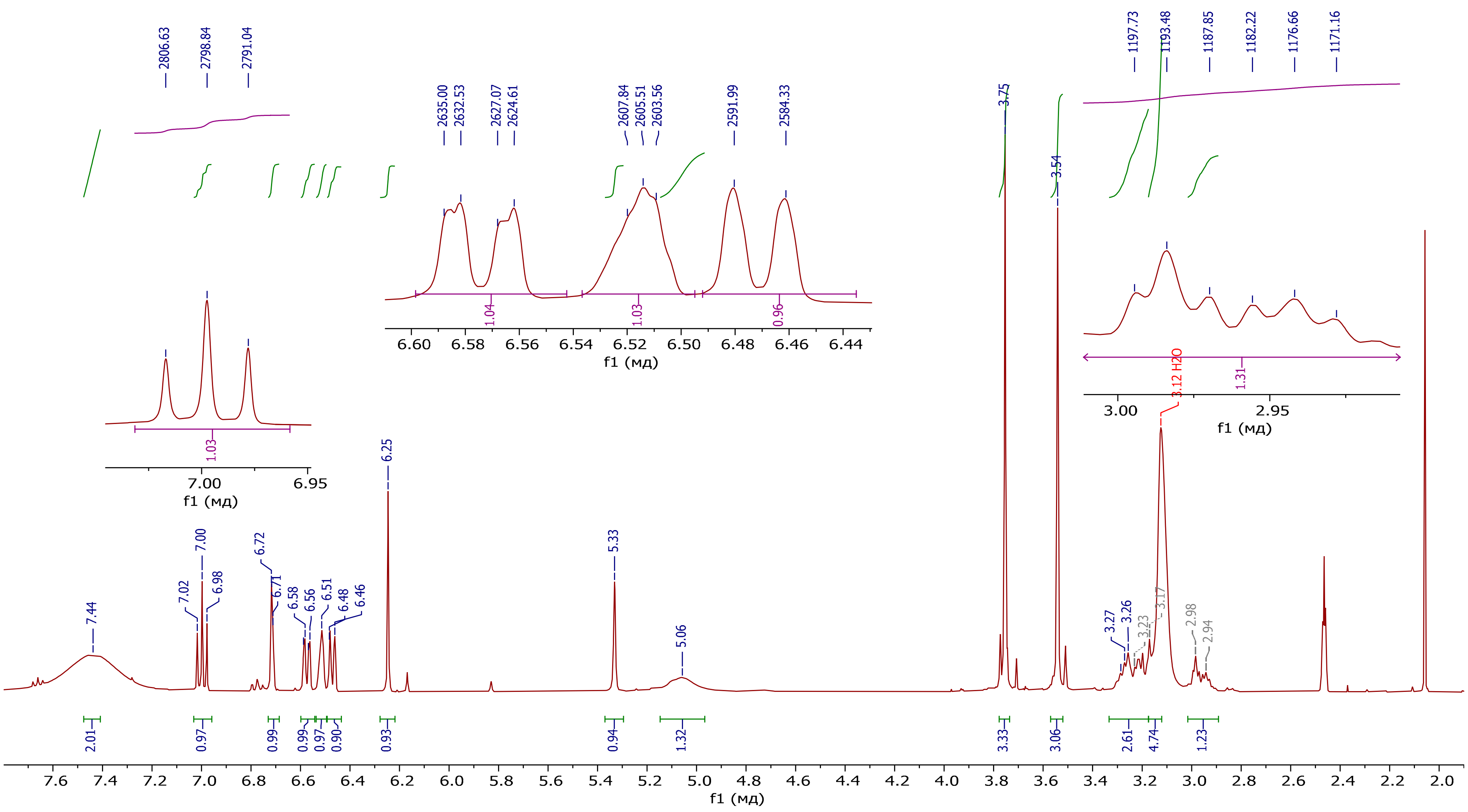

Figure S56. ^{1}H NMR spectrum of compound **4b**.

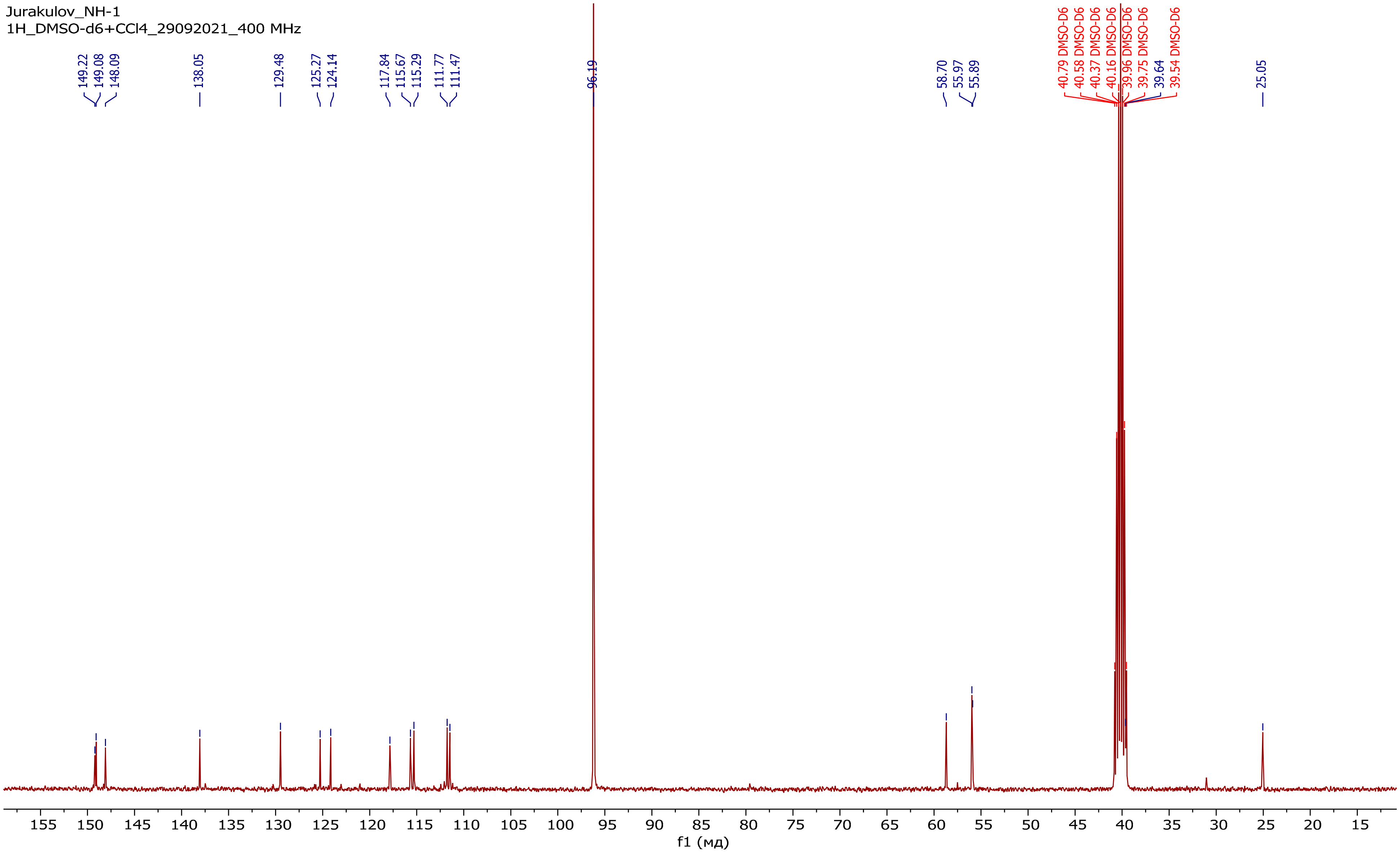

Figure S57. ^{13}C NMR spectrum of compound **4b**.
